# Hepatic FGF21 is not required for fasting metabolism but guides protein appetite post energy depletion

**DOI:** 10.1038/s44319-026-00790-9

**Published:** 2026-04-27

**Authors:** Justine Bruse, Clothilde Marbach, Arnaud Polizzi, Tatiana Landre, Juliette Salvi, Valérie Alquier-Bacquie, Shiou-Ping Chen, Marine Huillet, Clémence Rives, Céline M P Martin, Prunelle Perrier, Fadila Benhamed, Marion Régnier, Stefan Weger, Claire Naylies, Yannick Lippi, Caroline Sommer, Mikael Albin, Frédéric Lasserre, Thierry Levade, Michael Schupp, Laurence Gamet-Payrastre, Léon Kautz, Nicolas Loiseau, Walter Wahli, Sandrine Ellero-Simatos, Céline Cruciani-Guglielmacci, Alexandra Montagner, Alexandre Benani, Catherine Postic, Hervé Guillou, Anne Fougerat

**Affiliations:** 1 Toxalim (Research Center in Food Toxicology), INRAE UMR 1331, ENVT, INP-Purpan, Université de Toulouse, 31300 Toulouse, France; 2 Service de Gastroentérologie, Hépatologie, Nutrition et Maladies Héréditaires du Métabolisme pédiatriques, Hôpital des Enfants, CHU de Toulouse, 31059 Toulouse, France; 3 Centre des Sciences et du Goût et de l’Alimentation (CSGA), UMR CNRS 6265, INRAE 1324, Institut Agro, Université de Bourgogne, 21000 Dijon, France; 4 Université Paris Cité, Unité de Biologie Fonctionnelle et Adaptative (BFA), CNRS, UMR8251, 75013 Paris, France; 5 Université de Paris Cité, Institut Cochin, CNRS, INSERM, 75014 Paris, France; 6 Charité-Universitätsmedizin Berlin, Freie Universität Berlin, Humboldt-Universität zu Berlin, Institute of Virology, Campus Benjamin Franklin, Berlin, Germany; 7 Univ Toulouse, INSERM, Cancer Research Center of Toulouse (CRCT), 31100 Toulouse, France; 8 Laboratoire de Biochimie, Institut Fédératif de Biologie, CHU Purpan, 31059 Toulouse, France; 9 Charité-Universitätsmedizin Berlin, Freie Universität Berlin, Humboldt-Universität zu Berlin, Institute of Pharmacology, Cardiovascular Metabolic Renal (CMR)-Research Center, Berlin, Germany; 10 Institut de Recherche en Santé Digestive (IRSD), INSERM U1220, Université de Toulouse, INRAE, ENVT, 31300 Toulouse, France; 11 Center for Integrative Genomics, Université de Lausanne, CH-1015 Lausanne, Switzerland; 12 Institut des Maladies Métaboliques et Cardiovasculaires (I2MC), INSERM, 31400 Toulouse, France

**Keywords:** Metabolism, Neuroscience, Signal Transduction

## Abstract

Fasting initiates a coordinated metabolic response to preserve energy balance. As glycogen stores are depleted, the body transitions to mobilizing fatty acids from adipose tissue and generating ketone bodies in the liver to sustain the function of vital organs. A network of hormonal signals and transcriptional programs coordinate these adaptations. Among these, the hepatokine fibroblast growth factor 21 (FGF21) is strongly upregulated during fasting and has been proposed as a key mediator of the fasting response. To investigate the physiological functions of FGF21, we study mice with hepatocyte-specific deletion of *Fgf21*. Although the liver is the primary source of circulating FGF21 during fasting, its absence in hepatocytes does not alter typical fasting-induced gene expression or key metabolic pathways such as hepatic gluconeogenesis, adipose tissue lipolysis, or ketone production. Instead, we uncover a distinct role for FGF21 in promoting protein appetite following a fast. These findings challenge the conventional view of hepatocyte-produced FGF21 as a fasting-acting hormone and reveal a more specialized function in guiding nutrient selection after energy depletion.

## Synopsis

Despite being strongly induced by fasting, hepatocyte-derived FGF21 is not required for the core metabolic responses to fasting. Instead, our data identify hepatocyte-derived FGF21 as a critical regulator of nutrient preference following fasting, specifically promoting protein appetite.

- Hepatocyte FGF21 is not required for key metabolic adaptations to fasting.
- Fasting-induced changes in hepatic gene expression and metabolome are independent of hepatocyte FGF21.
- Hepatocyte FGF21 deletion does not influence adipose tissue responses to fasting.
- Hepatocyte FGF21 drives protein preference following fasting.

## Abstract (web summary)

Despite being strongly induced by fasting, hepatocyte-derived FGF21 is not required for the core metabolic responses to fasting. Instead, our data identify hepatocyte-derived FGF21 as a critical regulator of nutrient preference following fasting, specifically promoting protein appetite.

## Graphical abstract

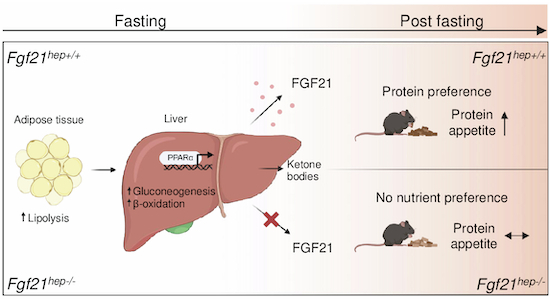

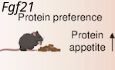

## Introduction

Adaptation to fluctuations in nutrient availability is fundamental to the survival of the organism and its metabolic health. In mammals, fasting triggers a complex physiological response that involves coordinated transcriptional and metabolic reprogramming across multiple tissues, including the liver (Fougerat et al, 2024) and white adipose tissue (WAT) (Ruppert and Kersten, 2025). These adaptations serve to maintain energy homeostasis by shifting metabolic priorities from glucose utilization toward lipid mobilization and ketogenesis (Puchalska and Crawford, 2017). They are driven by hormonal changes and nutrient-sensing transcriptional regulators (Goldstein and Hager, 2015) such as peroxisome proliferator-activated receptor alpha (PPARα) (Kersten et al, 1999; Montagner et al, 2016), cAMP response element-binding protein (CREB) (Herzig et al, 2001; Altarejos and Montminy, 2011), glucocorticoid receptor (GR), and forkhead box protein O1 (FOXO1) (Zhang et al, 2006; Haeusler et al, 2014). The liver plays a central role in these processes, acting both as a hub and as a source of metabolic and endocrine signals that influence the function of peripheral tissues (Fougerat et al, 2024).

One of the most prominently induced hepatic genes during fasting is fibroblast growth factor 21 (*Fgf21*), encoding for the liver-derived hormone or hepatokine FGF21 (Inagaki et al, 2007; Badman et al, 2007; Gälman et al, 2008). Hepatocyte FGF21 expression is strongly upregulated in response to fasting in a PPARα-dependent manner (Montagner et al, 2016; Inagaki et al, 2007; Badman et al, 2007; Lundåsen et al, 2007). This rise in FGF21 expression and plasma levels originates from hepatocytes (Montagner et al, 2016; Markan et al, 2014) and occurs in response to fasting-induced adipose tissue lipolysis (Jaeger et al, 2015; Fougerat et al, 2022). Early studies suggested that FGF21 functions as a key effector of the metabolic fasting response, promoting ketogenesis, increasing fatty acid oxidation (Inagaki et al, 2007; Badman et al, 2007, 2009; Potthoff et al, 2009) and maintaining glucose homeostasis (Liang et al, 2014). Despite these proposed roles, the specific physiological significance of hepatocyte FGF21 during fasting remains incompletely understood because most of these studies were performed using whole-body FGF21 knockout mice. In this context, it is worth mentioning that FGF21 is also expressed in many tissues, such as the WAT (Dutchak et al, 2012), the brown adipose tissue (BAT) (Fisher et al, 2012) and the exocrine pancreas (Coate et al, 2017). Moreover, while it was initially considered to be a starvation hormone, liver FGF21 has now been reported to be more broadly regulated by many forms of metabolic stress such as high carbohydrates or alcohol consumption (Talukdar et al, 2016; Jensen-Cody et al, 2020; Iroz et al, 2017; Song et al, 2018), ketogenic diet (Badman et al, 2007; Song et al, 2018), and protein restriction (Laeger et al, 2014; Solon-Biet et al, 2023).

Although pharmacological or transgenic overexpression of FGF21 improves metabolic outcomes in models of obesity and insulin resistance (Kliewer and Mangelsdorf, 2019), these effects may not accurately reflect the endogenous role of FGF21 under physiological fasting conditions. In the present study, we conducted a comprehensive analysis of the genomic and metabolic responses to fasting in wild-type mice and mice with hepatocyte-restricted deletion of *Fgf21*. Changes of gene expression and metabolic profiling in the liver, WATs, and BAT were assessed. Surprisingly, while fasting induced significant changes in gene expression, we found that the absence of hepatocyte FGF21 had minimal impact on fasting-induced remodeling across these tissues. Instead, we show that hepatocyte FGF21 is required for protein preference following fasting. Our findings challenge the classical concept of hepatocyte-produced FGF21 as a fasting-acting hormone and reveal its role as a key endocrine signal guiding nutrient selection after food deprivation.

## Results

### Hepatocyte FGF21 is dispensable for fasting-induced hypoglycemia and ketone body production

Several studies have suggested that liver-derived FGF21 plays a crucial role in the metabolic responses to fasting (Inagaki et al, 2007; Potthoff et al, 2009; Badman et al, 2007; Liang et al, 2014). However, most of these reports were based on observations in global *Fgf21* knockout or FGF21 overexpression models, which may not reflect the specific contribution of endogenous hepatic FGF21.

**Figure 1:**
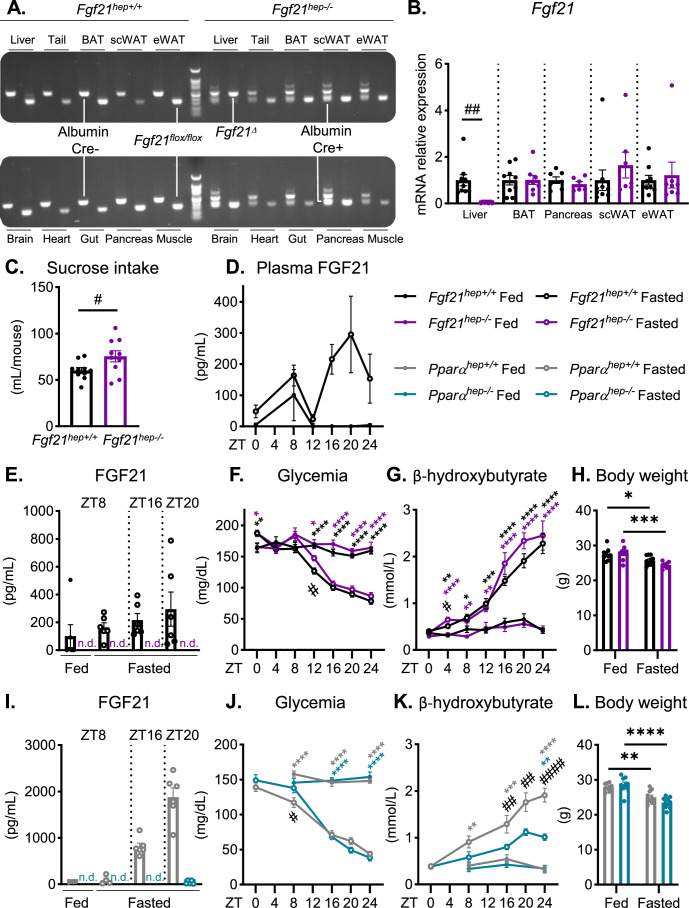
Characterization of the hepatocyte-specific fibroblast growth factor 21 (FGF21) knockout mouse model. (**A**) PCR analysis of *Fgf21* floxed (*Fgf21*^*hep+/+*^) and *Albumin-Cre* (*Albumin-Cre*^*+/−*^) genes from male mice that are liver floxed (*Fgf21*^*hep+/+*^) or liver knockout (*Fgf21*^*hep−/−*^*)* for *Fgf21* using DNA extracted from different organs. (**B**) mRNA relative expression of *Fgf21* in liver, brown adipose tissue (BAT), pancreas, subcutaneous white adipose tissue (scWAT), epididymal white adipose tissue (eWAT) samples of *Fgf21*^*hep+/+*^ and *Fgf21*^*hep−/−*^ male mice measured by qRT-PCR (*n* = 6–9 mice per group, biological replicates, Student’s *t*-test, Liver *Fgf21*, *p* = 0.0036). (**C**) Sucrose intake during a two-bottle choice of 10% sucrose vs water for 4 days, in *Fgf21* liver floxed (*Fgf21*^*hep+/+*^) or liver knockout (*Fgf21*^*hep−/−*^*)* male mice (*n* = 10 mice per group, biological replicates, Student’s *t*-test, *p* = 0.0364). (**D**) Liver floxed (*Fgf21*^*hep+/+*^) for *Fgf21* male mice were fed *ad libitum* or fasted for 24 h, and blood was collected at ZT0, ZT8, ZT12, ZT16, ZT20, and ZT24. FGF21 plasma level was determined by ELISA (*n* = 6 mice per group, biological replicates with repeated measurements taken at each ZT). (**E**–**H**) *Fgf21* liver floxed (*Fgf21*^*hep+/+*^) or liver knockout (*Fgf21*^*hep−/−*^*)* male mice were fed *ad libitum* or fasted for 24 h, and blood was collected at ZT8, ZT16, and ZT20. (**E**) FGF21 plasma level was determined by ELISA (*n* = 6 mice per group, biological replicates with repeated measurements taken at each ZT). (**F**) Blood glucose levels were monitored from ZT0 to ZT24 (*n* = 8–10 mice per group, biological replicates with repeated measurements taken at each ZT, two-way ANOVA followed by Šídák’s multiple comparisons test α = 0.05, Fed vs Fasted in *Fgf21*^*hep+/+*^ or *Fgf21*^*hep−/−*^ ZT0, *p_adj_ = 0.0328; **p_adj_ = 0.0094; ZT12, *p_adj_ = 0.0115, ****p_adj_ < 0.0001; ZT16-20-24, ****p_adj_ < 0.0001; Fasted *Fgf21*^*hep+/+*^ vs Fasted *Fgf21*^*hep−/−*^ ZT12, #*p*_adj_ = 0.015). (**G**) β-hydroxybutyrate levels were monitored from ZT0 to ZT24 (*n* = 8–10 mice per group, biological replicates with repeated measurements taken at each ZT, two-way ANOVA followed by Šídák’s multiple comparisons test α = 0.05, Fed *vs* Fasted in *Fgf21*^*hep+/+*^ or *Fgf21*^*hep−/−*^ ZT4, **p_adj_ = 0.029, ****p_adj_ < 0.0001; ZT8, *p_adj_ = 0.0445, **p_adj_ = 0.0097; ZT12, ***p_adj_ = 0.0002, **p_adj_ = 0.0089; ZT16-20-24, ****p_adj_ < 0.001; Fasted *Fgf21*^*hep+/+*^ vs Fasted *Fgf21*^*hep−/−*^ ZT4, #*p*_adj_ = 0.0467). (**H**) Body weight was measured after 24 h fasting (*n* = 8–10 mice per group, biological replicates, two-way ANOVA followed by Šídák’s multiple comparisons test α = 0.05, Fed vs Fasted in *Fgf21*^*hep+/+*^ or *Fgf21*^*hep−/−*^, *p_adj_ = 0.0395, ***p_adj_ = 0.0003). (**I**–**L**) *Pparα* liver floxed (*Pparα*^*hep+/+*^) or *Pparα* liver knockout (*Pparα*^*hep−/−*^) mice were fed *ad libitum* or fasted for 24 h, and blood was collected at ZT8, ZT16, and ZT20. (**I**) FGF21 plasma level was determined by ELISA (*n* = 6 mice per group, biological replicates with repeated measurements taken at each ZT). (**J**) Blood glucose levels were monitored from ZT0 to ZT24 (*n* = 8–10 mice per group, biological replicates with repeated measurements taken at each ZT, fed groups: measurements taken only at ZT8, ZT16, ZT24, two-way ANOVA followed by Šídák’s multiple comparisons test, α = 0.05, at ZT8, ZT16, and ZT24, Student’s *t*-test at ZT0 and ZT20 between Fasted *Pparα*^*hep+/+*^ vs Fasted *Pparα*^*hep−/−*^; Fed vs Fasted in *Pparα*^*hep+/+*^ or *Pparα*^*hep−/−*^ ZT8-16-24, ****p_adj_ < 0.0001; Fasted *Pparα*^*hep+/+*^ vs Fasted *Pparα*^*hep-/-*^ ZT8, #*p*_adj_ = 0.0348). (**K**)β-hydroxybutyrate levels were monitored from ZT0 to ZT24 (*n* = 8–10 mice per group, biological replicates with repeated measurements taken at each ZT, fed groups: measurements taken only at ZT8, ZT16, and ZT24, two-way ANOVA followed by Šídák’s multiple comparisons test, α = 0.05, at ZT8, ZT16, and ZT24, Student’s *t*-test at ZT0 and ZT20 between Fasted *Pparα*^*hep+/+*^ vs Fasted *Pparα*^*hep−/−*^; Fed vs Fasted in *Pparα*^*hep+/+*^ or *Pparα*^*hep−/−*^ ZT8, **p_adj_ = 0.0033; ZT16 ***p_adj_ = 0.0001; ZT24, **p_adj_ = 0.0013, ****p_adj_ < 0.0001; Fasted *Pparα*^*hep+/+*^ vs Fasted *Pparα*^*hep−/−*^ ZT8, ##*p*_adj_ = 0.008; ZT20, ##*p* = 0.007, ZT24, ####*p*_adj_ < 0.0001). (**L**) Body weight was measured after 24 h fasting (*n* = 8–10 mice per group, biological replicates, two-way ANOVA followed by Šídák’s multiple comparisons test α = 0.05, Fed vs Fasted in *Pparα*^*hep+/+*^ or *Pparα*^*hep−/−*^, **p_adj_ = 0.0023, ****p_adj_ < 0.0001). Data information: All data were presented as mean ± SEM; * shows a fasting effect; # shows a genotype effect. n.d. not detected, ZT Zeitgeber time. Source data are available online for this figure.

To investigate the physiological function of hepatocyte-produced FGF21, we generated mice with hepatocyte-specific deletion of *Fgf21* (*Fgf21*^*flox/flox*^*albumin-Cre*^*+/−*^, designated as *Fgf21*^*hep−/−*^) by crossing C57Bl/6 J *Fgf21*^*flox/flox*^ mice carrying LoxP sites flanking the three exons of the *Fgf21* gene with albumin-Cre mice on the same genetic background. We confirmed the specific deletion of *Fgf21* in the livers of adult *Fgf21*^*hep−/−*^ mice, indicating that most hepatic *Fgf21* expression is from hepatocytes (Fig. 1A,B). As prior studies have shown that FGF21 reduces sweet preference (Talukdar et al, 2016; Von Holstein-Rathlou et al, 2016; Iroz et al, 2017), we investigated whether the absence of hepatocyte FGF21 affects sucrose intake using the two-bottle preference assay (10% sucrose in drinking water *versus* water). Water consumption, which was measured daily for 3 days, confirmed that *Fgf21*^*hep−/−*^ mice consumed more sweetened water than *Fgf21*^*hep+/+*^ mice (Fig. 1C).

We next assessed the kinetics of plasma FGF21 levels in *Fgf21*^*hep+/+*^ mice fed *ad libitum* or fasted for 24 h. In agreement with previous reports (Montagner et al, 2016), we observed a peak of plasma protein expression at ZT8 in both fed and fasted groups during the light phase, corresponding to the sleep period of the animals. During the dark phase, plasma FGF21 concentrations increased and peaked at ZT20 only in fasted mice (Fig. 1D). Circulating FGF21 was not detected in *Fgf21*^*hep−/−*^ mice, confirming that the liver is the main source of plasma FGF21 (Markan et al, 2014) (Fig. 1E). The decrease in fasting blood glucose levels and the concomitant increase in ketone bodies starting from ZT12 were similar between *Fgf21*^*hep+/+*^ and *Fgf21*^*hep−/−*^ mice (Fig. 1F,G). The body weight loss was also comparable between genotypes after 24 h of fasting (Fig. 1H). Similar results were observed in fasted *Fgf21*^*hep+/+*^ and *Fgf21*^*hep−/−*^ females (Appendix Fig. S1A–D).

We also studied the effect of FGF21 deletion during a ketogenic diet, a condition that markedly induces FGF21 in the liver (Badman et al, 2007) (Appendix Fig. S2A). No significant difference in blood glucose, plasma ketone body levels, and body weight was observed between *Fgf21*^*hep+/+*^ and *Fgf21*^*hep−/−*^ mice fed a ketogenic diet for 9 days (Appendix Fig. S2B–D).

Liver FGF21 induction in response to starvation is highly controlled by the nuclear receptor PPARα in response to adipose tissue lipolysis (Badman et al, 2007; Inagaki et al, 2007; Montagner et al, 2016; Fougerat et al, 2022). Using mice with hepatocyte-specific deletion of *Pparα* (Montagner et al, 2016), we confirmed that fasting-induced FGF21 production was suppressed in the absence of PPARα in hepatocytes in both fed and fasted states (Fig. 1I). As expected, glycemia over a 24-h fasting challenge was unaffected by the absence of PPARα, whereas fasted hepatocyte *Pparα*-deficient mice (*Pparα*^*hep−/−*^) showed lower levels of plasma ketone bodies compared to fasted *Pparα*^*hep+/+*^ mice (Fig. 1J,K) (Régnier et al, 2018). This observation suggests that fasting ketogenesis depends on PPARα, but not on FGF21. Body weight was decreased in response to fasting in both genotypes (Fig. 1L).

These data confirmed that, during fasting, circulating FGF21 primarily originates from hepatocytes and is dependent on PPARα activity. However, *Fgf21* deletion in hepatocytes does not alter key metabolic adaptations to fasting such as hypoglycemia, ketogenesis, or weight loss.

### Hepatocyte FGF21 is dispensable for fasting-induced responses in the liver

**Figure 2:**
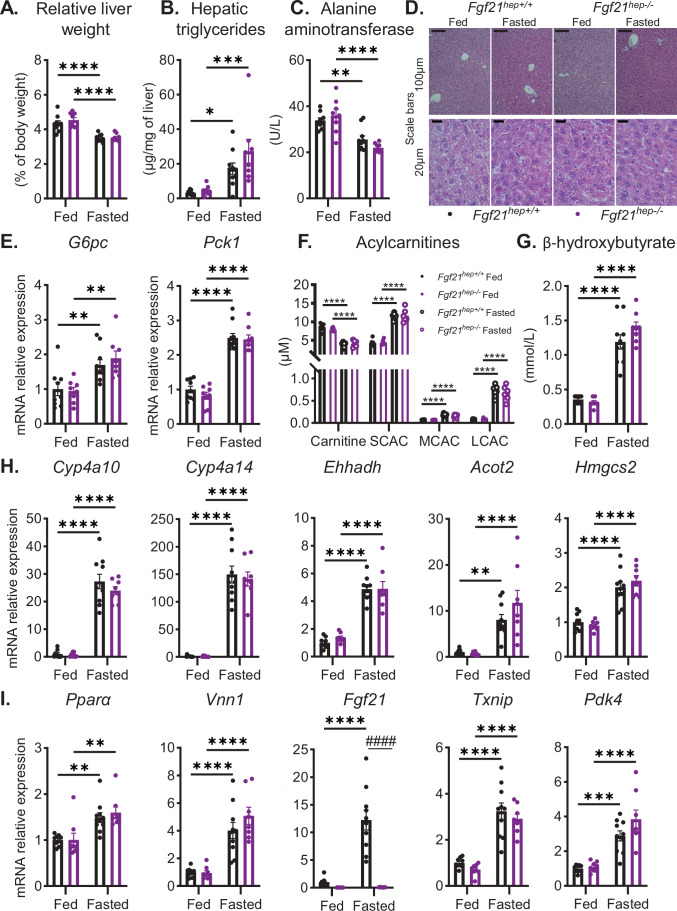
Hepatocyte-specific deletion of *Fgf21* does not affect fasting-induced hepatic responses. *Fgf21* liver floxed (*Fgf21*^*hep+/+*^) or *Fgf21* liver knockout (*Fgf21*^*hep−/−*^) mice were fed *ad libitum* or fasted for 20 h. (**A**) Relative liver weight (*n* = 8–10 mice per group, biological replicates, two-way ANOVA followed by Šídák’s multiple comparisons test α = 0.05, Fed *vs* Fasted in *Fgf21*^*hep+/+*^ or *Fgf21*^*hep−/−*^, ****p_adj_ < 0.0001). (**B**) Hepatic triglyceride levels (*n* = 8–10 mice per group, biological replicates, two-way ANOVA followed by Šídák’s multiple comparisons test α = 0.05, Fed vs Fasted in *Fgf21*^*hep+/+*^ or *Fgf21*^*hep−/−*^, *p_adj_ = 0.0182, ***p_adj_ = 0.005). (**C**) Plasma alanine aminotransferase levels (*n* = 8–10 mice per group, biological replicates, two-way ANOVA followed by Šídák’s multiple comparisons test α = 0.05, Fed vs Fasted in *Fgf21*^*hep+/+*^ or *Fgf21*^*hep−/−*^, **p_adj_ = 0.002, ****p_adj_ < 0.0001). (**D**) Representative pictures of H&E staining of liver sections. Scale bars, 100 and 20 µm. (**E**) mRNA relative expression of *G6pc and Pck1* in liver samples measured by qRT-PCR (*n* = 8–10 mice per group, biological replicates, two-way ANOVA followed by Šídák’s multiple comparisons test α = 0.05, *G6pc*, Fed vs Fasted in *Fgf21*^*hep+/+*^ or *Fgf21*^*hep−/−*^, **p_adj_ = 0.0094 (*Fgf21*^*hep+/+*^), **p_adj_ = 0.0012 (*Fgf21*^*hep−/−*^); *Pck1*, ****p_adj_ < 0.0001). (**F**) Plasma levels of carnitine, short chain acylcarnitines (SCAC, C2–C5), medium chain acylcarnitines (MCAC, C6–C12), and long-chain acylcarnitines (LCAC, C14–C18) (*n* = 6 mice per group, biological replicates, two-way ANOVA followed by Šídák’s multiple comparisons test α = 0.05, Fed vs Fasted in *Fgf21*^*hep+/+*^ or *Fgf21*^*hep−/−*^, ****p_adj_ < 0.0001). (**G**) Plasma level of β-hydroxybutyrate (*n* = 8–10 mice per group, biological replicates, two-way ANOVA followed by Šídák’s multiple comparisons test α = 0.05, Fed vs Fasted in *Fgf21*^*hep+/+*^ or *Fgf21*^*hep−/−*^, ****p_adj_ < 0.0001). (**H**) mRNA relative expression of *Cyp4a10*, *Cyp4a14*, *Ehhadh*, *Acot2*, and* Hmgcs2* in liver samples measured by qRT-PCR (*n* = 8–10 mice per group, biological replicates, two-way ANOVA followed by Šídák’s multiple comparisons test α = 0.05; Fed vs Fasted in *Fgf21*^*hep+/+*^ or *Fgf21*^*hep−/−*^, *Cyp4a10*, *Cyp4a14*, *Ehhadh*, *Acot2*, and *Hmgcs2*, ****p_adj_ < 0.0001; *Acot2*, **p_adj_ = 0.002). (**I**) mRNA relative expression of *Pparα*, *Vnn1*, *Fgf21*, *Txnip*, and *Pdk4* in liver samples measured by qRT-PCR (*n* = 8–10 mice per group, biological replicates, two-way ANOVA followed by Šídák’s multiple comparisons test α = 0.05; Fed vs Fasted in *Fgf21*^*hep+/+*^ or *Fgf21*^*hep-/-*^, *Pparα*, **p_adj_ = 0.0043 (*Fgf21*^*hep+/+*^), **p_adj_ = 0.0016 (*Fgf21*^*hep−/−*^); *Vnn1*, *Fgf21*, *Txnip*, and *Pdk4*, ****p_adj_ < 0.0001; *Pdk4*, ***p_adj_ = 0.0001; Fasted *Fgf21*^*hep+/+*^ *vs* Fasted *Fgf21*^*hep−/−*^, *Fgf21*, ####*p*_adj_ < 0.0001). Data information: All data were presented as mean ± SEM; * shows a fasting effect; # shows a genotype effect. Source data are available online for this figure.

Fasting induces a major shift in hepatic metabolic homeostasis (Fougerat et al, 2024; Ruppert and Kersten, 2024). To investigate the role of endogenous hepatocyte FGF21 in fasting-induced changes in hepatic homeostasis, *Fgf21*^*hep+/+*^ and *Fgf21*^*hep−/−*^ mice were either fed *ad libitum* or fasted for 20 h. Liver weight was significantly lower in fasted mice compared to fed animals, but did not differ between the genotypes (Fig. 2A). Hepatic triglyceride content and plasma alanine aminotransferase levels were also not affected by *Fgf21* deletion, both in fed and fasted mice (Fig. 2B,C). Consistent with these findings, hematoxylin and eosin staining of liver tissue did not reveal any difference between *Fgf21*^*hep+/+*^ and *Fgf21*^*hep−/−*^ mice (Fig. 2D). Similarly, the deletion of *Fgf21* in hepatocytes did not affect liver weight and circulating alanine aminotransferase in fasted females (Appendix Fig. S1E,F) nor in male mice fed a ketogenic diet (Appendix Fig. S2E,F).

**Figure EV1:**
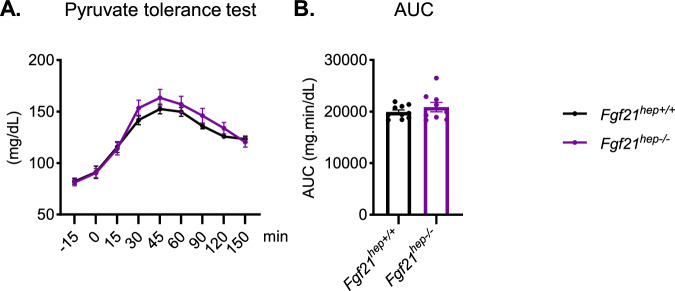
Hepatocyte-specific deletion of *Fgf21* does not affect glucose production during the pyruvate tolerance test. Related to Fig. 2. (**A**) Glycemia of pyruvate tolerance test (PTT) (*n* = 9–10 mice per group, biological replicates, Student’s *t*-test). (**B**) Area under the curve (AUC) for each mouse representing PTT results (*n* = 9–10 mice per group, biological replicates, Student’s *t*-test). Data information: All data were presented as mean ± SEM.

During fasting, the liver ensures the production of glucose and ketone bodies to supply peripheral tissues. After glycogen stores are depleted, glucose production depends on gluconeogenesis (Fougerat et al, 2024). We found that hepatic expression of key enzymes involved in gluconeogenesis, *G6pc* and *Pck1*, was increased in response to fasting in both *Fgf21*^*hep+/+*^ and *Fgf21*^*hep−/−*^ mice (Fig. 2E). These findings were further supported by a similar glucose production in the two genotypes, as assessed by the pyruvate tolerance test indicating that hepatocyte *Fgf21* deletion did not affect fasting-induced hepatic gluconeogenesis (Fig. EV1).

If fasting persists, gluconeogenesis decreases to prevent muscle protein breakdown while fatty acids derived from adipose tissue lipolysis are utilized to produce ketone bodies in the liver. Once in the liver, fatty acids are converted into acylcarnitines which, in turn, are beta-oxidized in mitochondria (Fougerat et al, 2024). In line with this, we found that fasting decreased the plasma level of free carnitine and increased short-, medium-, and long-chain acylcarnitine levels with no significant difference between genotypes (Fig. 2F). The increase in plasma ketone bodies and hepatic expression of genes involved in the regulation of fatty acid catabolism (β-oxidation and ketogenesis) was also similar in *Fgf21*^*hep+/+*^ and *Fgf21*^*hep−/−*^ mice in response to fasting (Fig. 2G,H). The induction of fatty acid oxidation and ketogenesis during fasting is mainly orchestrated transcriptionally by the nuclear receptor PPARα. Given that hepatic PPARα activity is regulated by adipose tissue lipolysis (Fougerat et al, 2022) and that FGF21 has been reported to regulate lipolysis (Inagaki et al, 2007; Hotta et al, 2009; Chen et al, 2011), FGF21 may exert a negative feedback or stimulate PPARα activity in hepatocytes during fasting. Except that of *Fgf21*, the expression of well-known PPARα target genes during fasting was unchanged in the absence of FGF21 in hepatocytes (Régnier et al, 2018) (Fig. 2I). Expression of these genes was also not impacted by *Fgf21* deletion in fasted females (Appendix Fig. S1G,H) and in males fed a ketogenic diet (Appendix Fig. S2G,H). Similar profiles were observed for genes involved in hepatic autophagy, a conserved catabolic process activated in the liver to recycle intracellular substrates and remove dysfunctional proteins during fasting (Appendix Fig. S3).

Together, these data indicate that hepatocyte FGF21 does not contribute to fasting-induced key hepatic metabolic pathways such as gluconeogenesis, fatty acid β-oxidation and ketogenesis. Additionally, our results do not support a role for hepatocyte FGF21 in the PPARα-dependent responses in the liver.

### Hepatocyte FGF21 deletion does not influence liver gene expression and metabolites in response to fasting

**Figure 3:**
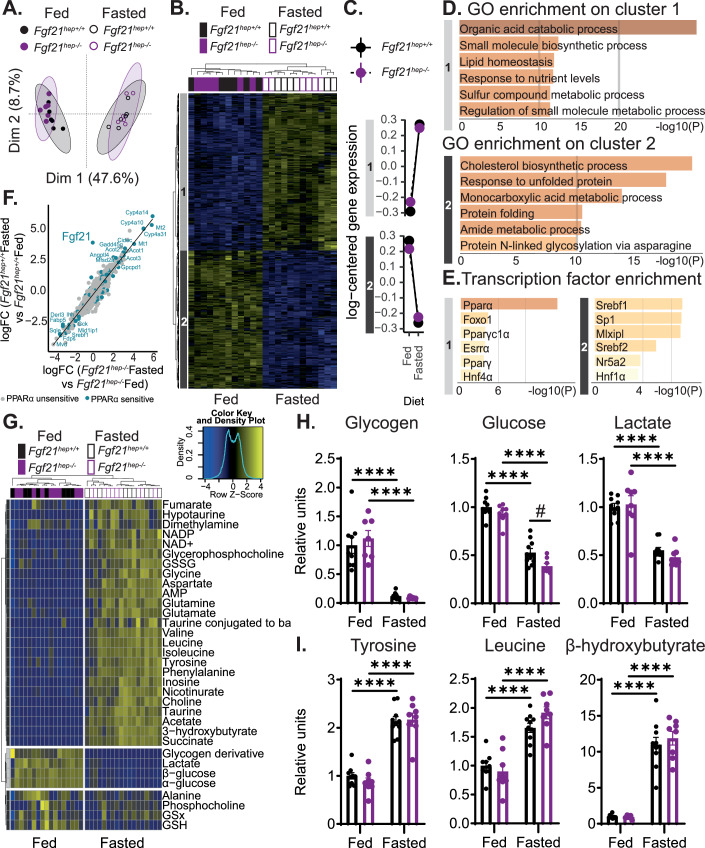
Hepatocyte FGF21 deletion does not influence liver gene expression and metabolites in response to fasting. *Fgf21* liver floxed (*Fgf21*^*hep+/+*^) or *Fgf21* liver knockout (*Fgf21*^*hep−/−*^) mice were fed *ad libitum* or fasted for 20 h. (**A**) Principal component analysis (PCA) score plots of the whole-liver transcriptome dataset. Each point represents a single mouse (*n* = 6 mice per group, biological replicates). (**B**–**E**) Microarray experiment was performed with liver samples. Hierarchical clustering shows the definition of two clusters, each column is a mouse, and each row represents one gene (*n* = 6 mice per group, biological replicates, FC >1.5; *p*_adj_ < 0.05). Hierarchical clustering analysis was performed according to similarities in their signal profiles, based on pairwise correlation. Clusters were generated using Ward’s minimum variance method) (**B**). Representation of the mean cluster profiles in each cluster (**C**). Gene Ontology (GO) categories enriched among the two clusters of genes (**D**) and enrichment of transcription factors (**E**) (functional enrichment analyses were performed using Metascape, as previously described (Zhou et al, 2019), integrating ontology (GO) and curated transcriptional regulatory (TRRUST) datasets, respectively. Statistical significance was assessed using the hypergeometric test, and *p* values were adjusted using the Benjamini–Hochberg procedure to control the false discovery rate. (**F**) Representation of fasting hepatic PPARα-dependent genes on the correlation of fasting-dependent genes in *Fgf21* liver floxed (*Fgf21*^*hep+/+*^) mice vs. fasting-dependent genes in *Fgf21* liver knockout (*Fgf21*^*hep−/−*^) mice. (**G**) Heatmap of liver metabolomic dataset. Hierarchical clustering shows the definition of three clusters, each column is a mouse, and each row represents one metabolite (*n* = 8–10 mice per group, biological replicates). Hierarchical clustering analysis was performed according to similarities in their signal profiles, based on pairwise correlation. Clusters were generated using Ward’s minimum variance method. (**H**, **I**) Area under the curve of the ^1^H-NMR spectra for representative metabolites that are significantly repressed (*n* = 8–10 mice per group, biological replicates, two-way ANOVA followed by Šídák’s multiple comparisons test α = 0.05; Fed vs Fasted in *Fgf21*^*hep+/+*^ or *Fgf21*^*hep−/−*^, glycogen, glucose, lactate, ****p_adj_ < 0.0001; Fasted *Fgf21*^*hep+/+*^ vs Fasted *Fgf21*^*hep−/−*^, glucose, #*p*_adj_ = 0.0154) (**H**) and induced (*n* = 8–10 mice per group, biological replicates, two-way ANOVA followed by Šídák’s multiple comparisons test α = 0.05; Fed vs Fasted in *Fgf21*^*hep+/+*^ or *Fgf21*^*hep−/−*^, tyrosine, leucine, β-hydroxybutyrate, ****p_adj_ < 0.0001) (**I**) by fasting. Data information: All data were presented as mean ± SEM; * shows a fasting effect; # shows a genotype effect. Source data are available online for this figure.

**Figure EV2:**
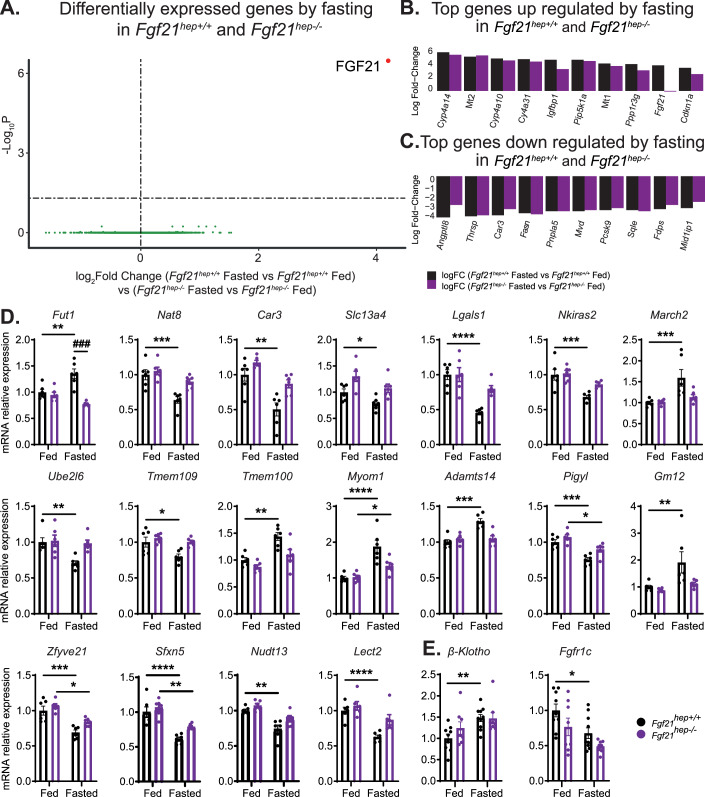
Hepatocyte-specific deletion of *Fgf21* affects the expression of only a few genes in the liver during fasting. Related to Fig. 3. *Fgf21* liver floxed (*Fgf21*^*hep+/+*^) or *Fgf21* liver knockout (*Fgf21*^*hep−/−*^) mice were fed *ad libitum* or fasted for 20 h. (**A**) Volcano plot representing the regulated genes in (*Fgf21*^*hep+/+*^ Fasted vs* Fgf21*^*hep+/+*^ Fed) vs (*Fgf21*^*hep−/−*^ Fasted *vs Fgf21*^*hep−/−*^ Fed) mice in liver samples. Green dots correspond to genes that are non-significant, and the red dot represents significant genes (*n* = 6 mice per group, biological replicates, log_2_FC >1: vertical line; −log_10_(*p*_adj_) >0.05: horizontal line). (**B**,** C**) LogFC of top genes up- (**B**) and down-regulated (**C**) by fasting from *Fgf21* liver floxed (*Fgf21*^*hep+/+*^) or *Fgf21* liver knockout (*Fgf21*^*hep−/−*^) mice (*n* = 6 mice per group, biological replicates). (**D**) mRNA relative expression of liver *Fut1*, *Nat8*, *Car3*, *Slc13a4*, *Lgals1*, *Nkiras2*, *March2*, *Ube2l6*, *Tmem109*, *Tmem100*, *Myom1*, *Adamts14*, *Pigyl*, *Gm12*, *Zfyve21*, *Sfxn5*, *Nudt13*, and *Lect2*, derived from microarray results (*n* = 6 mice per group, biological replicates, Pearson correlation analysis to identify genes whose expression levels are correlated with that of *Fgf21*: correlation coefficient threshold >0.7 and for each represented gene: limma package, with linear models fitted (lmFit), Fed vs Fasted in *Fgf21*^*hep+/+*^ or *Fgf21*^*hep−/−*^, *Fut1*, **p_adj_ = 0.003; *Nat8*, ***p_adj_ = 0.0002; *Car3*, **p_adj_ = 0.0019; *Slc13a4*, *p_adj_ = 0.048; *Lgals1*, ****p_adj_ < 0.0001; *Nkiras2*, ***p_adj_ = 0.00018; *March2*, ***p_adj_ = 0.0006; *Ube2l6*, **p_adj_ = 0.0016; *Tmem109*, *p_adj_ = 0.0123; *Tmem100*, **p_adj_ = 0.002; *Myom1*, ****p_adj_ < 0.0001 (*Fgf21*^*hep+/+*^), *p_adj_ = 0.033 (*Fgf21*^*hep−/−*^); *Adamts14*, ***p_adj_ = 0.0005; *Pigyl*, ***p_adj_ = 0.0004 (*Fgf21*^*hep+/+*^), *p_adj_ = 0.031 (*Fgf21*^*hep−/−-*^); *Gm12*, **p_adj_ = 0.0016; *Zfyve21*, ***p_adj_ = 0.00013 (*Fgf21*^*hep+/+*^), *p_adj_ = 0.0109 (*Fgf21*^*hep−/−*^); *Sfxn5*, ****p_adj_ < 0.0001 (*Fgf21*^*hep+/+*^), **p_adj_ = 0.0031 (*Fgf21*^*hep−/−*^); *Nudt13*, **p_adj_ = 0.0012; *Lect2*, ****p_adj_ < 0.0001); Fasted *Fgf21*^*hep+/+*^ vs Fasted *Fgf21*^*hep−/*^^−^, *Fut1*, ###*p*_adj_ = 0.0009. (**E**) mRNA relative expression of *β-Klotho and Fgfr1c* in liver samples measured by qRT-PCR (*n* = 8–10 mice per group, biological replicates, two-way ANOVA followed by Šídák’s multiple comparisons test α = 0.05; Fed vs Fasted in *Fgf21*^*hep+/+*^, *β-Klotho*, **p_adj_ = 0.008; *Fgfr1c*, *p_adj_ = 0.02). Data information: All data were presented as mean ± SEM; * shows a fasting effect; # shows a genotype effect.

Fasting induces a major change in liver gene expression and metabolome (Goldstein and Hager, 2015; Ruppert and Kersten, 2024). To further investigate the contribution of FGF21 on liver gene expression, we performed gene expression profiling on livers of fed and fasted *Fgf21*^*hep+/+*^ and *Fgf21*^*hep−/−*^ mice. Principal component analysis (PCA) showed that differences in gene expression were mostly observed between fed and fasted mice and indicated minimal effects of *Fgf21* deletion on the hepatic transcriptome (Fig. 3A). Volcano plot comparing differentially expressed genes (DEGs) between fed and fasted conditions revealed that *Fgf21* was the only gene significantly upregulated in response to fasting in *Fgf21*^*hep+/+*^ compared to *Fgf21*^*hep−/−*^ mice (Fig. EV2A). Hierarchical clustering on the DEGs (fold-change (FC) >1.5; *p*_adj_ < 0.05) confirmed the marked discrimination between fed and fasted mice, and identified two clusters showing specific gene expression in response to fasting, which were not dependent on *Fgf21* expression in hepatocytes (Fig. 3B). In the first cluster, 1467 genes were upregulated in response to fasting (Fig. 3C). Gene Ontology (GO) analysis revealed that these genes were most significantly associated with catabolic metabolic processes, mostly PPARα targets, as identified through transcription factor enrichment (Fig. 3D,E). Consistent with these observations, the most upregulated genes by fasting in *Fgf21*^*hep+/+*^ mice were known PPARα target genes. These genes were also upregulated in *Fgf21*^*hep−/−*^ mice, except for the deleted *Fgf21* (Fig. EV2B). The 1267 genes in cluster 2 exhibited lower mRNA expression in fasted mice compared to fed mice (Fig. 3B,C). This cluster was most significantly associated with ‘cholesterol biosynthetic process’ and enriched in SREBF1, SP1, and MLXIP target genes (Fig. 3D,E). The most significantly affected genes in cluster 2 included genes involved in lipogenesis and cholesterol metabolism, such as *Fasn*, *Pnpla5*, *Pcsk9*, and *Sqle* (Fig. EV2C). Next, we plotted the FC values for *Fgf21*^*hep−/−*^ mice in response to fasting (x-axis) against those for *Fgf21*^*hep+/+*^ mice under the same conditions (y-axis). A 45-degree trend line was observed, confirming that fasting exerts similar effects on liver gene expression in *Fgf21*^*hep+/+*^ and *Fgf21*^*hep−/−*^ mice (Fig. 3F). Consistent with our previous findings (Fig. 2H,I), fasting-induced PPARα-sensitive genes (Régnier et al, 2018; Data ref: Montagner et al, 2016) clustered closely along the trend line, with the exception of *Fgf21*. Finally, we performed a Pearson correlation analysis to identify genes whose expression levels are correlated with that of *Fgf21*, using a correlation coefficient threshold of >0.7. The expression profiles of these genes were not significantly different between fasted *Fgf21*^*hep+/+*^ and fasted *Fgf21*^*hep−/−*^ mice, with the exception of *Fut1* (Fig. EV2D).

To further explore the potential impact of *Fgf21* deficiency, we next performed hepatic metabolomic profiling of aqueous metabolites using ^1^H-NMR. This analysis confirmed the presence of marked fasting-dependent hepatic metabolites, which remained independent of *Fgf21* expression in hepatocytes (Fig. 3G). Among the metabolites reduced during fasting, we found glycogen, glucose, and lactate, which are utilized by the liver as energy substrates (Fig. 3H). Conversely, the concentrations of several amino acids were increased in fasted mice, likely deriving from protein degradation. Hepatic β-hydroxybutyrate, the most abundant ketone body, was also increased by fasting in both genotypes (Fig. 3I).

Collectively, these data indicate that the fasting-induced changes in hepatic gene expression and metabolome are not dependent on hepatocyte FGF21. Given the significant increase in circulating FGF21 levels during fasting in *Fgf21*^*hep+/+*^ mice, these results were somewhat unexpected. We confirmed that hepatic expression of the FGF21 receptor complex, including *Fgfr1c* and *β-Klotho*, was similar in *Fgf21*^*hep+/+*^ and *Fgf21*^*hep−/−*^ mice in both fed and fasted mice (Fig. EV2E). In addition, hepatic expression of several other hepatokines remained unaltered, ruling out compensatory upregulation mechanisms in the absence of FGF21 (Appendix Fig. S4).

### Hepatocyte FGF21 deletion does not influence adipose tissue responses to fasting

Having established that autocrine FGF21 signaling is not essential for fasting-induced responses in the liver, we next asked whether hepatic FGF21 influences adipose tissue function. While it is clear that FGF21 signaling in adipose tissue has insulin-sensitizing (Arner et al, 2008; Li et al, 2009) and lipolysis inhibitory effects in obese mice (Ding et al, 2012; BonDurant et al, 2017), the contribution of FGF21 to adipose tissue lipolysis during fasting remains debated (Hotta et al, 2009; Chen et al, 2011; Potthoff et al, 2009; Badman et al, 2009; Sostre-Colón et al, 2022). We therefore investigated the potential contribution of hepatocyte-derived FGF21 to the physiology of the adipose tissues.

**Figure EV3:**
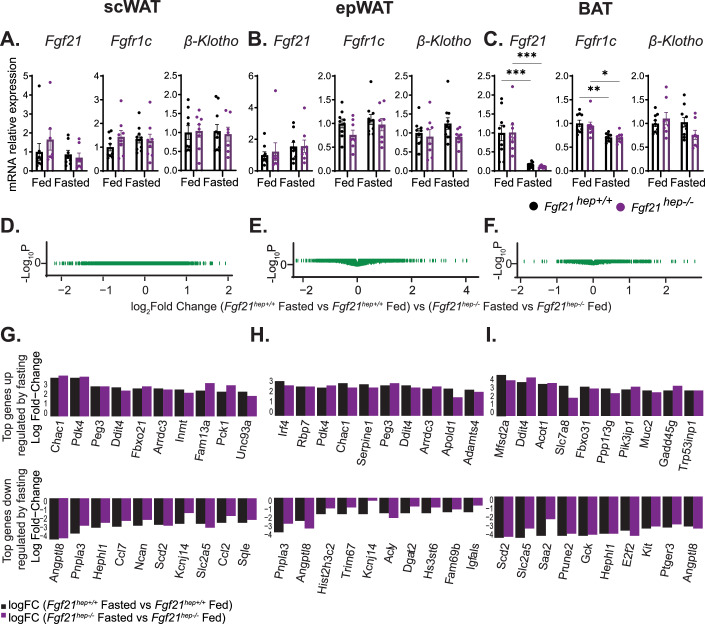
Hepatocyte-specific deletion of *Fgf21* does not affect fasting-induced adipose tissue gene expression. Related to Figs. 4 and 5. *Fgf21* liver floxed (*Fgf21*^*hep+/+*^) or *Fgf21* liver knockout (*Fgf21*^*hep−/−*^) mice were fed *ad libitum* or fasted for 20 h. (**A**) mRNA relative expression of *Fgf21*, *Fgfr1c*, and *β-Klotho* in subcutaneous white adipose tissue (scWAT) samples measured by qRT-PCR (*n* = 7–10 mice per group, biological replicates, two-way ANOVA followed by Šídák’s multiple comparisons test α = 0.05). (**B**) mRNA relative expression of *Fgf21*, *Fgfr1c*, and *β-Klotho* in epididymal white adipose tissue (epWAT) samples measured by qRT-PCR (*n* = 8–10 mice per group, biological replicates, two-way ANOVA followed by Šídák’s multiple comparisons test α = 0.05). (**C**) mRNA relative expression of *Fgf21*, *Fgfr1c*, and *β-Klotho* in brown adipose tissue (BAT) samples measured by qRT-PCR (*n* = 7–10 mice per group, biological replicates, two-way ANOVA followed by Šídák’s multiple comparisons test α = 0.05; Fed vs Fasted in *Fgf21*^*hep+/+*^ or *Fgf21*^*hep−/−*^, *Fgf21*, ***p_adj_ = 0.0003 (*Fgf21*^*hep+/+*^), ***p_adj_ = 0.0005 (*Fgf21*^*hep−/−*^); *Fgfr1c*, **p_adj_ = 0.0019, *p_adj_ = 0.014). (**D**–**F**) Volcano plot representing the regulated genes in (*Fgf21*^*hep+/+*^ Fasted *vs Fgf21*^*hep+/+*^ Fed) vs (*Fgf21*^*hep−/−*^ Fasted *vs Fgf21*^*hep−/−*^ Fed) mice in scWAT (**D**), epWAT (**E**), and BAT (**F**) samples. Green dots correspond to genes that are non-significant (*n* = 6 mice per group, biological replicates, Log_2_FC >1; under −log_10_(*p*_adj_) >0.05). (**G**–**I**) LogFC of top genes up- and down-regulated by fasting from *Fgf21* liver floxed (*Fgf21*^*hep+/+*^) or *Fgf21* liver knockout (*Fgf21*^*hep−/−*^) mice (*n* = 6 mice per group, biological replicates) in scWAT (**G**), epWAT (**H**), and BAT (**I**) samples. Data information: All data were presented as mean ± SEM; * shows a fasting effect.

**Figure 4:**
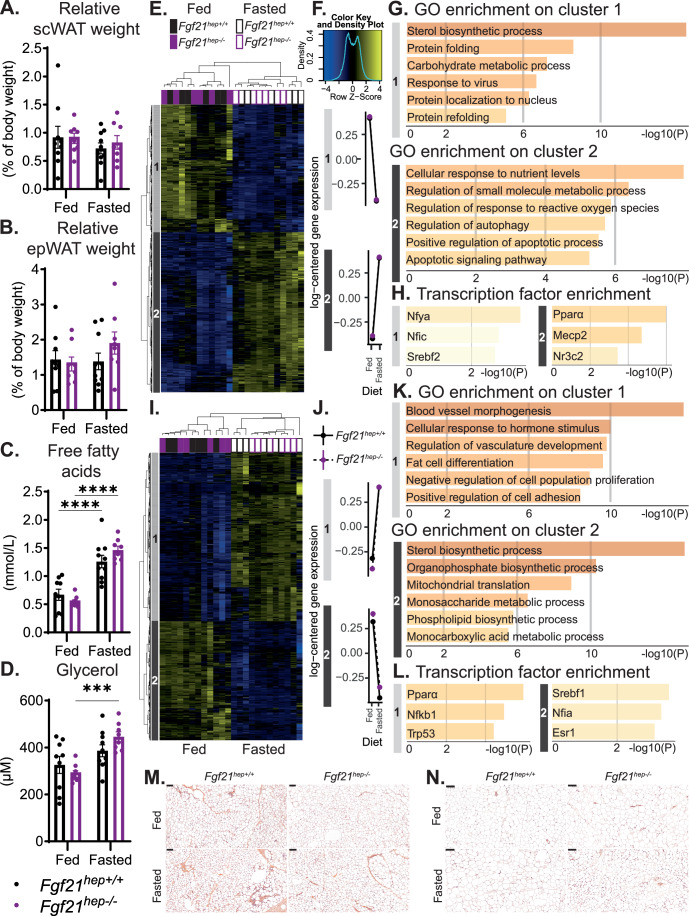
Hepatocyte FGF21 deletion does not influence white adipose tissue responses to fasting. *Fgf21* liver floxed (*Fgf21*^*hep+/+*)^ or *Fgf21* liver knockout (*Fgf21*^*hep−/−*^) mice were fed *ad libitum* or fasted for 20 h. (**A**) Relative subcutaneous white adipose tissue (scWAT) weight (*n* = 8–10 mice per group, biological replicates, two-way ANOVA followed by Šídák’s multiple comparisons test α = 0.05). (**B**) Relative epididymal white adipose tissue (epWAT) weight (*n* = 8–10 mice per group, biological replicates, two-way ANOVA followed by Šídák’s multiple comparisons test α = 0.05). (**C**) Plasma free fatty acids (*n* = 8–10 mice per group, biological replicates, two-way ANOVA followed by Šídák’s multiple comparisons test α = 0.05, Fed vs Fasted in *Fgf21*^*hep+/+*^ or *Fgf21*^*hep−/−*^, ****p_adj_ < 0.0001). (**D**) Plasma level of glycerol (*n* = 8–10 mice per group, biological replicates, two-way ANOVA followed by Šídák’s multiple comparisons test α = 0.05, Fed vs Fasted in *Fgf21*^*hep−/−*^, ***p_adj_ = 0.0006). (**E**–**H**) Microarray experiment performed with scWAT samples. Hierarchical clustering shows the definition of two clusters, each column is a mouse, and each row represents one gene (*n* = 6 mice per group, biological replicates, FC >1.5; *p*_adj_ <0.05). Hierarchical clustering analysis was performed according to similarities in their signal profiles, based on pairwise correlation. Clusters were generated using Ward’s minimum variance method) (**E**). Representation of the mean cluster profiles in each cluster (**F**). Gene Ontology (GO) categories enriched among the two clusters of genes (**G**) and enrichment of transcription factors (**H**) (functional enrichment analyses were performed using Metascape, as previously described (Zhou et al, 2019), integrating ontology (GO) and curated transcriptional regulatory (TRRUST) datasets, respectively. Statistical significance was assessed using the hypergeometric test, and *p* values were adjusted using the Benjamini–Hochberg procedure to control the false discovery rate. (**I**–**L**) Microarray experiment performed with epWAT samples. Hierarchical clustering shows the definition of two clusters, each column is a mouse, and each row represents one gene (*n* = 6 mice per group, biological replicates, FC >1.5; *p*_adj_ < 0.05). Hierarchical clustering analysis was performed according to similarities in their signal profiles, based on pairwise correlation. Clusters were generated using Ward’s minimum variance method (**I**). Representation of the mean cluster profiles in each cluster (**J**). Gene Ontology (GO) categories enriched among the two clusters of genes (**K**) and enrichment of transcription factors (**L**) (functional enrichment analyses were performed using Metascape, as previously described (Zhou et al, 2019), integrating ontology (GO) and curated transcriptional regulatory (TRRUST) datasets, respectively. Statistical significance was assessed using the hypergeometric test, and *p* values were adjusted using the Benjamini–Hochberg procedure to control the false discovery rate. (**M**,** N**) Representative pictures of H&E staining of scWAT (**M**) and epWAT (**N**) sections. Scale bars, 100 μm. Data information: All data were presented as mean ± SEM; * shows a fasting effect. Source data are available online for this figure.

We first measured the expression of *Fgf21* and its receptor complex in subcutaneous (sc) and epididymal (ep) white adipose tissues (WAT), which was not affected by hepatocyte *Fgf21* deletion in both tissues (Fig. EV3A,B). The relative weight of WATs was not significantly different between groups (Fig. 4A,B). Circulating free fatty acids and glycerol were increased in fasted mice compared to fed mice but not different between the two genotypes, indicating that the lack of FGF21 in hepatocytes does not influence WAT lipolysis during fasting (Fig. 4C,D). Similar results were observed in fasted females (Appendix Fig. S1I–L) as well as in males fed a ketogenic diet (Appendix Fig. S2I–L).

To further examine the potential effect of hepatocyte *Fgf21* deletion on white adipose tissues, we performed microarray analysis on scWAT and epWAT gene expression. In both tissues, hierarchical clustering using the DEGs (FC >1.5; *p*_adj_ < 0.05) identified two main gene clusters and revealed a marked discrimination only between fed and fasted animals, independent of *Fgf21* expression in hepatocytes (Fig. 4E,I). Volcano plots comparing DEGs between fasted and fed conditions in both genotypes further supported this conclusion. Notably, no gene met the significant p_value threshold (Fig. EV3D,E). The most up- and down-regulated genes in response to fasting in scWAT and epWAT are shown in Fig. EV3G,H. In scWAT, cluster 1 comprised genes that are downregulated following fasting. These genes are mainly associated with “sterol biosynthetic process”. In contrast, genes in cluster 2 exhibited a higher expression in fasted mice and were involved in “cellular response to nutrient levels” (Fig. 4F,G). In epWAT, genes in cluster 1 were upregulated by fasting and associated with “blood vessel morphogenesis”, “cellular response to hormone stimulus” and “regulation of vasculature development”, indicating transcriptional changes related to tissue remodeling and cellular plasticity. As for scWAT, genes with lower expression upon fasting (cluster 2) were mainly involved in “sterol biosynthetic process” (Fig. 4J,K). In both WAT depots, genes induced by fasting were identified mostly as PPARα target genes, while SREBP1 and SREBP2 targets are repressed by fasting (Fig. 4H,L). Histological analysis of WATs sections stained with hematoxylin and eosin (H&E) revealed no marked difference in adipocyte morphology (Fig. 4M,N).

**Figure 5:**
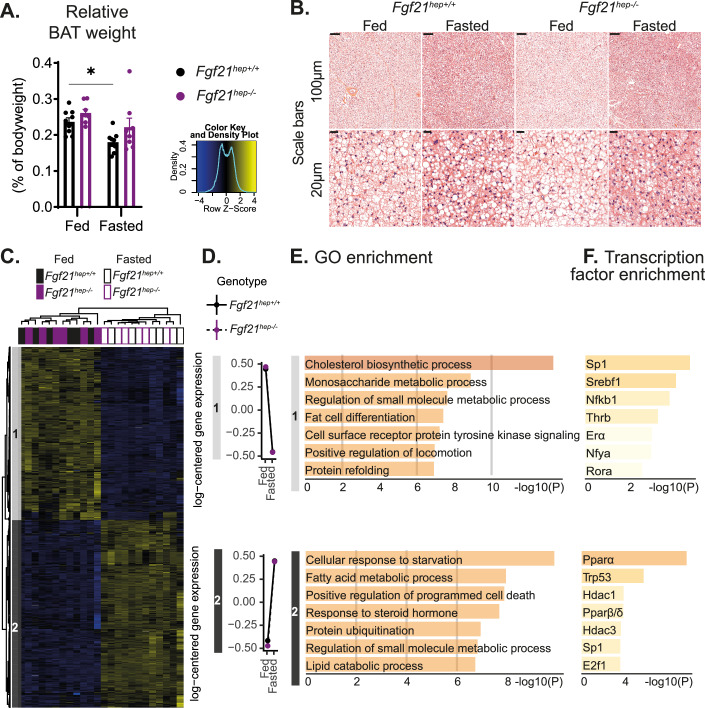
Hepatocyte-specific deletion of *Fgf21* does not affect fasting-induced brown adipose tissue responses. *Fgf21* liver floxed (*Fgf21*^*hep+/+*^) or *Fgf21* liver knockout (*Fgf21*^*hep−/−*^) mice were fed *ad libitum* or fasted for 20 h. (**A**) Relative (brown adipose tissue) BAT weight (*n* = 8–10 mice per group, biological replicates, two-way ANOVA followed by Šídák’s multiple comparisons test α = 0.05, Fed vs Fasted in *Fgf21*^*hep+/+*^, *p_adj_ = 0.0127). (**B**) Representative pictures of H&E staining of BAT sections. Scale bars, 100 and 20 µm. (**C**–**F**) Microarray experiment performed with BAT samples. Hierarchical clustering shows the definition of two clusters, each column is a mouse, and each row represents one gene (*n* = 6 mice per group, biological replicates, FC >1.5; *p*_adj_ < 0.05). Hierarchical clustering analysis was performed according to similarities in their signal profiles, based on pairwise correlation. Clusters were generated using Ward’s minimum variance method) (**C**). Representation of the mean cluster profiles in each cluster (**D**). Gene Ontology (GO) categories enriched among the two clusters of genes (**E**) and enrichment of transcription factors (**F**) (functional enrichment analyses were performed using Metascape, as previously described (Zhou et al, 2019), integrating ontology (GO) and curated transcriptional regulatory (TRRUST) datasets, respectively. Statistical significance was assessed using the hypergeometric test, and *p* values were adjusted using the Benjamini–Hochberg procedure to control the false discovery rate. Data information: All data were presented as mean ± SEM; * shows a fasting effect. Source data are available online for this figure.

In BAT, expression of *Fgf21* and its receptor complex was similarly unaffected by hepatocyte *Fgf21* deletion (Fig. EV3C), and BAT relative weight did not differ between genotypes (Fig. 5A). BAT sections stained with H&E also showed no apparent morphological changes in adipocytes between *Fgf21*^*hep+/+*^ and *Fgf21*^*hep−/−*^ mice (Fig. 5B). We assessed BAT gene expression using microarray. The gene clustering (FC >1.5; *p*_adj_ < 0.05) discriminated fed from fasted animals, independently of hepatocyte *Fgf21* expression (Figs. 5C,D and EV3F). Genes with lower expression in fasted BAT were involved in the “cholesterol biosynthetic process” and identified as SREBP1 targets, while genes upregulated by fasting were associated with “cellular response to starvation” and enriched for PPARα targets (Fig. 5E,F). The most up- and down-regulated genes in response to fasting in BAT are shown in Fig. EV3I.

Taken together, these results provide a detailed characterization of gene expression in three different adipose tissues in response to fasting (Ruppert and Kersten, 2025). Our findings indicate that these adipose tissue responses to fasting are largely independent of *Fgf21* expression in hepatocytes and circulating FGF21 levels.

### Hepatocyte FGF21 drives protein preference following fasting

Beyond its role in metabolic regulation, FGF21 has been implicated in the control of nutrient-driven behaviors, particularly in protein appetite and sweet preference (Von Holstein-Rathlou et al, 2016; Solon-Biet et al, 2023; Laeger et al, 2014; Hill et al, 2019; Nicolaisen et al, 2025; Kim et al, 2024). To explore this aspect, we examined whether the deletion of *Fgf21* in hepatocytes may affect systemic energy expenditure and macronutrient preference.

We first performed metabolic phenotyping *via* indirect calorimetry in fed, fasted and refed *Fgf21*^*hep+/+*^ and *Fgf21*^*hep−/−*^ mice. There was no difference in physical activity between genotypes under all nutritional conditions (Appendix Fig. S5A). In the fed state, oxygen consumption (VO_2_), carbon dioxide production (VCO₂), and total energy expenditure (EE) were slightly higher in *Fgf21*^*hep−/−*^ mice than in *Fgf21*^*hep+/+*^ mice at the beginning of the active dark period (Appendix Fig. S5B–D). However, the expiratory exchange ratio was similar in both genotypes (Appendix Fig. S5E). None of these parameters was strongly affected by *Fgf21* deletion in the fasted state (Appendix Fig. S5B–E). Most changes were observed during refeeding with *Fgf21*^*hep−/−*^ mice exhibiting higher VO_2_, VCO₂, and EE than *Fgf21*^*hep+/+*^ mice (Appendix Fig. S5B–D). As for fed mice, the respiratory exchange ratio was similar in the two genotypes during refeeding, indicating similar substrate utilization (Appendix Fig. S5E).

We then investigated the impact of hepatocyte *Fgf21* deletion during refeeding following a fasting period. Food choice after fasting represents a critical nutritional checkpoint during which animals must prioritize specific macronutrients to restore metabolic balance. Protein is unique among the macronutrients as it provides essential amino acids. A reduction in protein intake triggers adaptive physiological and behavioral changes in food intake and preference to prevent protein deficiency (Kim et al, 2024). FGF21 is robustly induced in the liver in response to protein restriction (Laeger et al, 2014; De Sousa-Coelho et al, 2012; Nicolaisen et al, 2025; Solon-Biet et al, 2023) and acts in the brain to enhance protein appetite while suppressing sugar preference (Laeger et al, 2014, 2016; Hill et al, 2019; Talukdar et al, 2016).

**Figure EV4:**
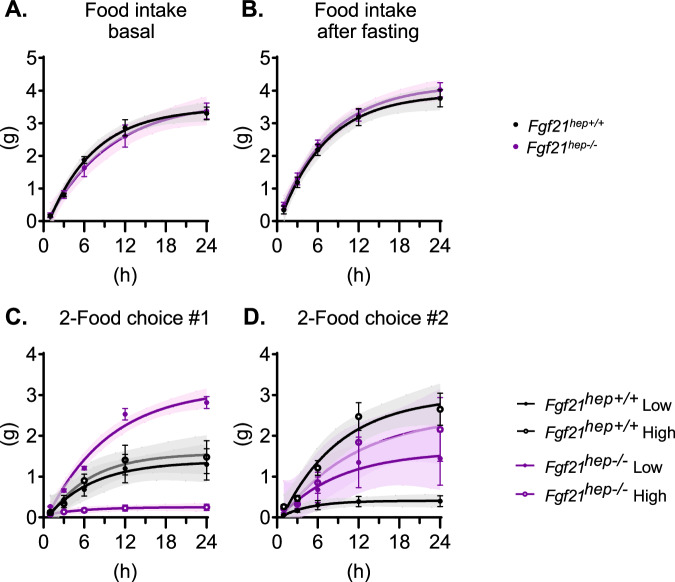
Hepatic FGF21 is required for protein preference after fasting from the first hours of feeding. Related to Fig. 6. (**A**,** B**) Food intake of singly housed mice was monitored over 24 h after 4, 6, 12, and 24 h in mice fed *ad libitum* with a standard diet (**A**) or refed *ad libitum* with a standard diet after 20 h of fasting (**B**) (*n* = 5–6 mice per group, nonlinear regression with an exponential plateau was fitted to the data, and 95% confidence bands were plotted). (**C**,** D**) Food intake of singly housed mice was monitored over 24 h after 4, 6, 12, and 24 h of refeeding of mice fed *ad libitum* with a low-protein diet (low-protein/high-carb diet: 6.5% protein and 80.4% carbohydrate) and a high-protein diet (high-protein/low-carb diet: 42.6% protein and 44.3% carbohydrate) (**C**) or *ad libitum* with a low-protein diet (low-protein/high-carb diet: 6.5% protein and 80.4% carbohydrate) and a high-protein diet (high-protein/high-carb diet: 17.8% protein, 70.4% carbohydrate) (**D**) (*n* = 5–6 mice per group, nonlinear regression with an exponential plateau was fitted to the data, and 95% confidence bands were plotted). Data information: Only the regression curves are shown on the graphs, with data points displayed as mean ± SEM.

**Figure 6:**
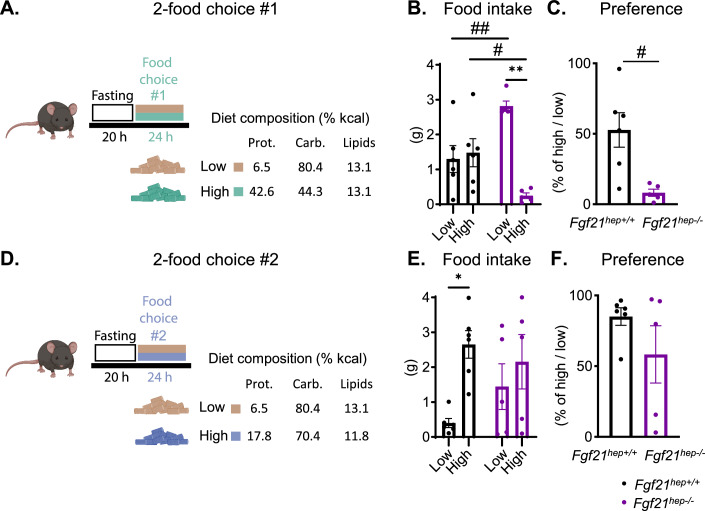
Hepatic FGF21 is required for protein preference after fasting. (**A**) *Fgf21* liver floxed (*Fgf21*^*hep+/+*^) or *Fgf21* liver knockout (*Fgf21*^*hep−/−*^) mice were fasted for 20 h and were exposed *ad libitum* to a low-protein diet (low-protein/high-carb diet: 6.5% protein and 80.4% carbohydrate) and a high-protein diet (high-protein/low-carb diet: 42.6% protein, 44.3% carbohydrate) for 24 h. (**B**) Food intake over 24 h after 20 h of fasting (*n* = 5–6 mice per group, biological replicates, two-way RM ANOVA followed by Fisher’s LSD, Low-protein diet vs high-protein diet in *Fgf21*^*hep−/−*^, **p = 0.003; *Fgf21*^*hep+/+*^ vs* Fgf21*^*hep−/−*^, ##*p* = 0.0032 (low-protein diet), #*p* = 0.013 (high-protein diet)). (**C**) Food preference for the high-protein diet over 24 h after 20 h of fasting (*n* = 5–6 mice per group, biological replicates, *t*-test with Welch’s correction test, *Fgf21*^*hep+/+*^ *vs Fgf21*^*hep−/−*^, #*p* = 0.0138, *p* value of *F*-test for variances comparison = 0.0068). (**D**) *Fgf21* liver floxed (*Fgf21*^*hep+/+*^) or *Fgf21* liver knockout (*Fgf21*^*hep−/−*^) mice were fasted for 20 h and were exposed *ad libitum* to a low-protein diet (low-protein/high-carb diet: 6.5% protein and 80.4% carbohydrate) and a high-protein diet (high-protein/high-carb diet: 17.8% protein and 70.4% carbohydrate) for 24 h. (**E**) Food intake over 24 h after 20 h of fasting (*n* = 5–6 mice per group, biological replicates, two-way RM ANOVA followed by Fisher’s LSD, low-protein diet vs high-protein diet in *Fgf21*^*hep+/+*^, *p = 0.0372). (**F**) Food preference for the high-protein diet over 24 h after 20 h of fasting (*n* = 5–6 mice per group, biological replicates, *t*-test with Welch’s correction test, *Fgf21*^*hep+/+*^ vs* Fgf21*^*hep−/−*^, *p* value of *F*-test for variances comparison = 0.0333). Data information: All data were presented as mean ± SEM; * shows a diet effect; # shows a genotype effect. Mice were singly housed. The percentage of preference was calculated for each mouse by dividing the intake of the low-protein diet or high-protein diet by the total food intake (low-protein + high-protein diets). Source data are available online for this figure.

To test whether hepatic FGF21 contributes to nutrient selection following fasting, we performed a two-choice preference test after fasting in *Fgf21*^*hep+/+*^ and in *Fgf21*^*hep−/−*^ mice. During the baseline acclimation phase, no significant difference in 24-h food intake was observed between genotypes (Fig. EV4A). Food intake during 24-h refeeding after 20 h of fasting was also comparable between genotypes (Fig. EV4B). In a first two-food choice test, mice were fasted for 20 h and then exposed simultaneously *ad libitum* to a low-protein diet (6.5% protein, 80.4% carbohydrate, 3.8 kcal/g) and to a high-protein diet (42.6% protein, 44.3% carbohydrate, 3.8 kcal/g) for 24 h (Appendix Table S1 and Fig. 6A). *Fgf21*^*hep+/+*^ mice displayed a balanced consumption of both diets. In contrast, *Fgf21*^*hep−/−*^ mice consumed significantly less of the high-protein diet than the low-protein diet (Fig. 6B). Preference analysis confirmed that *Fgf21*^*hep+/+*^ mice displayed a relative balance between diets (52.72% ± 8.97 preference high/low), while *Fgf21*^*hep−/−*^ mice showed an impaired ability to shift toward the high-protein diet (8.18% ± 4.58 preference high/low) (Fig. 6C), from the first hours of refeeding (Fig. EV4C).

In the first assay, the high-protein diet also had a lower carbohydrate content compared to the low-protein diet (44.3 vs 80.4%). To distinguish between protein and carbohydrate effects, we performed a second two-choice test in which both high- and low-protein diets (17.8 and 6.5% protein, respectively) were adjusted with a comparable amount of carbohydrates (70.4 and 80.4% carbohydrate, respectively) (Appendix Table S1 and Fig. 6D). In this second assay, *Fgf21*^*hep+/+*^ mice displayed a marked preference for the high-protein diet straightaway (85.10% ± 6.19 preference high/low), whereas *Fgf21*^*hep−/−*^ mice consumed the same amount of both diets (58.35% ± 17.53 preference high/low) (Figs. 6E,F and EV4D). Importantly, total food intake was not different between genotypes (Fig. 6B,E), indicating that FGF21 influences nutrient selection toward proteins rather than global caloric intake.

Taken together, these data demonstrate that hepatocyte FGF21 is required for protein preference following fasting.

## Discussion

FGF21 is widely regarded as an endocrine regulator of fasting-induced metabolic adaptations. This view stems largely from the early discovery that FGF21 is a liver-derived hormone robustly induced during fasting (Inagaki et al, 2007; Badman et al, 2007; Gälman et al, 2008). FGF21 has also been implicated in response to diverse metabolic stress such as cold exposure (Fisher et al, 2012; Ameka et al, 2019), ketogenic diet consumption (Badman et al, 2007; Watanabe et al, 2020), mitochondrial dysfunction (Croon et al, 2022), integrated stress response (Xu et al, 2018), high carbohydrate consumption (Dushay et al, 2015; Von Holstein-Rathlou et al, 2016; Iroz et al, 2017), alcohol consumption (Zhao et al, 2015; Talukdar et al, 2016), obesity and steatotic liver diseases (Dushay et al, 2010). Given the broad metabolic effects of recombinant FGF21 or FGF21 mimetics (Kliewer and Mangelsdorf, 2019), they are being actively explored as a therapeutic strategy for metabolic diseases (Rose et al, 2025; Noureddin et al, 2025; Loomba et al, 2023; Harrison et al, 2024). Here, we revisited the metabolic role of FGF21 in fasting. Using mice with hepatocyte-specific deletion of *Fgf21*, we show that hepatocyte-derived FGF21 is dispensable for core metabolic responses to fasting, including glycemic control, adipose tissue lipolysis, ketogenesis, and fasting-induced gene expression changes. Instead, our data reveal a more selective role for hepatocyte-derived FGF21 in shaping post-fasting nutrient preference, specifically by promoting protein intake during refeeding.

Consistent with previous reports, we confirm that fasting-induced circulating FGF21 originates almost exclusively from hepatocytes (Montagner et al, 2016; Markan et al, 2014) and is dependent on PPARα activity (Inagaki et al, 2007; Badman et al, 2007; Lundåsen et al, 2007; Montagner et al, 2016; Régnier et al, 2018; Fougerat et al, 2022). However, hepatocyte-specific *Fgf21* deletion did not impair fasting-induced ketone body production, glucose lowering, or body weight loss. This contrasts with studies reporting defective ketogenesis and impaired gluconeogenesis in global *Fgf21* knockout mice (Potthoff et al, 2009; Liang et al, 2014), but aligns with others demonstrating preserved ketone and glucose production in both loss- and gain-of-function models (Badman et al, 2009; Hotta et al, 2009; Inagaki et al, 2007; Antonellis et al, 2016). Our findings support the notion that the metabolic phenotypes observed in whole-body *Fgf21*-deficient mice may reflect contributions from extrahepatic FGF21 signaling, rather than a direct autocrine effect of hepatocyte-derived FGF21. At the hepatic level, genome-wide gene expression profiling and metabolomic analysis revealed that fasting-induced metabolic remodeling was preserved in the absence of hepatocyte-derived FGF21. The PPARα-dependent induction of hepatic genes critical for fatty acid oxidation and ketogenesis pathways were unchanged. These near-identical fasting signatures observed in control and *Fgf21*^*hep−/−*^ mice argue against a role for FGF21 as either a feed-forward or feedback regulator of hepatic PPARα activity or of any other fasting-sensitive pathway. Together, these results position FGF21 as a marker, rather than a mediator, of the hepatic fasting response. Previous studies have reported that FGF21 acts on adipose tissue metabolism (Hotta et al, 2009; Chen et al, 2011; Potthoff et al, 2009; Badman et al, 2009) and its hepatocyte-specific deletion alters liver-to-adipose tissue communication in mouse models of liver-specific insulin resistance (Emanuelli et al, 2014; Sostre-Colón et al, 2021) and in diet-induced obesity (Vernia et al, 2014, 2016). Therefore, we also examined whether hepatocyte-derived FGF21 modulates fasting-induced adipose tissue responses. Despite robust transcriptional remodeling of white and brown adipose tissues during fasting, these adaptations were fully preserved in the absence of hepatocyte FGF21. Circulating free fatty acid and glycerol levels were similarly increased in fasted control and mutant mice, indicating intact lipolytic responses. These data are in accordance with recent work showing that acute deletion of hepatic FGF21 does not affect fasting-induced adipose tissue lipolysis in mice (Sostre-Colón et al, 2022). Together, these findings suggest that adipose tissue adaptations to fasting are primarily governed by changes in classical hormonal cues, such as insulin, glucagon, or glucocorticoids, rather than by endocrine FGF21 signaling (Kersten, 2023; Ruppert and Kersten, 2025; Goldberg and Goldstein, 2026).

In contrast to its dispensability for fasting metabolism, hepatocyte-derived FGF21 emerged as a critical regulator of nutrient choice during refeeding. Following fasting, mice lacking hepatocyte FGF21 failed to appropriately shift food preference toward protein-rich diets, despite normal total caloric intake. This phenotype was evident even when carbohydrate content was matched between diets, indicating a specific defect in protein preference rather than altered energy sensing. These data extend previous studies identifying FGF21 as a hormone linking protein restriction to adaptive feeding behavior and establish hepatocytes as a key physiological source of this signal during fasting–refeeding transitions (Laeger et al, 2014, 2016). Recent data implicating hepatic FGF21 in sex-specific adaptations to juvenile protein malnutrition further reinforce its role as an amino acid–sensitive endocrine coordinator (preprint: Joly et al, 2025). Moreover, dietary protein restriction increases circulating FGF21 levels in humans, reinforcing the notion that FGF21 serves as a conserved endocrine signal of protein deficiency across species (Nicolaisen et al, 2025). However, while FGF21 controls metabolic adaptations to protein restriction, including increased food intake and decreased body weight, our data show that FGF21 is not required for metabolic adaptations during fasting but becomes essential during the refeeding period to guide macronutrient selection toward proteins. Mechanistically, this finding supports a model in which liver-derived FGF21 functions predominantly as an endocrine signal acting on the central nervous system to coordinate post-fasting nutrient selection. FGF21 receptors are expressed in discrete brain regions involved in nutrient sensing and reward (Liang et al, 2014; Bookout et al, 2013; Jensen-Cody et al, 2020), and previous work has demonstrated that central FGF21 signaling is required for adaptive protein appetite (Hill et al, 2019; Khan et al, 2025). While the precise neuronal circuits engaged by fasting-induced FGF21 remain to be identified, our data strongly support the existence of a liver–brain axis that prioritizes protein acquisition after food deprivation, likely to restore essential amino acid balance.

Fasting engages multiple nutrient-sensing transcriptional pathways, among which ATF4 and PPARα play central but distinct roles in coordinating metabolic adaptation (Goldstein and Hager, 2015; Bideyan et al, 2021). ATF4 is primarily activated in response to amino acid insufficiency through the integrated stress response, promoting transcriptional programs that support cellular homeostasis and stress adaptation, including induction of *Fgf21* (De Sousa-Coelho et al, 2012; Laeger et al, 2014, 2016; Hill et al, 2022). In contrast, PPARα is a key regulator of lipid metabolism during fasting (Kersten et al, 1999; Montagner et al, 2016; Régnier et al, 2018; Fougerat et al, 2022), driving hepatic fatty acid oxidation and ketogenesis (Fougerat et al, 2024; Ruppert and Kersten, 2024). Emerging evidence suggests functional crosstalk between these pathways, whereby ATF4-dependent signals may modulate PPARα activity or transcriptional output, thereby integrating amino acid availability with lipid metabolic responses and the regulation of *Fgf21* expression in hepatocytes. Such coordination likely contributes to hepatic metabolic flexibility during fasting, although the molecular mechanisms linking ATF4, PPARα, and FGF21 remain incompletely understood.

In summary, our study challenges the prevailing view of FGF21 as a central regulator of fasting-induced metabolic adaptations in liver and adipose tissues. Beyond clarifying the role of FGF21, this work also provides a gene expression resource of metabolically active tissues mobilized during fasting, which may inform future investigations into systemic responses to nutrient deprivation. Together, our data support a model in which hepatocyte-derived FGF21 functions as an endocrine signal that is largely dispensable for fasting metabolism but becomes important during refeeding, where it contributes to the guidance of macronutrient selection toward protein. This behavioral role may represent a key biological function of FGF21, potentially coordinating nutrient-seeking behavior with metabolic state to facilitate recovery from essential nutrient deprivation (Solon-Biet et al, 2023). In addition, a growing body of evidence indicates that FGF21 is robustly induced in response to infectious diseases (Fan et al, 2025; Huen et al, 2021), conditions that are frequently associated with reduced food intake and sickness behavior (Huen et al, 2021; Wang et al, 2016). These observations raise the possibility that FGF21 may play an underappreciated metabolic role in pathological contexts characterized by sustained anorexia. Importantly, our study examined changes in gene expression and metabolic adaptations in response to a 20-h fast in mice, as longer fasting durations could not be explored for ethical reasons. Under these conditions, we did not detect a significant contribution of hepatocyte-derived FGF21 to fasting adaptation. However, this does not exclude the possibility that more prolonged starvation may elicit FGF21-dependent effects on metabolism and fertility, as reported in mouse models overexpressing FGF21 (Owen et al, 2013; Zhang et al, 2012). Our findings that circulating FGF21 exerts no detectable metabolic effects during fasting further underscore the divergence across its physiological, pathophysiological, and pharmacological actions (Kliewer and Mangelsdorf, 2019), the mechanistic basis of which remains incompletely understood.

## Methods

**Table:** Reagents and tools table

Reagent/resource	Reference or source	Identifier or catalog number
**Experimental models**		
Mouse: Albumin-Cre^−/−^*Pparα*^flox/flox^	(Montagner et al, 2016)	N/A
Mouse: Albumin-Cre^+/−^*Pparα*^flox/flox^	(Montagner et al, 2016)	N/A
Mouse: Albumin-Cre^−/−^*Fgf21*^flox/flox^	(Fougerat et al, 2022)	N/A
Mouse: Albumin-Cre^+/−^*Fgf21*^flox/flox^	(Fougerat et al, 2022)	N/A
**Oligonucleotides and other sequence-based reagents**		
Oligonucleotide sequences are listed in Appendix Table S2	This paper	Appendix Table S2
**Chemicals, enzymes, and other reagents**		
Takyon® Rox SYBR	Eurogentec	Cat#UF-LSMT-B0701
TRIzol Reagent	Invitrogen	Cat#TR118
FGF21 ELISA Kit	Sigma-Aldrich	Cat#ERKMFGF21-26K
Hot star Taq DNA Polymerase	Quiagen	Cat#203605
High-Capacity cDNA RT kit	Thermo Fisher	Cat#4368813
**Software**		
LinRegPCR (v2021.2)	(Ruijter et al, 2009)	http://LinRegPCR.nl
GraphPad Prism (v10.5.0)	GraphPad	https://www.graphpad.com
Metascape	(Zhou et al, 2019)	https://metascape.org
RStudio (v2024.04.1)	RStudio Team, PBC	https://posit.co
R (v4.5.1)	R Core Team	https://www.r-project.org
FactoMineR (v2.12)	(Lê et al, 2008)	
Factoextra (v1.0.7)	(Kassambara and Mundt, 2016)	
Tidyverse (v2.0.0)	(Wickham et al, 2019)	
Pheatmap (v1.0.13)	(Kolde, 2010)	
Illustrator (v28.7.8)	Adobe	https://www.adobe.com
Scan Control (v8.5.1)	Agilent	https://www.agilent.com
AriaMx (v2.0)	Agilent	https://www.agilent.com
**Deposited Data**		
Liver Microarray data	This paper	E-MTAB-15848
scWAT Microarray data	This paper	E-MTAB-15880
epWAT Microarray data	This paper	E-MTAB-15878
BAT Microarray data	This paper	E-MTAB-15855

### Methods and protocols

#### Mice

In vivo studies were performed in compliance with the European guidelines for the use and care of laboratory animals, and approved by an independent ethics committee under the authorization numbers 31741-2021052011598985. All mice were housed at 21–23 °C on a 12-h light (ZT0–ZT12)/12-h dark (ZT12–ZT24) cycle and had free access to the standard rodent diet (Safe 04 U8220G10R) and tap water. The ketogenic diet (TD. 96355, INOTIV), the 6% protein diet (TD.90016, INOTIV), the 40% protein diet (TD.90018, INOTIV) and the 70% carbohydrate diet (TD.98090, INOTIV) were used for mouse dietary intervention. Their compositions were listed in Appendix Table S1.

ZT stands for Zeitgeber time; ZT0 is defined as the time when the lights are turned on. Mice used in this study were sacrificed at ZT20, unless stated otherwise. All experiments were performed in 10–13-week-old mice. Before each experiment, the mouse body weight was recorded, and mice were randomly allocated to the different experimental groups.

*Fgf21* hepatocyte-specific knockout (*Fgf21*^*hep−/−*^) mice were generated at INRAE’s rodent facility (Toulouse, France) by mating the floxed-*Fgf21* mouse strain (B6.129S6(SJL)Fgf21 < tm1.2Djm >/J provided by The Jackson Laboratory) with C57BL/6J albumin-Cre transgenic mice, to obtain albumin-Cre^+/−^*Fgf21*^flox/flox^ mice, as described previously (Fougerat et al, 2022). Albumin-Cre^−/−^*Fgf21*^flox/flox^ (*Fgf21*^*hep+/+*^) littermates were used as controls.

*Pparα* hepatocyte-specific knockout (*Pparα*^*hep−/−*^) mice were generated at INRAE’s rodent facility (Toulouse, France) by mating the floxed-*Pparα* mouse strain with C57BL/6J albumin-Cre transgenic mice, as described previously (Montagner et al, 2016), to obtain albumin-Cre^+/−^*Pparα*^flox/flox^ mice. Albumin-Cre^−/−^*Pparα*^flox/flox^ (*Pparα*^*hep+/+*^) littermates were used as controls.

#### Genomic DNA

Genomic DNA was extracted from liver, subcutaneous white adipose tissue (scWAT), epididymal white adipose tissue (epWAT), brown adipose tissue (BAT), heart, brain, pancreas, gut, and muscle samples, which were stored at −80 °C following euthanasia. Tissue samples were homogenized in a solution containing 0.5 M EDTA, 5 M NaCl, 1 M Tris-HCl (pH 7.4), 10% SDS, and 20 mg/mL proteinase K (pH 8). The mixtures were incubated at 56 °C with continuous shaking at 300 rpm for 3 h. Following incubation, the samples were cooled on ice for 10 min and centrifuged. The supernatant was collected and mixed. For DNA extraction, 400 µL of a chloroform:phenol:isoamyl alcohol (24:25:1) was added to the samples, which were then centrifuged. The supernatant was transferred and mixed with 1 mL of 100% ethanol for DNA precipitation. Samples were incubated at −20 °C for 1 h before being washed with 70% ethanol. After ethanol removal, the samples were allowed to air dry for 1 h. DNA was subsequently resuspended in water prior to PCR. A total of 50 ng of genomic DNA was used for PCR amplification.

*Fgf21* deletion was confirmed by PCR using HotStart Taq Polymerase (Ozyme) and two forwards F (5′- AGTAGGGGTCAGACGTGGTG-3′), F2 (5’- TCAGACTCAGGAGTGCAGACAA-3’) and a reverse primer R (5′- TCAGACTCAGGAGTGCAGACAA-3′) (Fig. 1A). Amplification conditions were as follows with Touchdown PCR: 95 °C for 1 min; followed by nine cycles of 95 °C for 15 s, 65 °C for 15 s, 68 °C for 30 s, and 95 °C for 15 s, followed by 28 cycles of 60 °C for 15 s, 72 °C for 1 min, and 72 °C for 5 min. This reaction produced 500-bp fragments with exons 1,2, and 3 deletion (*Fgf21* deletion) and 352-bp (*Fgf21* floxed allele).

The albumin-Cre allele was detected by PCR using the following primer pairs: Mutant forward (Alb promoter) (5′- GAAGCAGAAGCTTAGGAAGATGG -3′, Wild-type forward (5′- TGGCTCGTTGTCCTTTGT -3′), and a common reverse (5′- TTGGCCCCTTACCATAACTG -3′). Amplification conditions were as follows: 95 °C for 1 min; followed by 35 cycles of 95 °C for 15 s, 60 °C for 15 s, and 72 °C for 30 s; and 72 °C for 5 min. This reaction produced 390-bp (mutant), 390-bp and 530 bp (heterozygote), and 530- bp (wild type) fragments.

#### DNA preparation for genotyping

DNA was extracted from tail tissue. Samples were mixed with 50 µL 25 mM NaOH, and 2 mM EDTA (pH 12), then incubated for 30 min at 95 °C. Samples were next, mixed, and neutralized with 50 µL 40 µM Tris-HCL (pH 5.0). After centrifugation, 7.6 µL of supernatant was used for PCR with HotStart Taq Polymerase (Ozyme) following the manufacturer’s instructions.

### Method details

#### Fasting kinetic experiments in Pparα hepatocyte-deficient and Fgf21 hepatocyte-deficient mouse model

Twelve-week-old male mice were fed *ad libitum* or fasted for 24 h (starting at ZT0), and their glycemia and ketonemia were monitored every 4 h. Plasma samples were prepared by centrifugation at 4000 rpm at 4 °C for 15 min. Body weight was monitored at ZT0 and ZT24 (*n* = 6–10 mice/genotype/experimental condition).

#### Fasting experiment in the Fgf21 hepatocyte-deficient mouse model

Thirteen-week-old male and female mice were fed *ad libitum* or fasted for 20 h (starting at ZT0) (*n* = 6–10 mice/genotype/experimental condition). Body weight, glycemia and ketonemia were measured at ZT20. After 20 h of fasting, mice were sacrificed by cervical dislocation. Plasma samples were prepared by centrifugation at 4000 rpm at 4 °C for 15 min. Organs and tissues were carefully collected, weighed, and frozen at −80 °C until subsequent analysis. Plasma FGF21 level peaked after 20 h of fasting. Therefore, we chose to fast mice for 20 h.

#### Ketogenic experiment in Fgf21 hepatocyte-deficient mouse model

Ten-week-old male mice were fed *ad libitum* standard diet or ketogenic diet for 9 days. On day 9, mice were sacrificed by cervical dislocation (*n* = 10 mice/genotype/experimental condition). Body weight, glycemia and ketonemia were measured. Plasma samples were prepared by centrifugation at 4000 rpm at 4 °C for 15 min. Organs and tissues were carefully collected, weighed, and frozen at −80 °C until subsequent analysis.

#### Indirect calorimetry

All animals were individually housed in a cage with lights on from 8 a.m. to 8 p.m. and an ambient temperature of 22 °C ± 0.5. Mice were acclimated to their calorimetric cages for 48 h before experimental measurements. After baseline recordings for 3 days, mice were fasted for 24 h and then refed *ad libitum*. Data were collected every 15 min. Total energy expenditure (kcal/h), oxygen consumption and carbon dioxide production (VO_2_ and VCO_2_, where V is the volume), respiratory exchange rate (RER = VCO_2_/VO_2_), and activity (counts/h) were measured using calorimetric cages (Labmaster, TSE Systems GmbH, Bad Homburg, Germany). Data analysis was carried out with Excel XP using extracted raw values of VO_2_ consumption, VCO_2_ production (mL/h), and energy expenditure (kcal/h). Subsequently, each value was expressed as a function of total lean tissue mass extracted from the EchoMRI analysis.

#### Bottle and food preference tests

All animals were individually housed in a cage. For the two-bottle sucrose preference assays, mice were allowed *ad libitum* access to a standard diet and were acclimated to cages with two bottles of just water for 3 days. Mice were then given access to bottles with water and water containing 10% sucrose (Sigma S9378). The position of the two bottles was changed every 2 days to exclude position effects. Consumption was measured daily during 4 days.

For the food-preference test, feeding behavior has been monitored using the BioDAQ system (Research Diets, Inc.). This automatic device allows continuous measurement of food intake for each animal. Before the experiment, calibration and quality control were done. For analyses, the minimum amount of food consumed by a mouse was set at 0.01 g. During the experiment, animals were singly housed at a constant temperature of 22.5 ± 1.0 °C, in a reversed light/night cycle (Off: 10:30–22:30). Cages were enriched with a nesting dome. Mice had free access to water and were fed *ad libitum*. Before the preference test, mice were fed with standard food (#A04, SAFE). After a 3-day acclimation period, food intake was recorded for 24 h on the standard diet. Since each cage was equipped with two feeders, food intake consisted of the sum of both.

The two food-preference tests were carried out after a 20-h fast, and was assessed over a 24-h refeeding period, beginning at the onset of the dark period (10:30 a.m.). For the first test, each mouse had free access to two feeders, containing either a low-protein diet (TD.90016, INOTIV) or a high-protein diet (TD.90018, INOTIV). At the end of the first test, mice were fed with a standard diet again for 12 days. For the second test, feeders contained either the low-protein diet (TD.90016, INOTIV) or another high-protein diet (TD.98090, INOTIV). During tests, the distribution of diets in the right or the left feeder was randomized for each mouse.

#### Blood and tissue sampling

Prior to sacrifice, the submandibular vein was lanced, and blood was collected into lithium heparin-coated tubes (BD Microtainer®, BD, Dutscher, Brumath, France). Then, mice were killed by cervical dislocation. Plasma was isolated by centrifugation (1500 × *g*, 15 min, 4 °C) and stored at −80 °C until biochemical analysis. Following sacrifice by cervical dislocation, the liver, subcutaneous white adipose tissue (scWAT), epididymal white adipose tissue (epWAT) and brown adipose tissue (BAT) were removed, weighed, and prepared for histology, or snap-frozen in liquid nitrogen and stored at −80 °C until use. Immediately after dissection, the pancreas was washed twice in cold HBSS 1X (without Ca^2+^ and Mg^2+^), then kept on ice during transfer to the cell culture facility. The pancreas was transferred to a sterile Petri dish containing 5 mL of HBSS 1X (without Ca^2+^ and Mg^2+^) (Gibco, 14175-095) and minced into small fragments (1–3 mm³) using sterile scissors. The fragments were transferred into a 50 mL conical tube and centrifuged at 400 × *g* for 2 min at 4 °C. The supernatant was carefully discarded to remove debris and blood cells. Tissue fragments were resuspended in 5 mL of freshly prepared collagenase II solution (Collagenase II (Sigma, C6885) 2000U/mL; HBSS 1X (with Ca^2+^ and Mg^2+^) (Gibco, 24020-117); HEPES (Gibco, 15630-080) 10 mM; Trypsin inhibitor (Sigma, Soybean 65035-M) 0.25 mg/mL) and incubated at 37 °C for 20–30 min. During incubation, the tissue was mechanically dissociated every 5 min by gentle pipetting, using pipettes of decreasing volume (25 mL, then 10 mL, then 5 mL). Once the tissue appeared homogeneously dissociated, enzymatic digestion was stopped by adding 10 mL of washing solution (HBSS 1X without Ca^2+^/Mg^2+^, 5% FBS, 10 mM HEPES). The suspension was centrifuged at 400 × *g* for 2 min at 4 °C. The supernatant containing collagenase was carefully removed. The pellet was resuspended in 5 mL of washing solution and filtered through a 100 µm sterile cell strainer placed over a new 50 mL conical tube. The filter was rinsed with an additional 5 mL of washing solution. The filtrate was centrifuged at 400 × *g* for 3 min at 4 °C, and the supernatant was discarded. The resulting pellet was resuspended in 1 mL of washing solution and transferred to a 1.5 mL microcentrifuge tube. A final centrifugation was performed at 450 × *g* for 5 min at 4 °C. The supernatant was removed, and the pellet was immediately snap-frozen and stored at –80 °C for subsequent RNA extraction.

#### Blood glucose and plasma analysis

Free fatty acids (FFAs) were determined from plasma samples using a COBASMIRA + biochemical analyzer (Genotoul Anexplo facility, Toulouse, France). Plasma glycerol was measured by enzymatic assay (Free Glycerol reagent, Sigma), and blood glucose was measured with an Accu-Chek Guide glucometer (Roche Diagnostics). β-hydroxybutyrate was measured with Optium β-ketone test strips that carried Optium Xceed sensors (Abbott Diabetes Care). Plasma FGF21 was assayed using the rat/mouse FGF21 ELISA kit (Sigma) according to the manufacturer’s instructions. Free carnitine and acylcarnitines were measured from plasma (10 µL) spotted on filter membranes (Protein Saver 903 cards; Whatman), dried, and then treated as reported (Chace et al, 1997). Briefly, acylcarnitines were derivatized to their butyl esters and treated with the reagents of the NeoGram MSMS-AAAC kit (PerkinElmer). Their analysis was conducted using a Waters 2795/Quattro Micro AP liquid chromatography–tandem mass spectrometer (Waters, Milford, MA).

#### Gene expression

Total cellular RNA from liver, scWAT, epWAT, BAT and pancreas was extracted with Tri Reagent® (Molecular Research Center, Inc., Cincinnati, OH, USA). RNAs were quantified using a nanophotometer N60 (Implen). Total RNA samples (2 μg) were then reverse transcribed using the High-Capacity cDNA Reverse Transcription kit (Applied BiosystemsTM) for real-time quantitative polymerase chain reaction (qPCR) analyses. The primers used for the SYBR Green assays are presented in Appendix Table S2. Amplifications were performed by the Takyon^TM^ DNA polymerase (Takyon^TM^ low row SYBR^®^ mastermix dNTP blue kit) on a Stratagene Mx3005P thermocycler (Agilent Technology, Santa Clara, CA, USA). The qPCR data were normalized to the level of TATA-box binding protein (TBP) messenger RNA (mRNA), and analysed with LinRegPCR software (v2021.2) to determine mean efficiency (NO), which was calculated as follows: NO = threshold/(Eff meanCq), where Eff mean: mean PCR efficiency, and Cq: quantification cycle.

Microarray experiments were conducted on *n* = 6 mice per group. Gene expression profiles were performed at the GeT-TRiX facility (GénoToul, Génopole Toulouse Midi-Pyrénées, France) using Sureprint G3 Mouse GE v2 microarrays (8 x 60 K, design 074809, Agilent Technologies) following the manufacturer’s instructions. For each sample, Cyanine-3 (Cy3) labeled cRNA was prepared from 200 ng of total RNA using the One-Color Quick Amp Labeling kit (Agilent Technology) according to the manufacturer’s instructions. Then purification was performed by Agencourt RNAClean XP (Agencourt Bioscience Corporation, Beverly, Massachusetts, USA). Dye incorporation and cRNA yield were checked using Dropsense™ 96 UV/VIS droplet reader (Trinean, Gent, Belgium). A total of 600 ng of Cy3-labeled cRNA were hybridized on the microarray slides following the manufacturer’s instructions. Immediately after washing, the slides were scanned on Agilent G2505C Microarray Scanner using Agilent Scan Control A.8.5.1 software. The fluorescence signal was extracted using Agilent Feature Extraction software v10.10.1.1 with default parameters.

#### Histology

4% formaldehyde-fixed, paraffin-embedded liver tissue was sliced into 5 µm sections and stained with hematoxylin and eosin (H&E). The staining was visualized with a light microscope equipped with a Nikon 90i camera. 4% formaldehyde-fixed, paraffin-embedded adipose tissues were sliced into 3 µm sections and stained with hematoxylin and eosin (H&E) and scanned (GénoToul, Génopole Toulouse Midi-Pyrénées, France).

#### Liver neutral lipids analysis

Hepatic lipids were extracted as previously described (BLIGH and DYER, 1959). Briefly, tissue samples were homogenized in Lysing Matrix D tubes with 1 ml methanol/5 mM EGTA (ethylene glycol-bis(β-aminoethyl ether)-*N*,*N*,*N*’,*N*’-tetraacetic acid) (2:1, v/v) in a FastPrep machine (MP Biochemicals). Lipids (corresponding to an equivalent of 2 mg of tissue) were extracted in chloroform/methanol/water (2.5:2.5:2, v/v/v), in the presence of the following internal standards: glyceryl trinonadecanoate, stigmasterol, and cholesteryl heptadecanoate (Sigma-Aldrich, Saint-Quentin-Fallavier, France). Total lipids were suspended in 160 µl ethyl acetate, and the triglycerides were analyzed with gas chromatography on a Focus Thermo Electron system using a Zebron-1 Phenomenex fused-silica capillary column (5 m, 0.32 mm i.d., 0.50 µm film thickness; Phenomenex, England), as previously described (Podechard et al, 2018) (GénoToul Metatoul-Lipidomiqie, Génopole Toulouse Midi-Pyrénées, France). The oven temperature was programmed to increase from 200° to 350 °C at a rate of 5 °C/min, and the carrier gas was hydrogen (0.5 bar). The injector and the two detectors were set to 315 and 345 °C, respectively.

#### Proton nuclear magnetic resonance (1H-NMR)-based metabolomics

Liver polar extracts were prepared and analysed using 1H-NMR-based metabolomics. All spectra were obtained on a Bruker DRX-600-Avance NMR spectrometer (Bruker) on the AXIOM metabolomics platform (MetaToul). Details on experimental procedures, data pretreatment and statistical analysis were described previously (Lukowicz et al, 2018).

#### Quantification and statistical analyses

Statistical analyses on biochemical and qPCR data were performed using GraphPad Prism for Windows (version 10.00; GraphPad Software). Two-way ANOVA was performed, followed by appropriate post hoc tests (Sidak’s multiple comparisons test or uncorrected Fisher’s LSD for food preference tests). Statistically significant post hoc differences are indicated on the graphs (* or # *p*_adj_ < 0.05; ** or ##*p*_adj_ < 0.01; *** or ###*p*_adj_ < 0.001; **** or ####*p*_adj_ < 0.0001). When only two groups were compared, the Student’s *t*-test was used or an unpaired *t*-test with Welch’s correction test for food preference tests. Statistically significant differences are indicated on the graphs (* or #*p* < 0.05, ** or ## *p* < 0.01, *** or ###*p* < 0.001, **** or ####*p* < 0.0001). Microarray data were analyzed using R and Bioconductor packages (Huber et al, 2015) as described in Array Express accession (E-MTAB-15848, E-MTAB-15855, E-MTAB-15880, and E-MTAB-15878). Raw data (median signal intensity) were filtered, log2 transformed, corrected for batch effects (microarray washing bath and labeling serials), and normalized using the quantile method (Bolstad et al, 2003). A model was fitted using the limma lmFit function (Ritchie et al, 2015). Pairwise comparisons between biological conditions were applied using specific contrasts. A correction for multiple testing was applied using the Benjamini–Hochberg procedure (Benjamini and Hochberg, 1995) to control the false discovery rate (FDR). Probes with an FDR <0.05 were considered to be differentially expressed between conditions. Hierarchical clustering was applied to the samples and the differentially expressed probes, using the 1-Pearson correlation coefficient as the distance metric and Ward’s criterion for agglomeration. The clustering results are illustrated as a heatmap of expression signals. Gene ontology and transcription factor enrichment analysis were performed using Metascape (Zhou et al, 2019). Microarray data identifying genes that are sensitive or insensitive to hepatocyte PPARα during fasting were obtained from (Montagner et al, 2016). Gene set enrichment analysis (GSEA) was performed to identify the leading-edge subsets.

### Graphics

The synopsis image was created with BioRender.

## Supplementary information

Appendix

Peer Review File

Source data Fig. 1

Source data Fig. 2

Source data Fig. 3

Source data Fig. 4

Source data Fig. 5

Source data Fig. 6

Expanded View Figures

## Data Availability

The datasets produced in this study are available in the following databases: all experimental details and microarray data were available in NCBI’s Gene Expression Omnibus (Edgar, 2002) and are accessible through Array Express accession (E-MTAB-15848, E-MTAB-15855, E-MTAB-15880, E-MTAB-15878) and are available at the following links, https://www.ebi.ac.uk/biostudies/arrayexpress/studies/E-MTAB-15848?key=a759354b-755e-4624-bf19-6c8f652bcd8d, https://www.ebi.ac.uk/biostudies/arrayexpress/studies/E-MTAB-15855?key=7a94d8a5-748e-40ba-98e9-2a4182ded2f0, https://www.ebi.ac.uk/biostudies/arrayexpress/studies/E-MTAB-15880?key=0c9f43d0-cd08-499e-a342-beb311b3c48d, https://www.ebi.ac.uk/biostudies/arrayexpress/studies/E-MTAB-15878?key=1739b37f-9c27-46f7-87a9-4de2dc45f68f, respectively. The source data of this paper are collected in the following database record: biostudies:S-SCDT-10_1038-S44319-026-00790-9.
