# Supplementary material for: Hepatic FGF21 is not required for fasting metabolism but guides protein appetite post energy depletion
Source: EMBO Rep. 2026 Apr 27;27(12):3189–213. doi: 10.1038/s44319-026-00790-9 (PMC13303862; doi:10.1038/s44319-026-00790-9)
Supplement: Supplementary file 1 — Appendix [file 44319_2026_790_MOESM1_ESM.pdf]

APPENDIX

Table

Appendix Figure S1. Hepatocyte-specific deletion of *Fgf21* does not affect fasting-induced metabolic responses in female mice. Related to Figures 1, 2 and 3..**2**

Appendix Figure S2. Hepatocyte-specific deletion of *Fgf21* does not affect metabolic responses to ketogenic diet in male mice. Related to Figures 1, 2 and 3..**4**

Appendix Figure S3. Hepatocyte-specific deletion of *Fgf21* does not affect autophagy gene expression in the liver of fed or fasted mice. Related to Figure 2..**6**

Appendix Figure S4. Hepatocyte-specific deletion of *Fgf21* does not affect hepatokine gene expression in the liver of fed and fasted mice. Related to Figure 3..**8**

Appendix Figure S5. Calorimetry for *Fgf21* liver floxed ($Fgf21^{hep+/+}$) or *Fgf21* liver knockout ($Fgf21^{hep-/-}$) male mice during 24 hours of feeding (Basal), 24 hours of fasting (Fasting), or 24 hours of refeeding (Refeeding). Related to Figure 6...**10**

Appendix Table S1. Diet composition...**12**

Appendix Table S2. Oligonucleotide sequences for real-time qPCR..**13**

Appendix Figure S1.

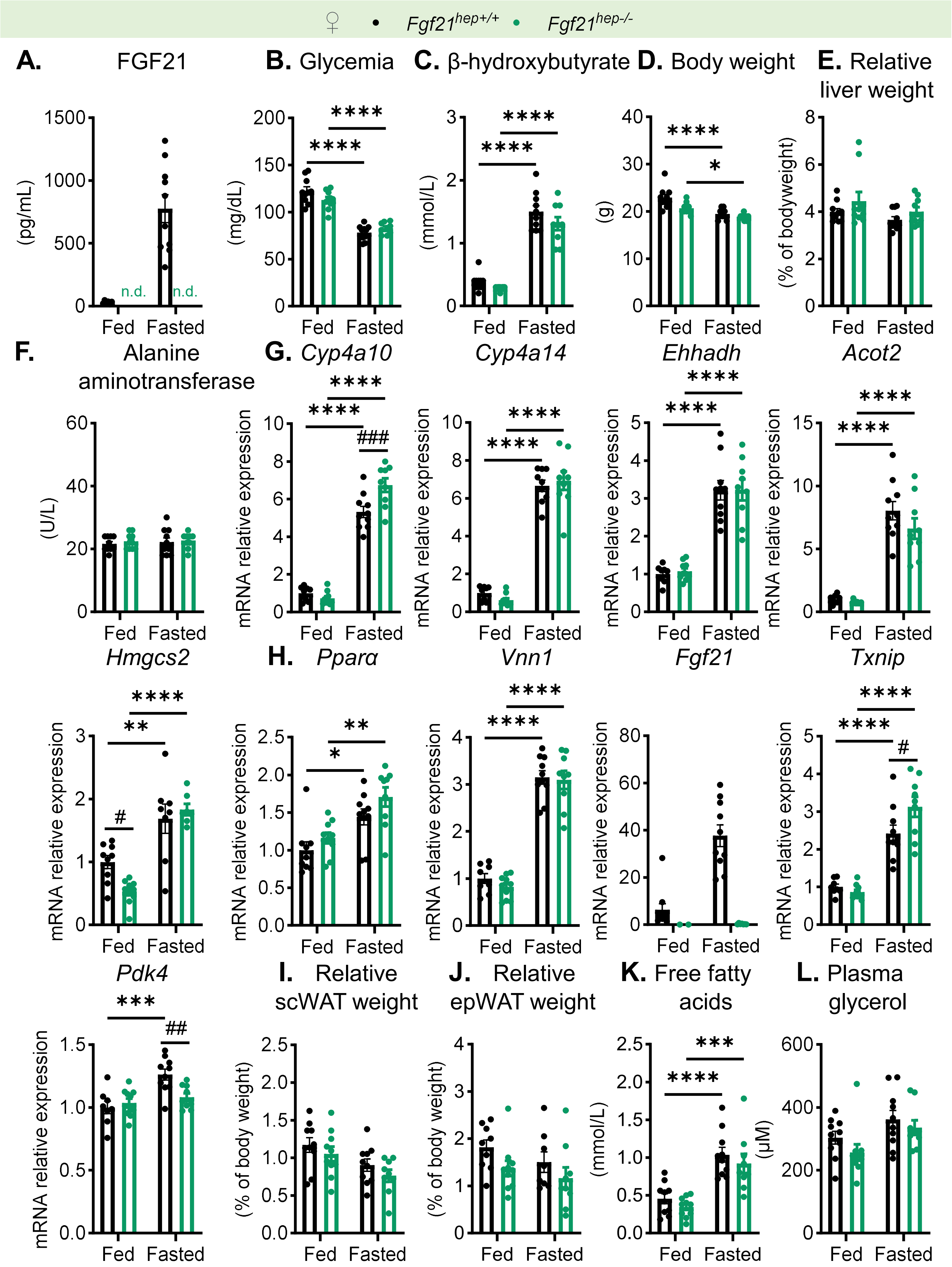

Appendix Figure S1. Hepatocyte-specific deletion of *Fgf21* does not affect fasting-induced metabolic responses in female mice. Related to Figures 1, 2 and 3.
Fgf21 liver floxed ($Fgf21^{hep+/+}$) or *Fgf21* liver knockout ($Fgf21^{hep-/-}$) female mice were fed *ad libitum* or fasted for 20 hours.

(A) FGF21 plasma level was determined by ELISA (n=9-10 mice per group, biological replicates).

(B) Blood glucose levels (n=9-10 mice per group, biological replicates, two-way ANOVA followed by Šídák's multiple comparisons test α=0.05, Fed *vs* Fasted in $Fgf21^{hep+/+}$ or $Fgf21^{hep-/-}$, ****p_{adj}<0.0001).

(C) Plasma level of β-hydroxybutyrate (n=9-10 mice per group, biological replicates, two-way ANOVA followed by Šídák's multiple comparisons test α=0.05, Fed *vs* Fasted in $Fgf21^{hep+/+}$ or $Fgf21^{hep-/-}$, ****p_{adj}<0.0001).

(D) Body weight was measured after 20 hours of fasting (n=9-10 mice per group, biological replicates, two-way ANOVA followed by Šídák's multiple comparisons test α=0.05, Fed *vs* Fasted in $Fgf21^{hep+/+}$ or $Fgf21^{hep-/-}$, ****p_{adj}<0.0001, *p_{adj}=0.0157).

(E) Relative liver weight (n=9-10 mice per group, biological replicates, two-way ANOVA followed by Šídák's multiple comparisons test α=0.05).

(F) Plasma alanine aminotransferase levels (n=9-10 mice per group, biological replicates, two-way ANOVA followed by Šídák's multiple comparisons test α=0.05).

(G-H) mRNA relative expression of *Cyp4a10*, *Cyp4a14*, *Ehhadh*, *Acot2*, *Hmgcs2* **(G)** and *Pparα*, *Vnn1*, *Fgf21*, *Txnip*, *Pdk4* **(H)** in liver samples measured by qRT-PCR (n=8-10 mice per group, biological replicates, two-way ANOVA followed by Šídák's multiple comparisons test α=0.05, Fed *vs* Fasted in $Fgf21^{hep+/+}$ or $Fgf21^{hep-/-}$, ****p_{adj}<0.0001; *Hmgcs2*, **p_{adj}=0.0019; *Pparα*, *p_{adj}=0.0113, **p_{adj}=0.0018; *Pdk4*, ***p_{adj}=0.0002; Fasted $Fgf21^{hep+/+}$ *vs* Fasted $Fgf21^{hep-/-}$, *Cyp4a10*, ###p_{adj}=0.0007; *Txnip*, #p_{adj}=0.0194; *Pdk4*, ##p_{adj}=0.0062; Fed $Fgf21^{hep+/+}$ *vs* Fed $Fgf21^{hep-/-}$, *Hmgcs2*, #p_{adj}=0.0258).

(I-J) Relative sub-cutaneous white adipose tissue (scWAT) weight **(I)** and epididymal white adipose tissue (epWAT) weight **(J)** (n=8-10 mice per group, biological replicates, two-way ANOVA followed by Šídák's multiple comparisons test α=0.05).

(K) Plasma free fatty acids (n=9-10 mice per group, biological replicates, two-way ANOVA followed by Šídák's multiple comparisons test α=0.05, Fed *vs* Fasted in $Fgf21^{hep+/+}$ or $Fgf21^{hep-/-}$, ****p_{adj}<0.0001, ***p_{adj}=0.0001).

(L) Plasma level of glycerol (n=9-10 mice per group, biological replicates, two-way ANOVA followed by Šídák's multiple comparisons test α=0.05).

Data information: All data are presented as mean ± SEM; * shows a fasting effect; # shows a genotype effect. n.d.: not detected.

Appendix Figure S2.

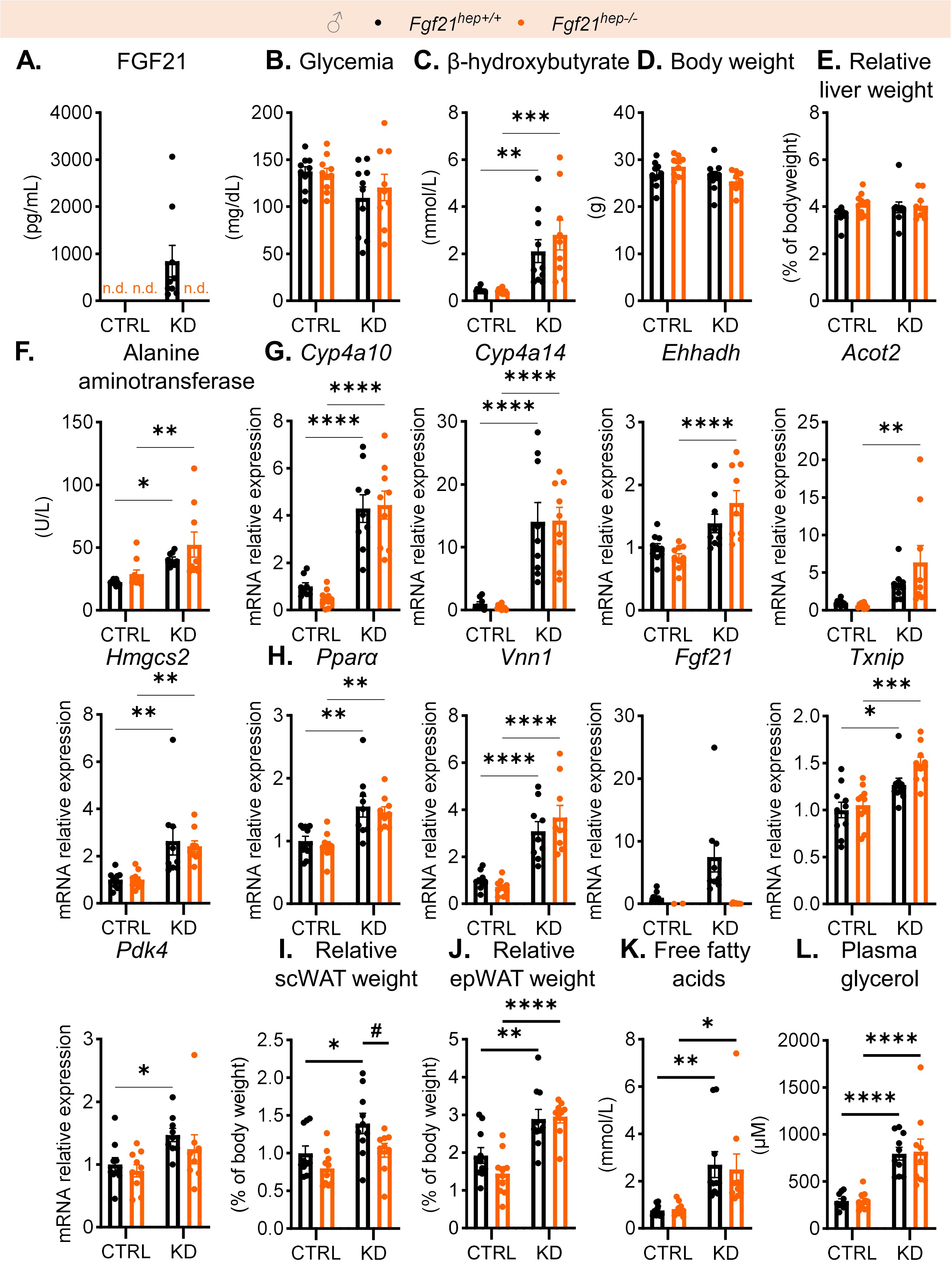

Appendix Figure S2. Hepatocyte-specific deletion of *Fgf21* does not affect metabolic responses to ketogenic diet in male mice. Related to Figures 1, 2 and 3.
Fgf21 liver floxed (*Fgf21*$^{hep+/+}$) or *Fgf21* liver knockout (*Fgf21*$^{hep-/-}$) female mice were fed a control diet (CTRL) or a ketogenic diet (KD) ad libitum for 9 days.

(A) FGF21 plasma level was determined by ELISA (n=9-10 mice per group, biological replicates).

(B) Blood glucose levels (n=9-10 mice per group, biological replicates, two-way ANOVA followed by Šídák's multiple comparisons test α=0.05).

(C) Plasma level of β-hydroxybutyrate (n=9-10 mice per group, biological replicates, two-way ANOVA followed by Šídák's multiple comparisons test α=0.05, CTRL *vs* KD in *Fgf21*$^{hep+/+}$ or *Fgf21*$^{hep-/-}$, **p_{adj}=0.0077, ***p_{adj}=0.002).

(D) Body weight was measured after 20 hours of fasting (n=9-10 mice per group, biological replicates, two-way ANOVA followed by Šídák's multiple comparisons test α=0.05).

(E) Relative liver weight (n=9-10 mice per group, biological replicates, two-way ANOVA followed by Šídák's multiple comparisons test α=0.05).

(F) Plasma alanine aminotransferase levels (n=9-10 mice per group, biological replicates, two-way ANOVA followed by Šídák's multiple comparisons test α=0.05, CTRL *vs* KD in *Fgf21*$^{hep+/+}$ or *Fgf21*$^{hep-/-}$, *p_{adj}=0.0349, **p_{adj}=0.0057).

(G-H) mRNA relative expression of *Cyp4a10*, *Cyp4a14*, *Ehhadh*, *Acot2*, *Hmgcs2* **(G)** and *Pparα*, *Vnn1*, *Fgf21*, *Txnip*, *Pdk4* **(H)** in liver samples measured by qRT-PCR (n=8-10 mice per group, biological replicates, two-way ANOVA followed by Šídák's multiple comparisons test α=0.05, CTRL *vs* KD in *Fgf21*$^{hep+/+}$ or *Fgf21*$^{hep-/-}$, ****p_{adj}<0.0001; *Acot2*, **p_{adj}=0.0018; *Hmgcs2*, **p_{adj}=0.0014 (*Fgf21*$^{hep+/+}$), **p_{adj}=0.0053 (*Fgf21*$^{hep-/-}$); *Pparα*, **p_{adj}=0.0013 (*Fgf21*$^{hep+/+}$), **p_{adj}=0.0016 (*Fgf21*$^{hep-/-}$); *Txnip*, *p_{adj}=0.0309, ***p_{adj}=0.0003; *Pdk4*, *p_{adj}=0.0376).

(I-J) Relative sub-cutaneous white adipose tissue (scWAT) weight **(I)** and epididymal white adipose tissue (epWAT) weight **(J)** (n=9-10 mice per group, biological replicates, two-way ANOVA followed by Šídák's multiple comparisons test α=0.05, scWAT, CTRL *vs* KD in *Fgf21*$^{hep+/+}$ or *Fgf21*$^{hep-/-}$, *p_{adj}=0.0184, **p_{adj}=0.0037, ****p_{adj}<0.0001; KD *Fgf21*$^{hep+/+}$ *vs* KD *Fgf21*$^{hep-/-}$, #p_{adj}=0.0413).

(K) Plasma free fatty acids (n=9-10 mice per group, biological replicates, two-way ANOVA followed by Šídák's multiple comparisons test α=0.05, CTRL *vs* KD in *Fgf21*$^{hep+/+}$ or *Fgf21*$^{hep-/-}$, **p_{adj}=0.0043, *p_{adj}=0.0171).

(L) Plasma level of glycerol (n=9-10 mice per group, biological replicates, two-way ANOVA followed by Šídák's multiple comparisons test α=0.05, CTRL *vs* KD in *Fgf21*$^{hep+/+}$ or *Fgf21*$^{hep-/-}$, ****p_{adj}<0.0001).

Data information: All data are presented as mean ± SEM; * shows a fasting effect; # shows a genotype effect. n.d.: not detected.

Appendix Figure S3.

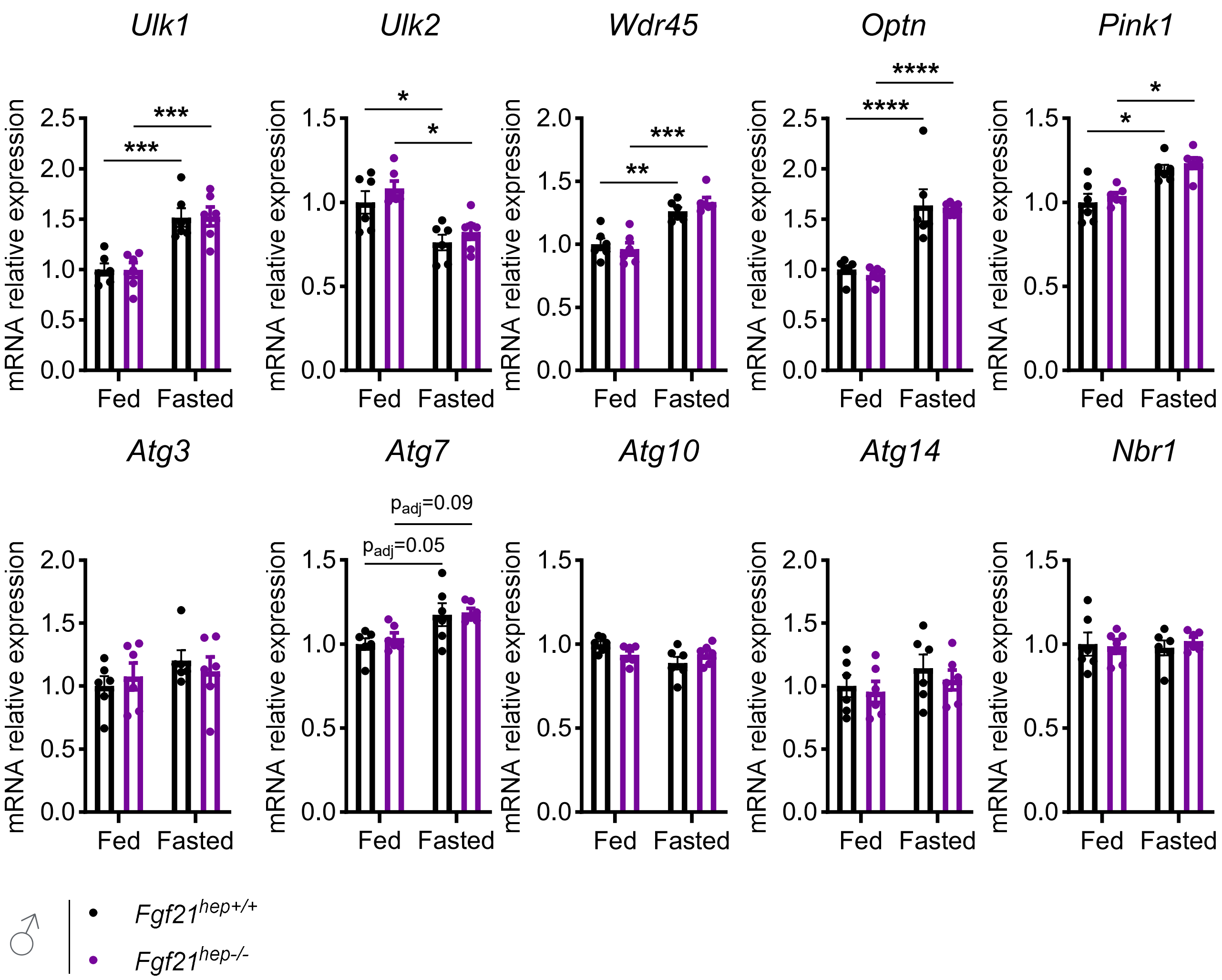

Appendix Figure S3. Hepatocyte-specific deletion of *Fgf21* does not affect autophagy gene expression in the liver of fed or fasted mice. Related to Figure 2.

Microarray experiment performed with liver samples from *Fgf21* liver floxed (*Fgf21*$^{hep+/+}$) or *Fgf21* liver knockout (*Fgf21*$^{hep-/-}$) mice were fed *ad libitum* or fasted for 20 hours. Bar graphs show the mRNA relative expression of liver *Ulk1*, *Ulk2*, *Wdr45*, *Optn*, *Pink1*, *Atg3*, *Atg7*, *Atg10*, *Atg14* and *Nbr1*, derived from microarray results (n=6 mice per group, biological replicates, for each represented gene: limma package, with linear models fitted (lmFit), Fed *vs* Fasted in *Fgf21*$^{hep+/+}$ or *Fgf21*$^{hep-/-}$, *Ulk1*, ***p_{adj}=0.0007 (*Fgf21*$^{hep+/+}$), ***p_{adj}=0.0006 (*Fgf21*$^{hep-/-}$); *Ulk2*, *p_{adj}=0.01 (*Fgf21*$^{hep+/+}$), *p_{adj}=0.011 (*Fgf21*$^{hep-/-}$); *Wdr45*, **p_{adj}=0.003, ***p_{adj}=0.0001; *Optn*, ****p_{adj}<0.0001; *Pink1*, *p_{adj}=0.01 (*Fgf21*$^{hep+/+}$), *p_{adj}=0.019 (*Fgf21*$^{hep-/-}$)).

Data information: All data are presented as mean ± SEM; * shows a fasting effect.

Appendix Figure S4.

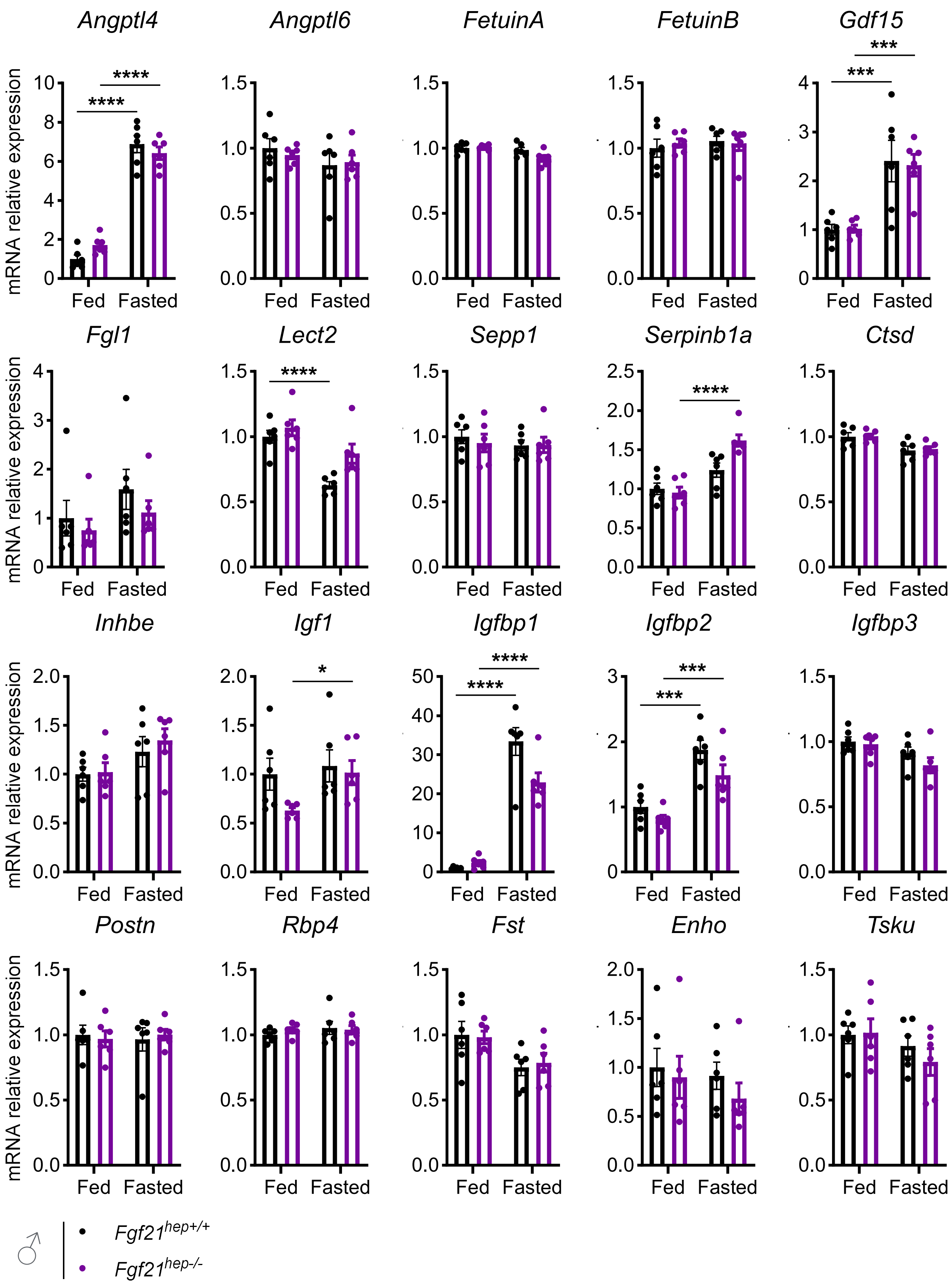

Appendix Figure S4. Hepatocyte-specific deletion of *Fgf21* does not affect hepatokine gene expression in the liver of fed and fasted mice. Related to Figure 3.

Microarray experiment performed with liver samples from *Fgf21* liver floxed (*Fgf21*$^{hep+/+}$) or *Fgf21* liver knockout (*Fgf21*$^{hep-/-}$) mice were fed *ad libitum* or fasted for 20 hours. Bar graphs show the mRNA relative expression of liver *Angptl4*, *Angptl6*, *FetuinA*, *FetuinB*, *Gdf15*, *Fgl1*, *Lect2*, *Sepp1*, *Serpinb1a*, *Ctsd*, *Inhbe*, *Igf1*, *Igfbp1*, *Igfbp2*, *Igfbp3*, *Postn*, *Rbp4*, *Fst*, *Enho*, and *Tsku*, derived from microarray results (n=6 mice per group, biological replicates, for each represented gene: limma package, with linear models fitted (lmFit), Fed *vs* Fasted in *Fgf21*$^{hep+/+}$ or *Fgf21*$^{hep-/-}$, ****p_{adj}<0.0001; *Gdf15*, ***p_{adj}=0.0006 (*Fgf21*$^{hep+/+}$), ***p_{adj}=0.0009 (*Fgf21*$^{hep-/-}$); *Igf1*, *p_{adj}=0.036; *Igfbp2*, ***p_{adj}=0.0001 (*Fgf21*$^{hep+/+}$), ***p_{adj}=0.0005 (*Fgf21*$^{hep-/-}$)).

Data information: All data are presented as mean ± SEM; * shows a fasting effect.

Appendix Figure S5.

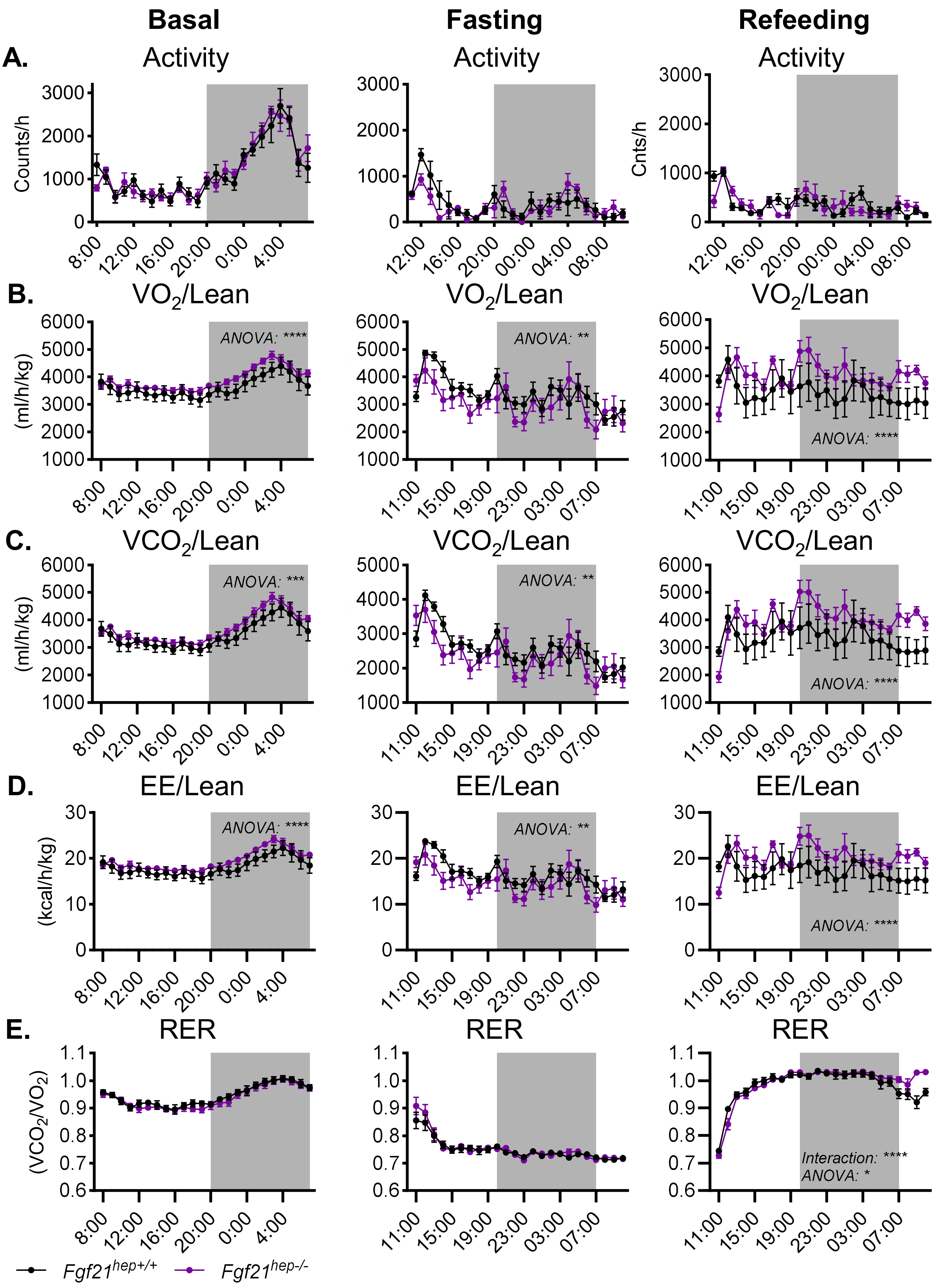

Appendix Figure S5. Calorimetry for *Fgf21* liver floxed (*Fgf21*$^{hep+/+}$) or *Fgf21* liver knockout (*Fgf21*$^{hep-/-}$) male mice during 24 hours of feeding (Basal), 24 hours of fasting (Fasting), or 24 hours of refeeding (Refeeding). Related to Figure 6.

(A) Locomotor activity calculated during the light and dark phases (n=5-6 mice per group, biological replicates, two-way ANOVA followed by Šídák's multiple comparisons test α=0.05).

(B) Rate of VO_2 consumption normalized per lean body mass (n=5-6 mice per group, biological replicates, two-way ANOVA followed by Šídák's multiple comparisons test α=0.05, ****$p_{genotype}<0.0001$ (Basal); **$p_{genotype}=0.0024$ (Fasting); ****$p_{genotype}<0.0001$ (Refeeding)).

(C) Rate of VCO_2 consumption normalized per lean body mass (n=5-6 mice per group, biological replicates, two-way ANOVA followed by Šídák's multiple comparisons test α=0.05, ***$p_{genotype}=0.0004$ (Basal); **$p_{genotype}=0.0064$ (Fasting); ****$p_{genotype}<0.0001$ (Refeeding)).

(D) Energy expenditure (EE) normalized per lean body mass (n=5-6 mice per group, biological replicates, two-way ANOVA followed by Šídák's multiple comparisons test α=0.05, ****$p_{genotype}<0.0001$ (Basal); **$p_{genotype}=0.0028$ (Fasting); ****$p_{genotype}<0.0001$ (Refeeding)).

(E) Respiratory exchange ratio (RER) (n=5-6 mice per group, biological replicates, two-way ANOVA followed by Šídák's multiple comparisons test α=0.05, *$p_{genotype}=0.016$ (Refeeding)).

Data information: All data are presented as mean ± SEM; * shows a genotype effect from ANOVA results.

Appendix Table S1. Diet composition.

Regimen	CTRL	Ketogenic diet	6% protein diet	40% protein diet	70% carbohydrate diet
**Product number**	A04<br>Safe	TD.96355<br>Envigo	TD.90016<br>Envigo	TD.90018<br>Envigo	TD.98090<br>Envigo
**Protein (% kcal from)**	19	9.2	6.5	42.6	17.8
**Carbohydrate (% kcal from)**	72	0.3	80.4	44.3	70.4
**Sucrose (g/kg)**	26.35	NA	571.8	231.82	645.6
**Corn Strach (g/kg)**	318	NA	200	200	20
**Fat (% kcal from)**	8	90.5	13.1	13.1	11.8
**Kcal/g**	3.3	6.7	3.8	3.8	4

Appendix Table S2. Oligonucleotide sequences for real-time qPCR.

Gene	NCBI Refseq	Forward primer	Reverse primer
Acot2	NM_134188	TGGAGTACTTTGAAGAAGCCGTG	CATTTTGACTTGGTTTCTCAGAAGG
β-klotho	NM_031180	GATGAAGAATTTCCTAAACCAGGTT	AACCAAACACGCGGATTTC
Cyp4a10	NM_010011	TCCAGCAGTTCCCATCACCT	TTGCTTCCCCAGAACCATCT
Cyp4a14	NM_007822	TCAGTCTATTTCTGGTGCTGTTC	GAGCTCCTTGTCCTTCAGATGGT
Ehhadh	NM_023737	CGTCTCCTCGGTTGGTGTTC	ATTATCTTCTTTGCAGTATCTAGCTGCTT
Fgf21	NM_020013	AAAGCCTCTAGGTTTCTTTGCCA	CCTCAGGATCAAAGTGAGGCG
Fgf21 **(F: wild type)**	NM_020013	GAAACAAAGCTTCAAAATAGGG	AGTAGGGGTCAGACGTGGTG
Fgf21 **(F2: floxed allele)**	NM_020013	TCAGACTCAGGAGTGCAGACAA	
Fgfr1c	NM_010206	GCCAGACAACTTGCCGTATG	ATTTCCTTGTCGGTGGTATTAACTC
G6pc	NM_008061	CTCACTTTCCCCACCAGGTC	GCTGAAAGTTTCAGCCACAGC
Hmgcs2	NM_008256	TGCAGGAAACTTCGCTCACA	AAATAGACCTCCAGGGCAAGGA
Pck1	NM_011044	TCCGCAAGCTGAAGAAATATGA	TGATGATGACTGTCTTGCTTTCG
Pdk4	NM_013743	ATCGCCAGAATTAAACCTCACAC	TGGATTGGTTGGCCTGGA
Pparα	NM_011144	CCCTGTTTGTGGCTGCTATAATTT	GGGAAGAGGAAGGTGTCATCTG
Txnip	NM_023719	GACTGGAGAGCCCCACCAC	GGACGCAGGGATCCACCTCA
Vnn1	NM_011704	ATGAGGTTTATGCCTTTGGAGC	CCACAGGTGCGTAAATTGGTAG
