# Supplementary material for: Hepatic FGF21 is not required for fasting metabolism but guides protein appetite post energy depletion
Source: EMBO Rep. 2026 Apr 27;27(12):3189–213. doi: 10.1038/s44319-026-00790-9 (PMC13303862; doi:10.1038/s44319-026-00790-9)
Supplement: Supplementary file 3 — Source data Fig. 1 [file 44319_2026_790_MOESM3_ESM.zip › Figure 1/1A/Agarose gel annotated.pptx]

## Slide 1
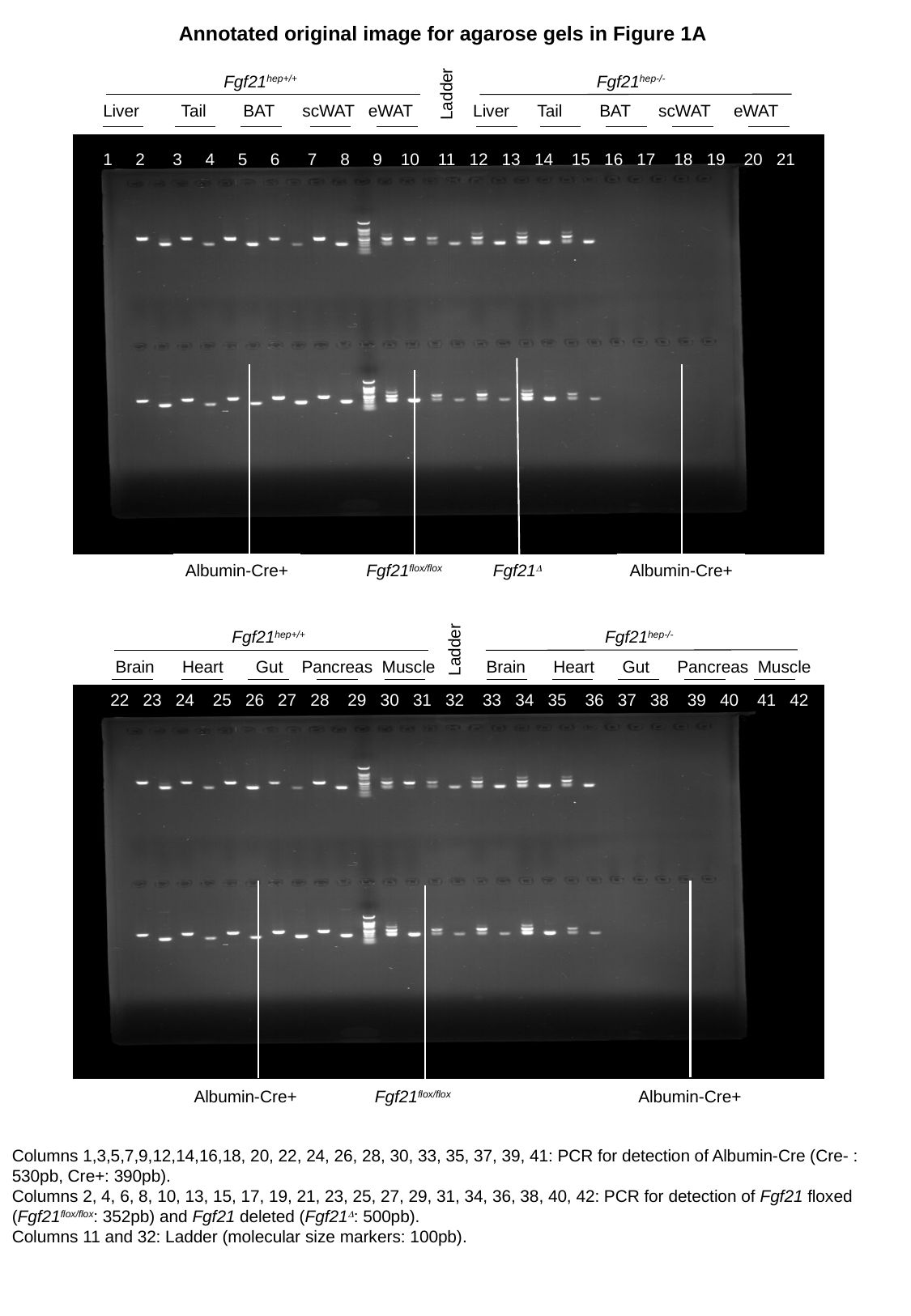

Annotated original image for agarose gels in Figure 1A
Fgf21hep+/+
Fgf21hep-/-
Ladder
Liver Tail BAT scWAT eWAT Liver Tail BAT scWAT eWAT
1 2 3 4 5 6 7 8 9 10 11 12 13 14 15 16 17 18 19 20 21
Albumin-Cre+
Fgf21flox/flox
Fgf21D
Albumin-Cre+
Fgf21hep+/+
Fgf21hep-/-
Ladder
Brain Heart Gut Pancreas Muscle Brain Heart Gut Pancreas Muscle
22 23 24 25 26 27 28 29 30 31 32 33 34 35 36 37 38 39 40 41 42
Albumin-Cre+
Fgf21flox/flox
Albumin-Cre+
Columns 1,3,5,7,9,12,14,16,18, 20, 22, 24, 26, 28, 30, 33, 35, 37, 39, 41: PCR for detection of Albumin-Cre (Cre- : 530pb, Cre+: 390pb).
Columns 2, 4, 6, 8, 10, 13, 15, 17, 19, 21, 23, 25, 27, 29, 31, 34, 36, 38, 40, 42: PCR for detection of Fgf21 floxed (Fgf21flox/flox: 352pb) and Fgf21 deleted (Fgf21D: 500pb).
Columns 11 and 32: Ladder (molecular size markers: 100pb).
