# Supplementary figures and images for: Hepatic FGF21 is not required for fasting metabolism but guides protein appetite post energy depletion
Source: EMBO Rep. 2026 Apr 27;27(12):3189–213. doi: 10.1038/s44319-026-00790-9 (PMC13303862; doi:10.1038/s44319-026-00790-9)

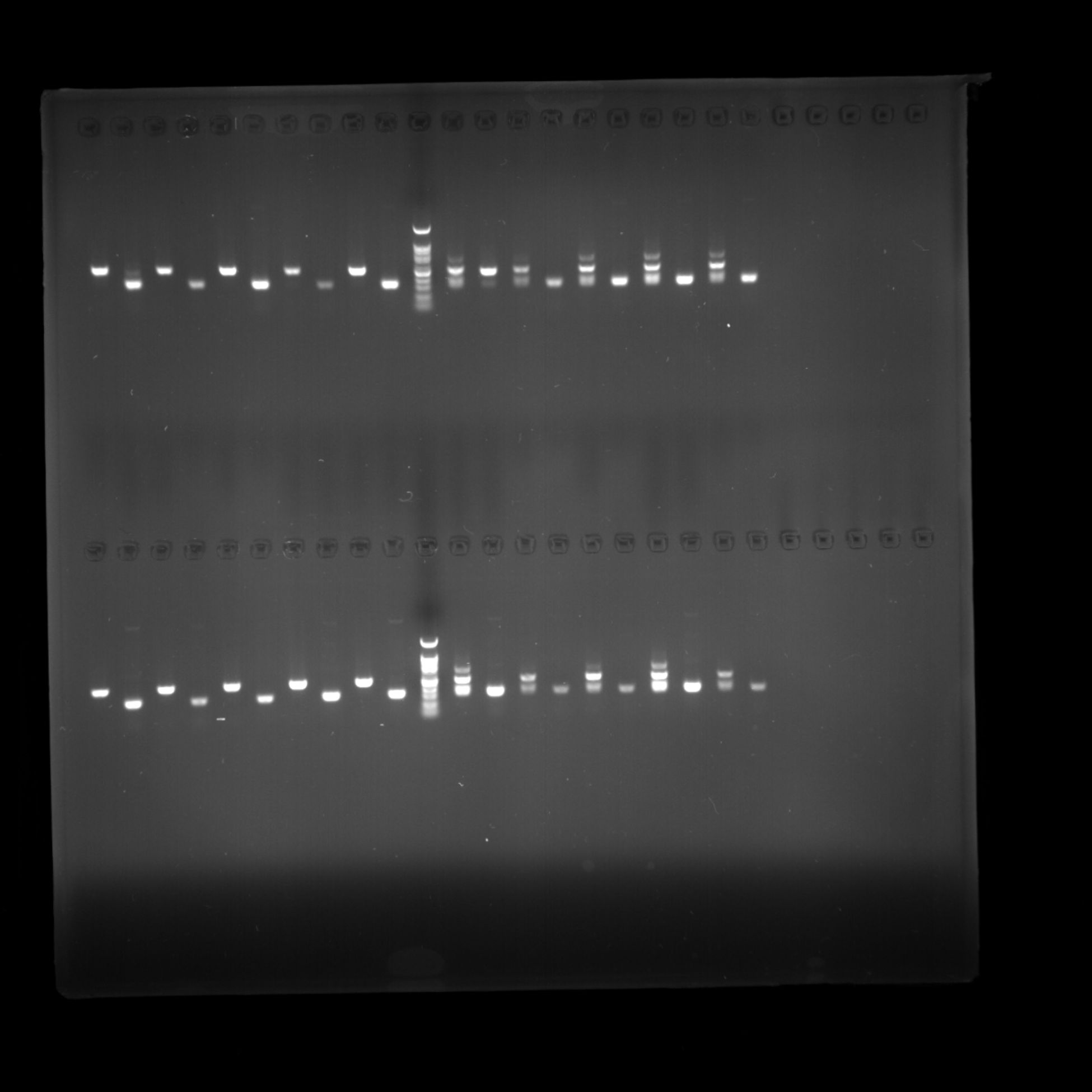

Supplement: Supplementary file 3 — Source data Fig. 1 [file 44319_2026_790_MOESM3_ESM.zip › Figure 1/1A/Agarose gel.Tif]

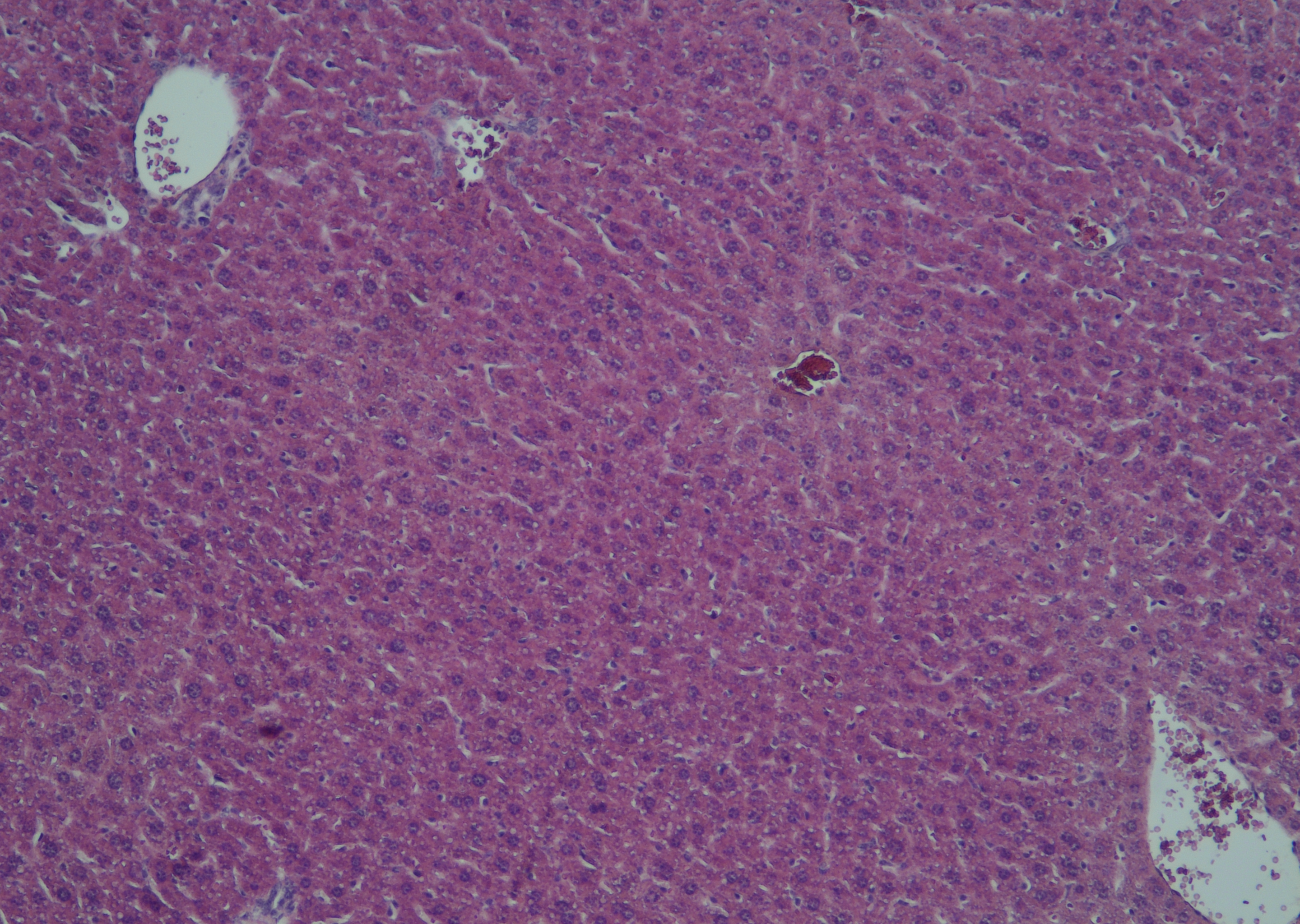

Supplement: Supplementary file 4 — Source data Fig. 2 [file 44319_2026_790_MOESM4_ESM.zip › Figure 2/2D/Liver FGF21LKO Fasted.tif]

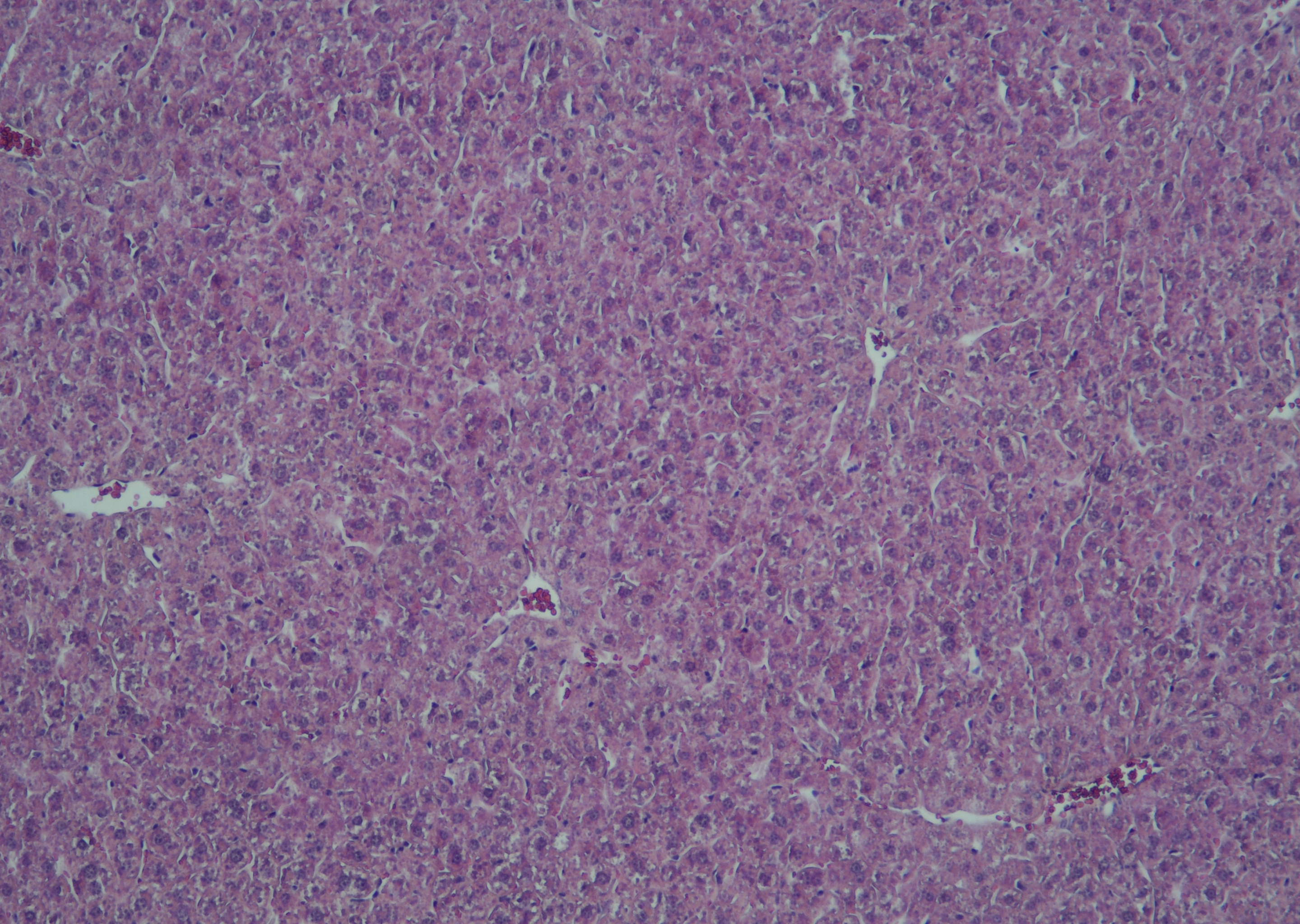

Supplement: Supplementary file 4 — Source data Fig. 2 [file 44319_2026_790_MOESM4_ESM.zip › Figure 2/2D/Liver FGF21LKO Fed.tif]

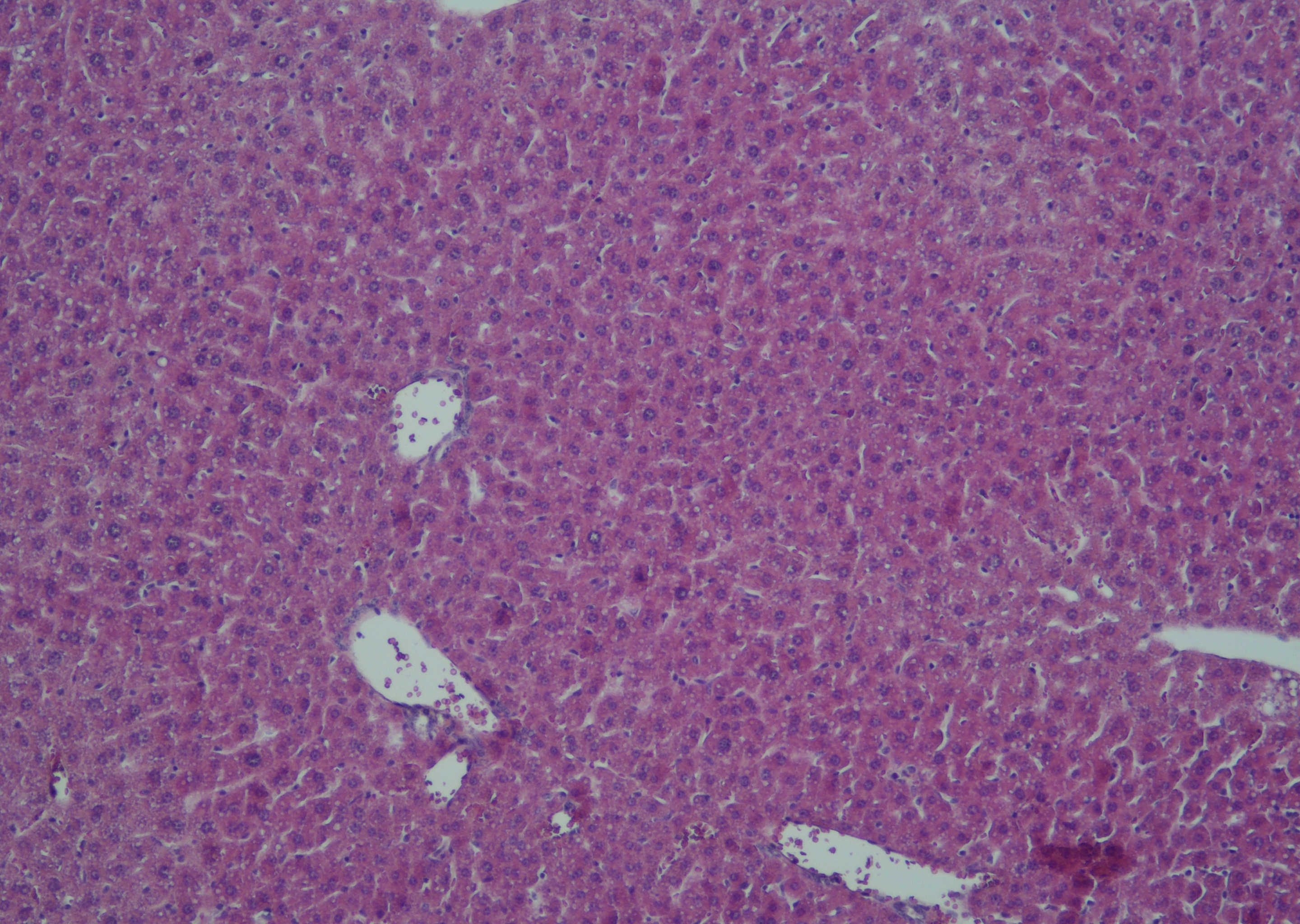

Supplement: Supplementary file 4 — Source data Fig. 2 [file 44319_2026_790_MOESM4_ESM.zip › Figure 2/2D/Liver FGF21LWT Fasted.tif]

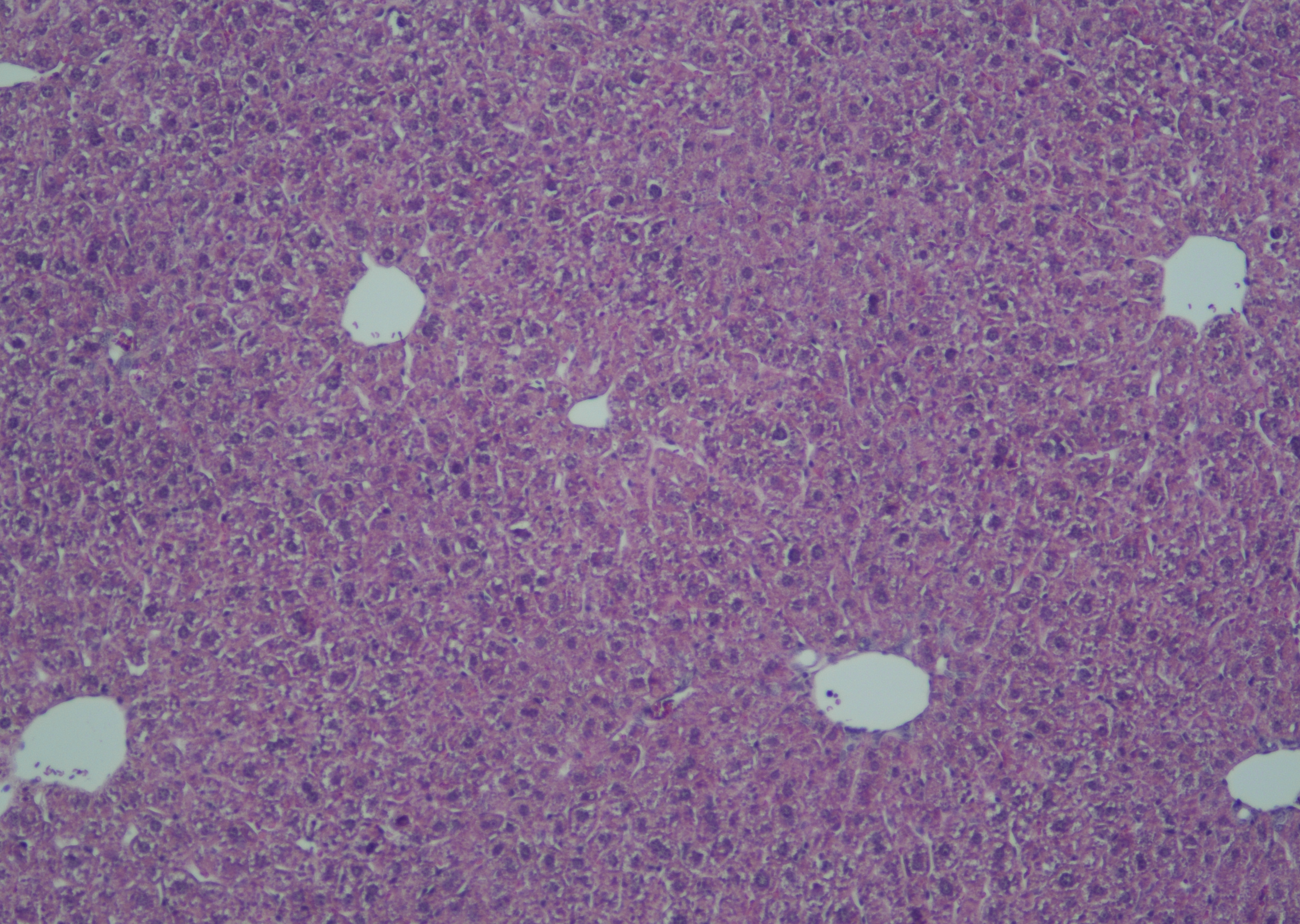

Supplement: Supplementary file 4 — Source data Fig. 2 [file 44319_2026_790_MOESM4_ESM.zip › Figure 2/2D/Liver FGF21LWT Fed.tif]

Scree plot

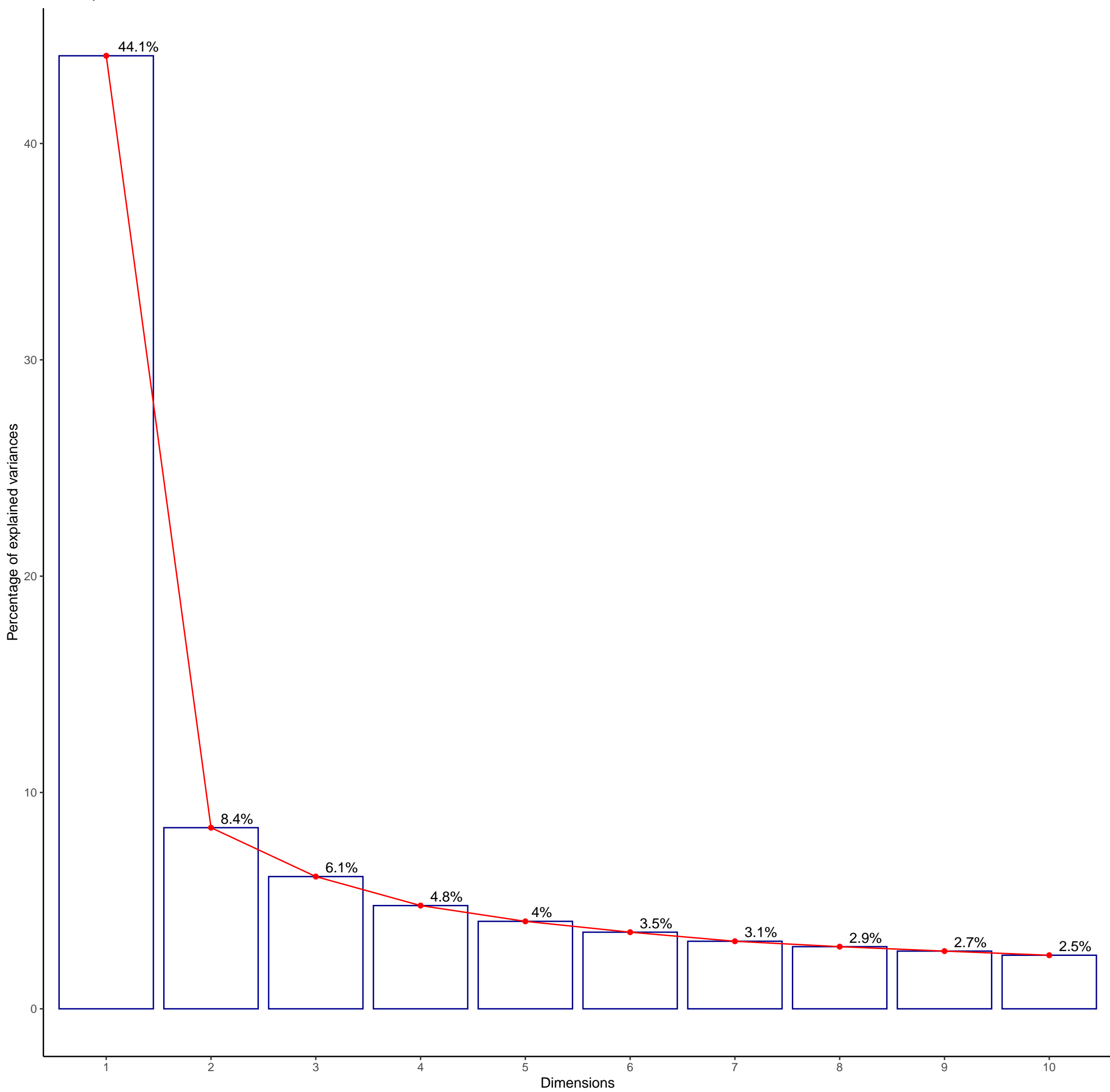

Supplement: Supplementary file 5 — Source data Fig. 3 [file 44319_2026_790_MOESM5_ESM.zip › Figure 3/3A/Scree_plot_dim_PCA_liver.pdf]

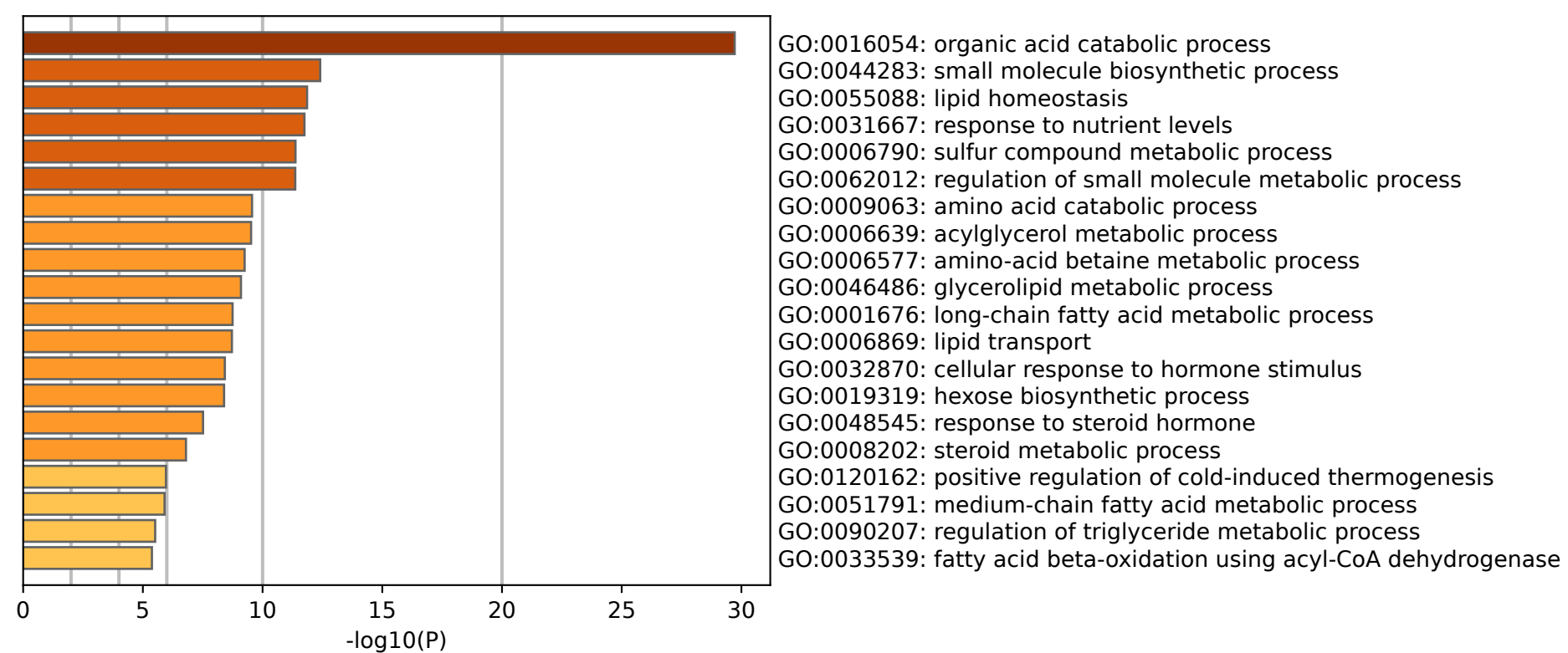

Supplement: Supplementary file 5 — Source data Fig. 3 [file 44319_2026_790_MOESM5_ESM.zip › Figure 3/3D/HeatmapSelectedGO cluster1_liver.pdf]

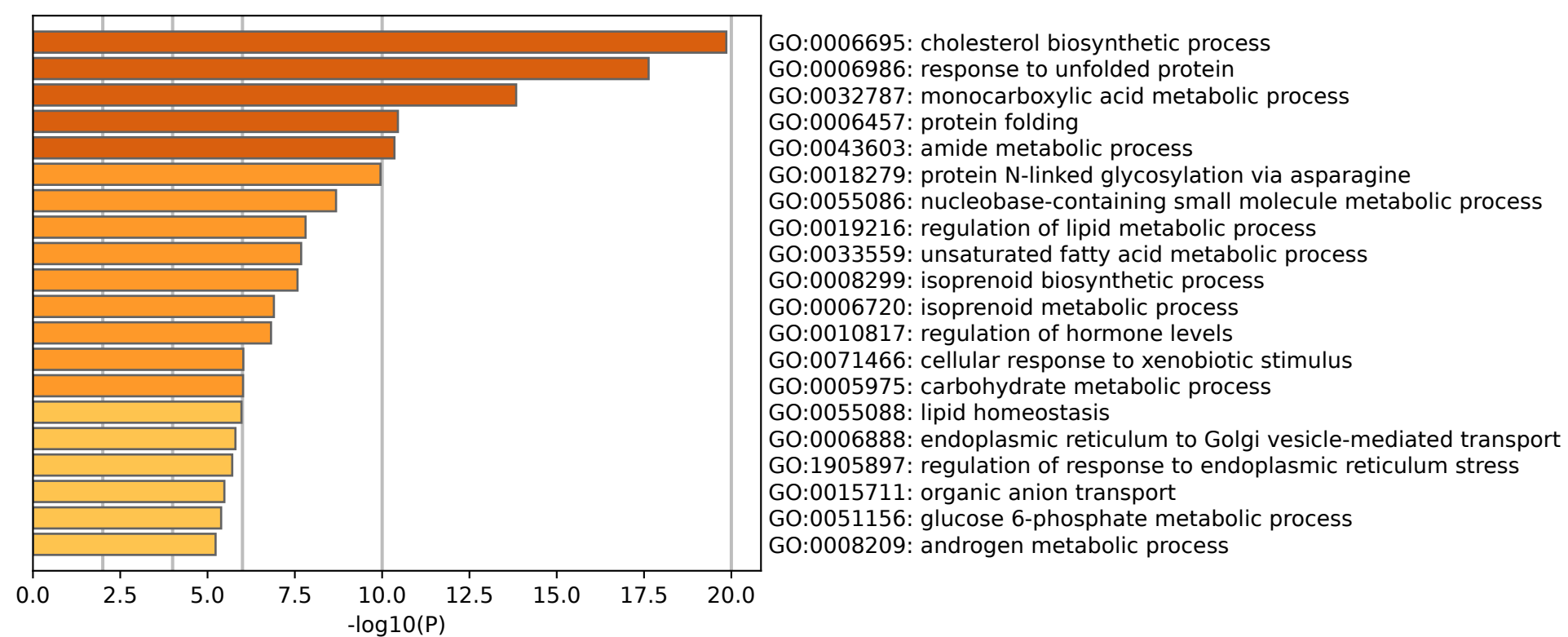

Supplement: Supplementary file 5 — Source data Fig. 3 [file 44319_2026_790_MOESM5_ESM.zip › Figure 3/3D/HeatmapSelectedGO cluster2_liver.pdf]

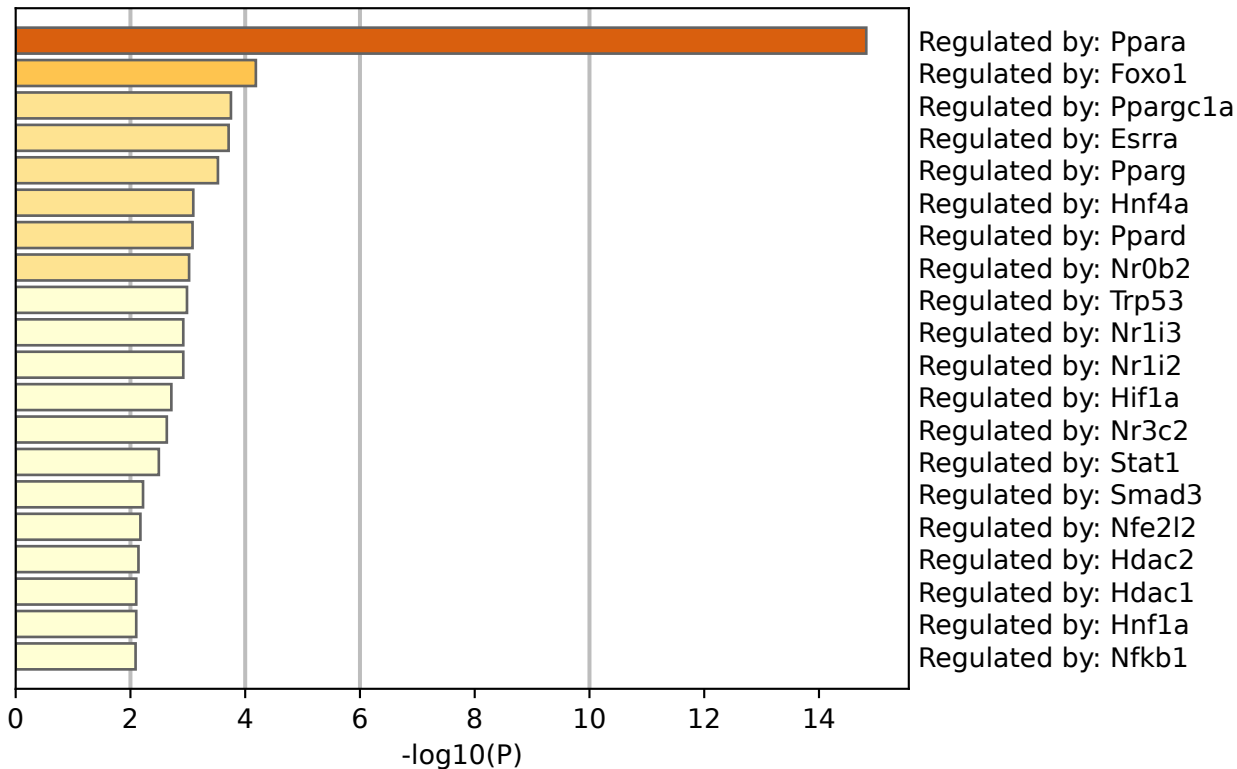

Supplement: Supplementary file 5 — Source data Fig. 3 [file 44319_2026_790_MOESM5_ESM.zip › Figure 3/3E/HeatmapSelectedGO_TRRUST cluster1_liver.pdf]

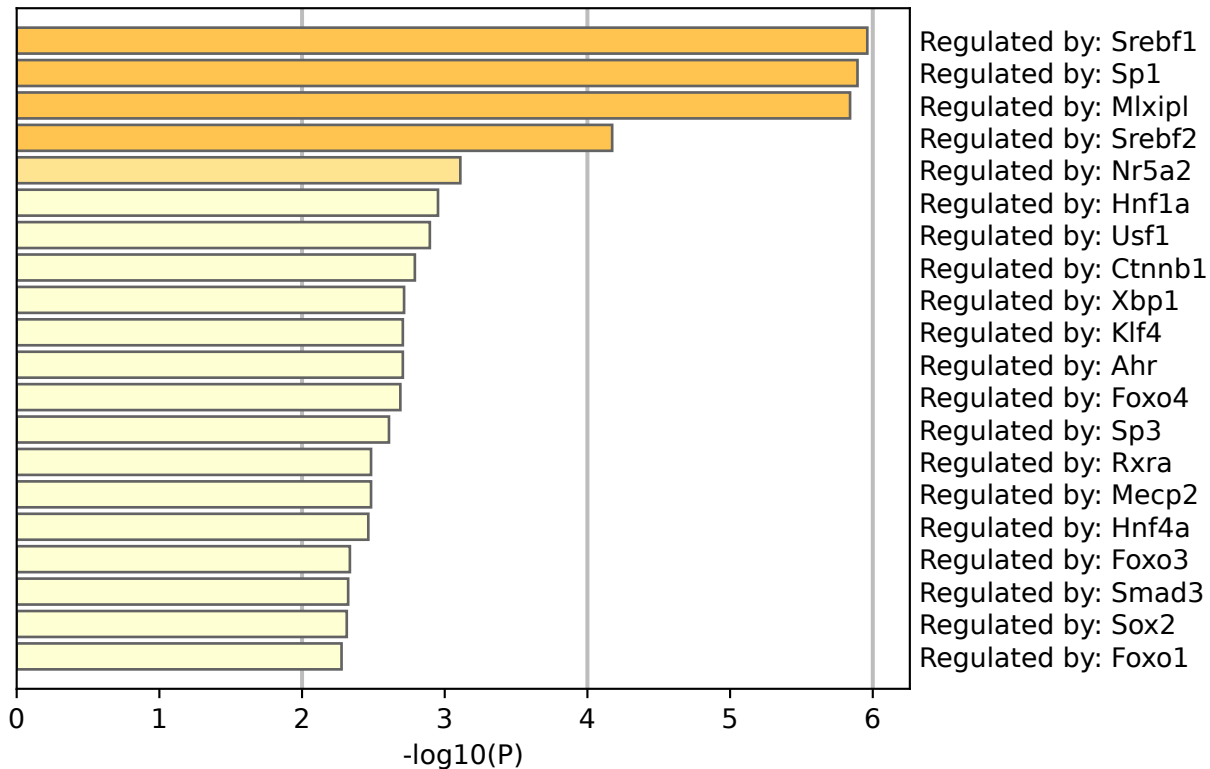

Supplement: Supplementary file 5 — Source data Fig. 3 [file 44319_2026_790_MOESM5_ESM.zip › Figure 3/3E/HeatmapSelectedGO_TRRUST cluster2_liver.pdf]

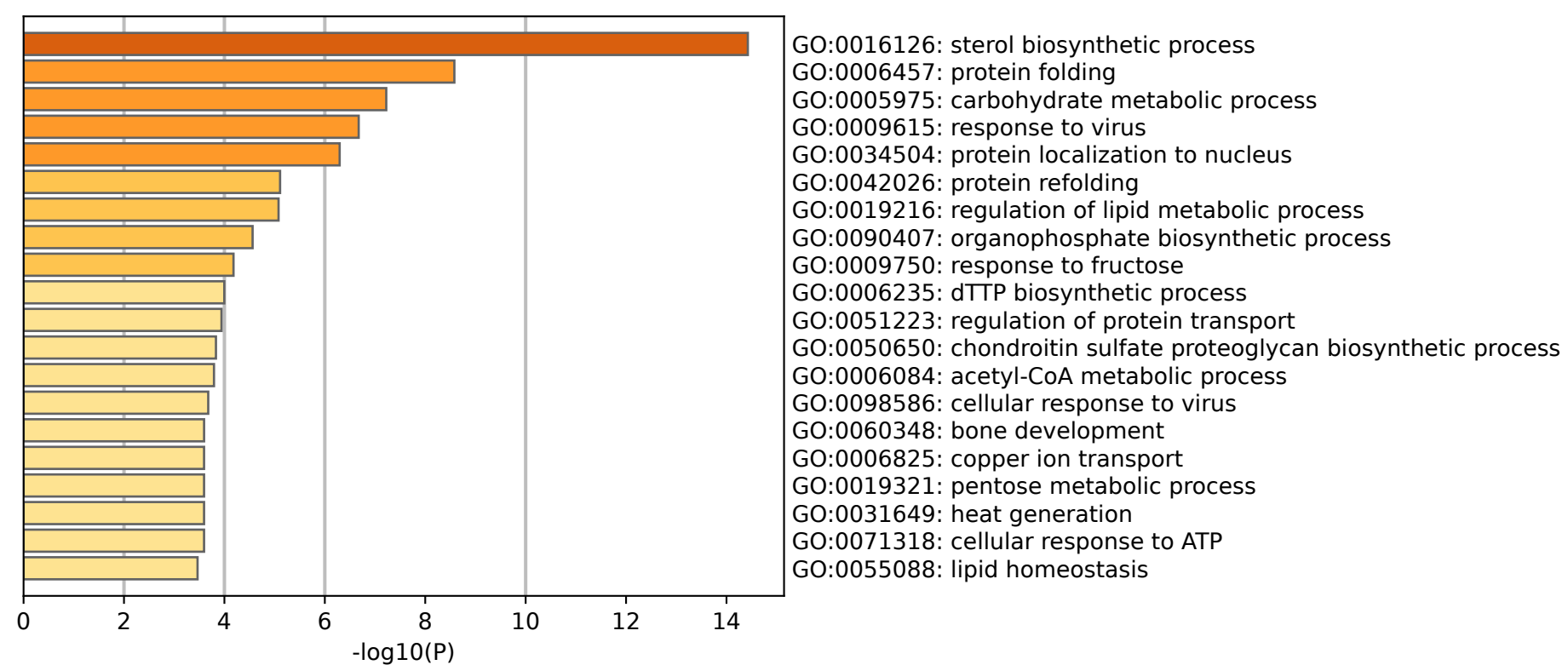

Supplement: Supplementary file 6 — Source data Fig. 4 [file 44319_2026_790_MOESM6_ESM.zip › Figure 4/4G/HeatmapSelectedGO cluster1_scWAT.pdf]

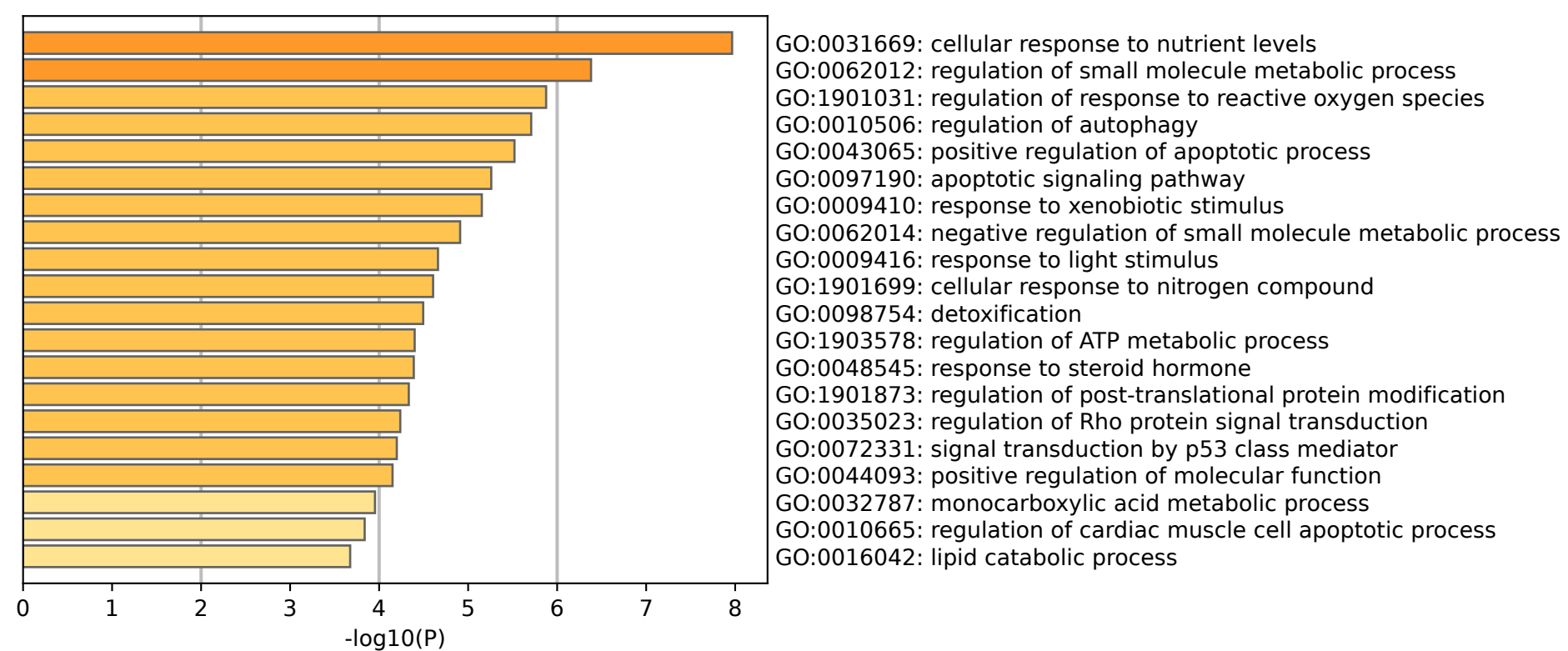

Supplement: Supplementary file 6 — Source data Fig. 4 [file 44319_2026_790_MOESM6_ESM.zip › Figure 4/4G/HeatmapSelectedGO cluster2_scWAT.pdf]

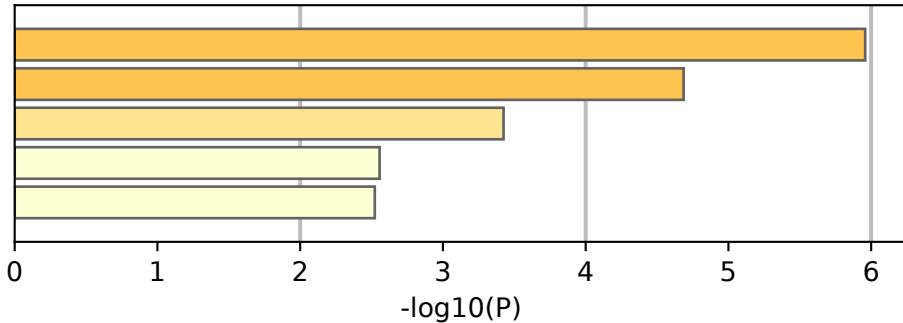

Regulated by: Ppara  
Regulated by: Mecp2  
Regulated by: Nr3c2  
Regulated by: Arntl  
Regulated by: Stat5a

Supplement: Supplementary file 6 — Source data Fig. 4 [file 44319_2026_790_MOESM6_ESM.zip › Figure 4/4H/HeatmapSelectedGO_TRRUST cluster2_scWAT.pdf]

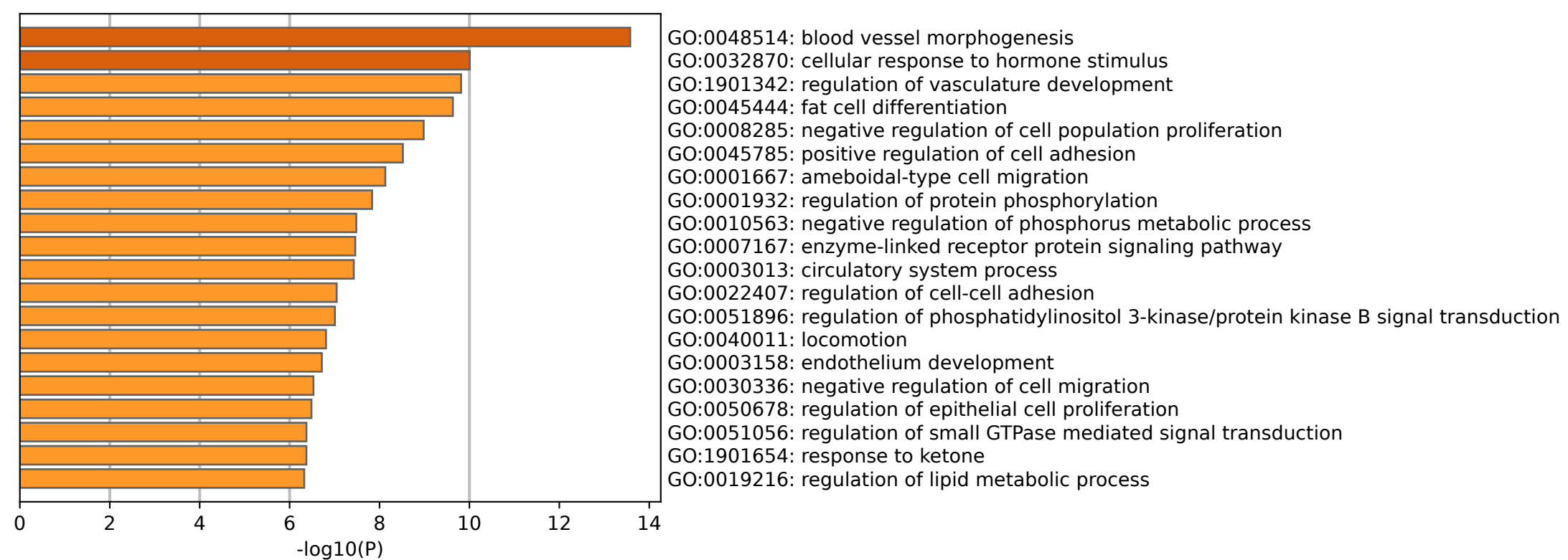

Supplement: Supplementary file 6 — Source data Fig. 4 [file 44319_2026_790_MOESM6_ESM.zip › Figure 4/4K/HeatmapSelectedGO cluster1_epWAT.pdf]

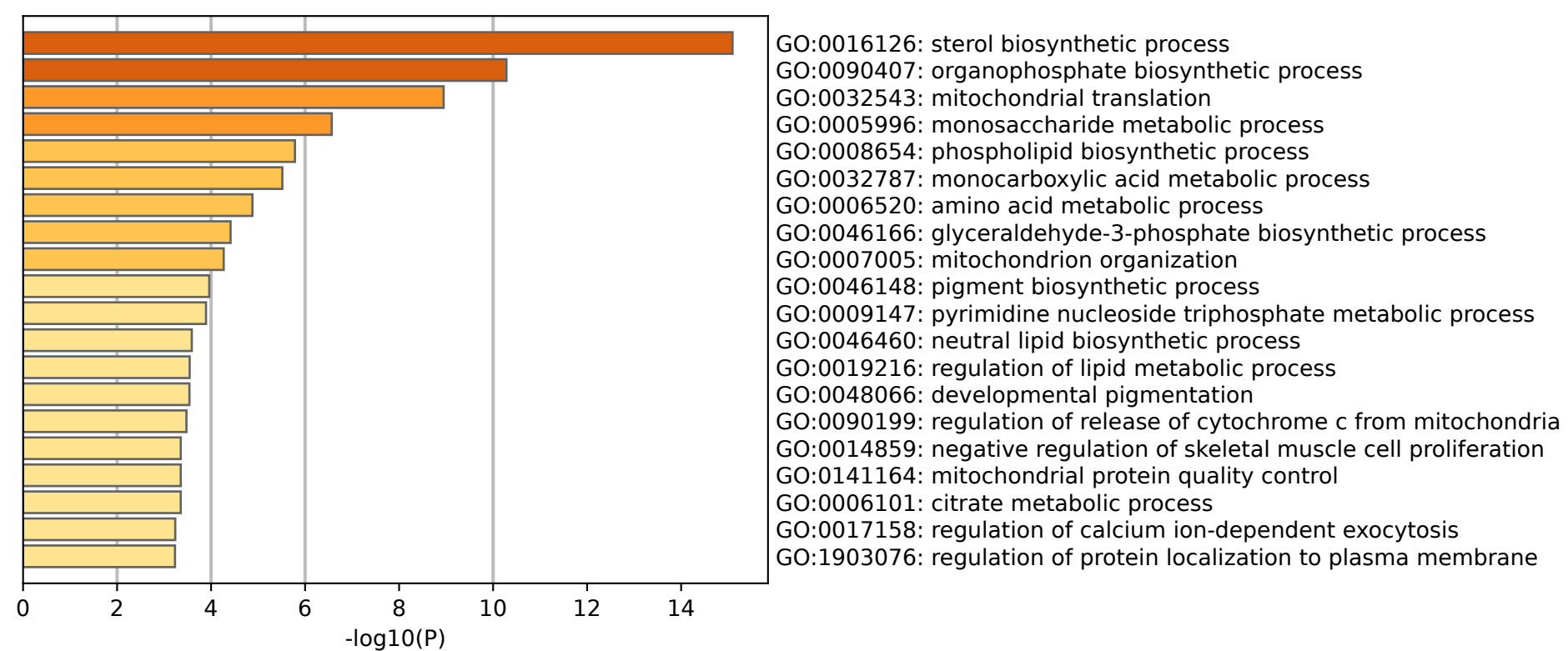

Supplement: Supplementary file 6 — Source data Fig. 4 [file 44319_2026_790_MOESM6_ESM.zip › Figure 4/4K/HeatmapSelectedGO cluster2_epWAT.pdf]

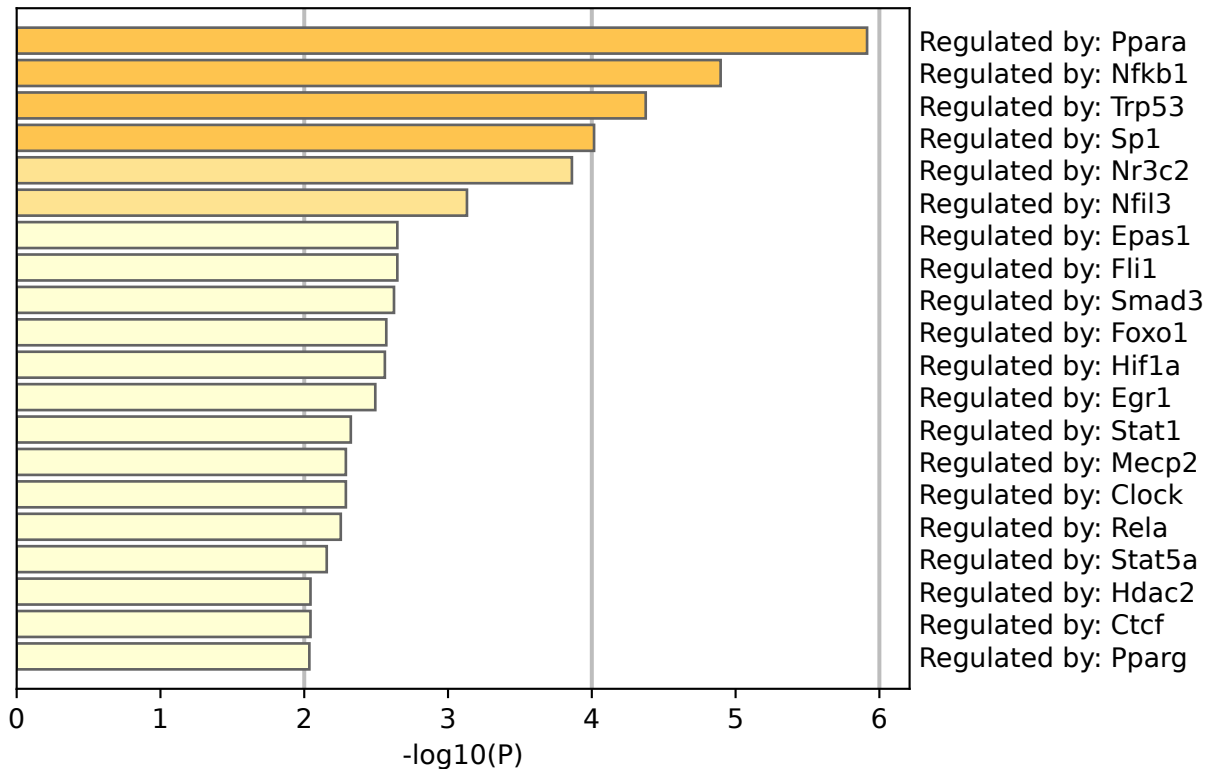

Supplement: Supplementary file 6 — Source data Fig. 4 [file 44319_2026_790_MOESM6_ESM.zip › Figure 4/4L/HeatmapSelectedGO_TRRUST cluster1_epWAT.pdf]

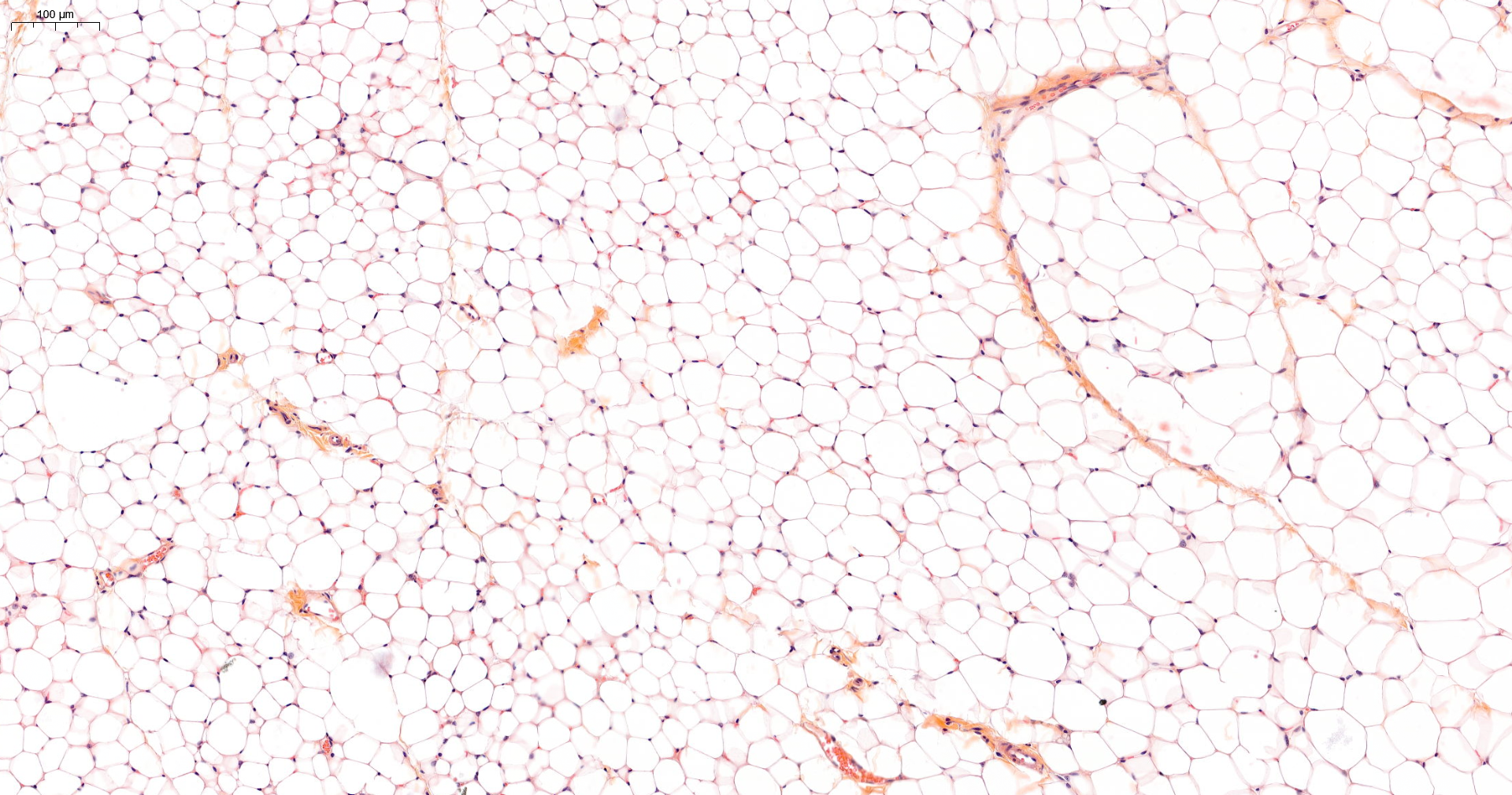

Supplement: Supplementary file 6 — Source data Fig. 4 [file 44319_2026_790_MOESM6_ESM.zip › Figure 4/4M/scWAT FGF21LKO Fasted.tif]

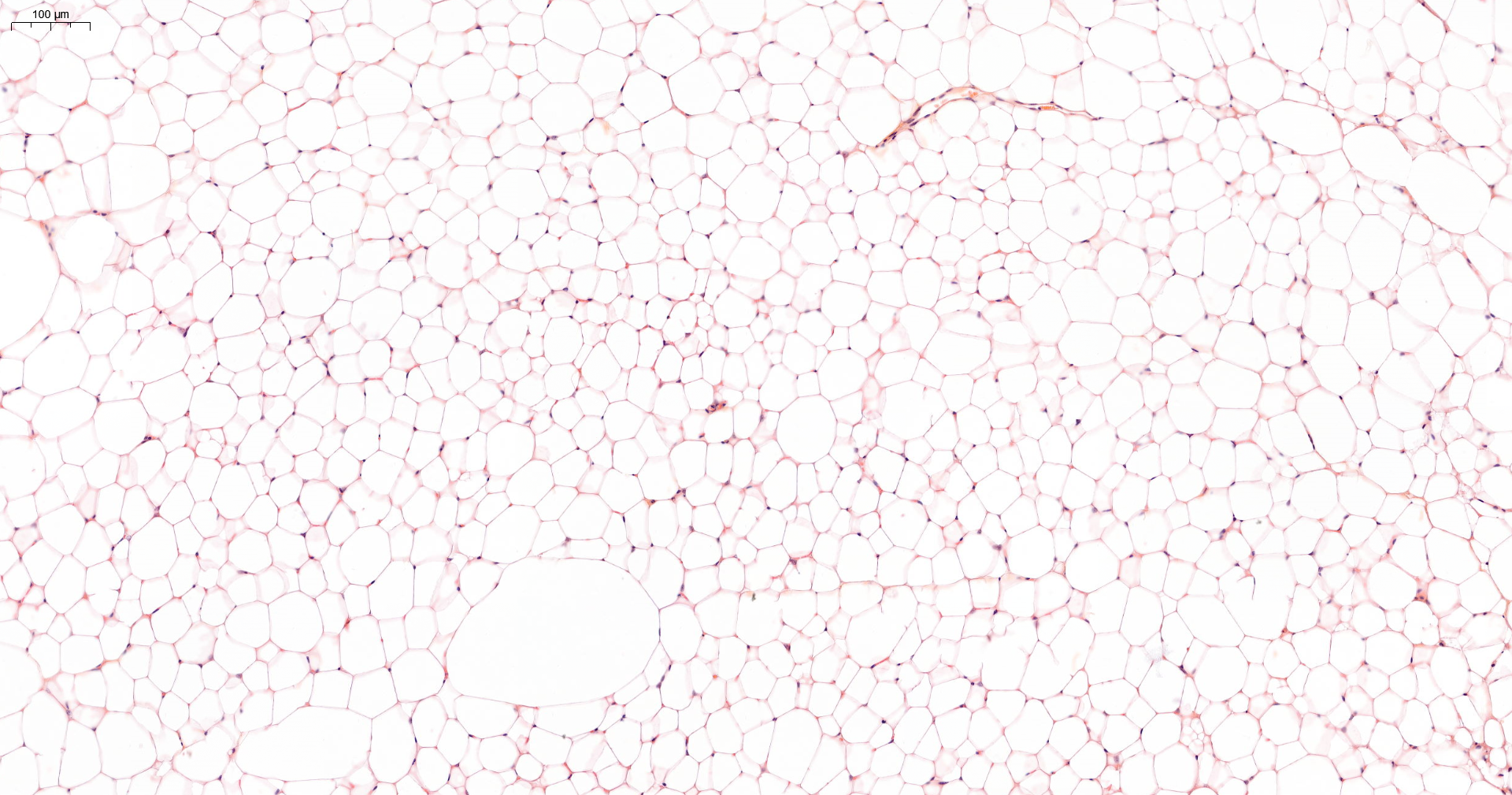

Supplement: Supplementary file 6 — Source data Fig. 4 [file 44319_2026_790_MOESM6_ESM.zip › Figure 4/4M/scWAT FGF21LKO Fed.tif]

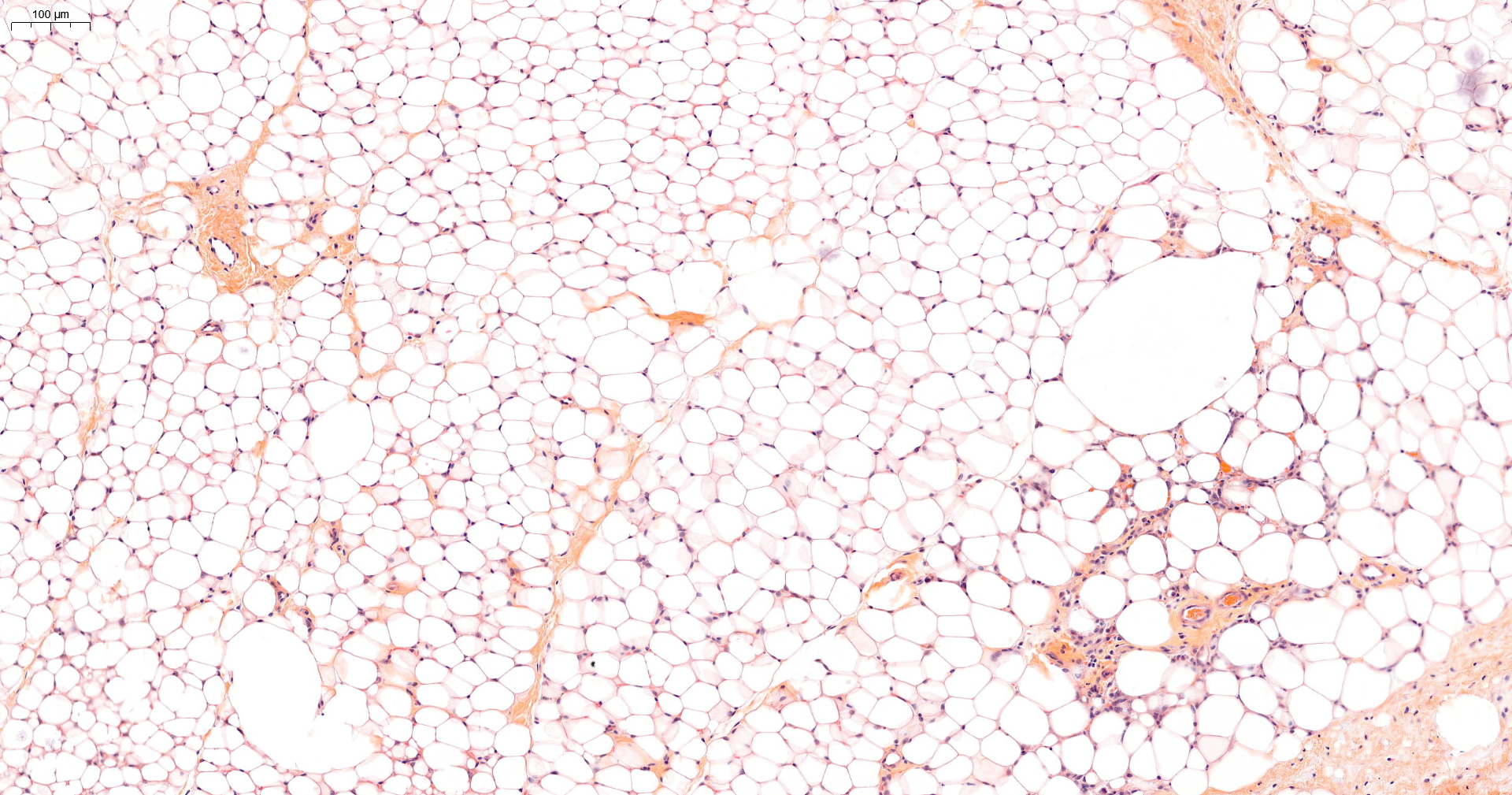

Supplement: Supplementary file 6 — Source data Fig. 4 [file 44319_2026_790_MOESM6_ESM.zip › Figure 4/4M/scWAT FGF21LWT Fasted.tif]

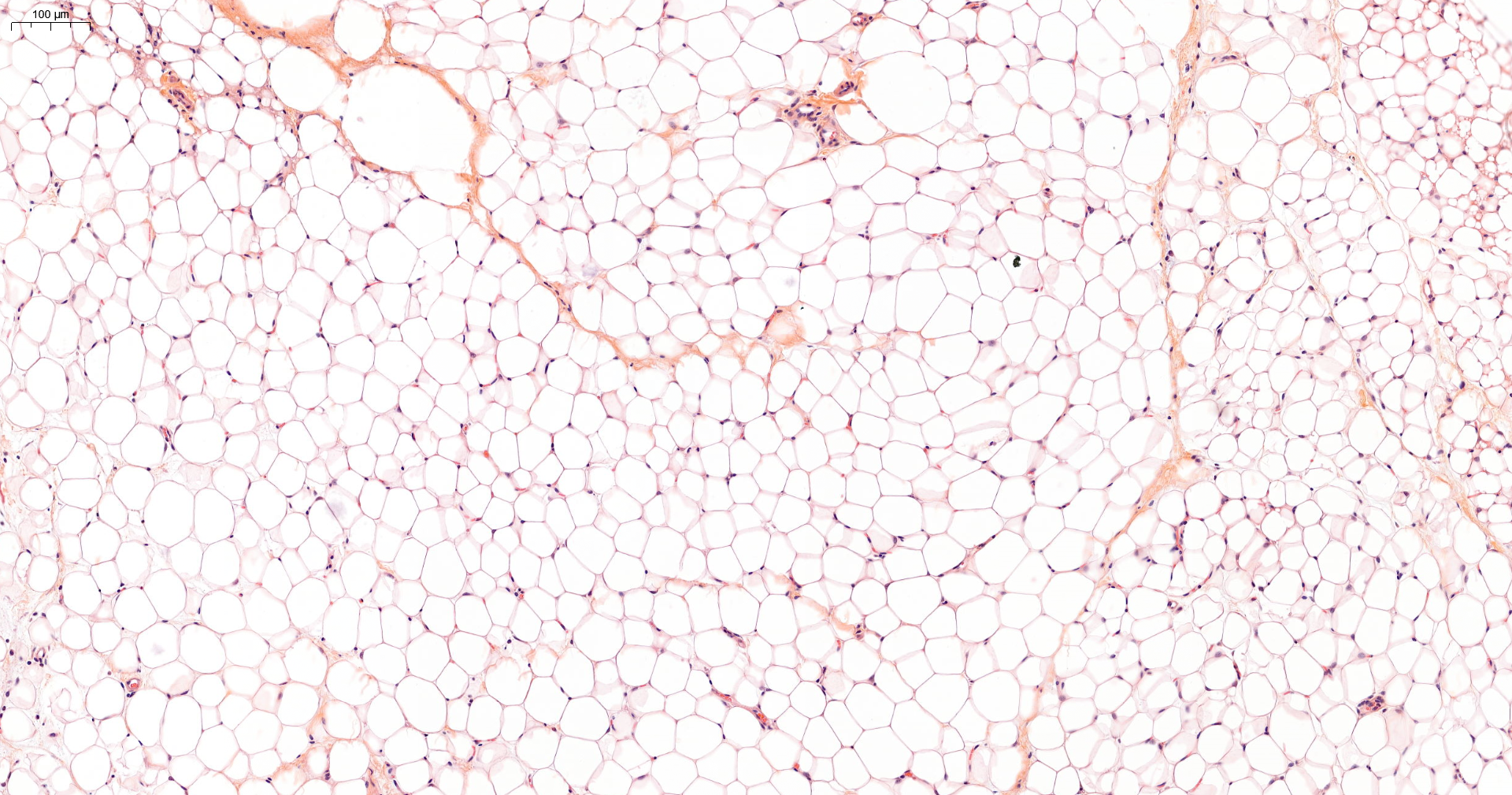

Supplement: Supplementary file 6 — Source data Fig. 4 [file 44319_2026_790_MOESM6_ESM.zip › Figure 4/4M/scWATsc FGF21LWT Fed.tif]

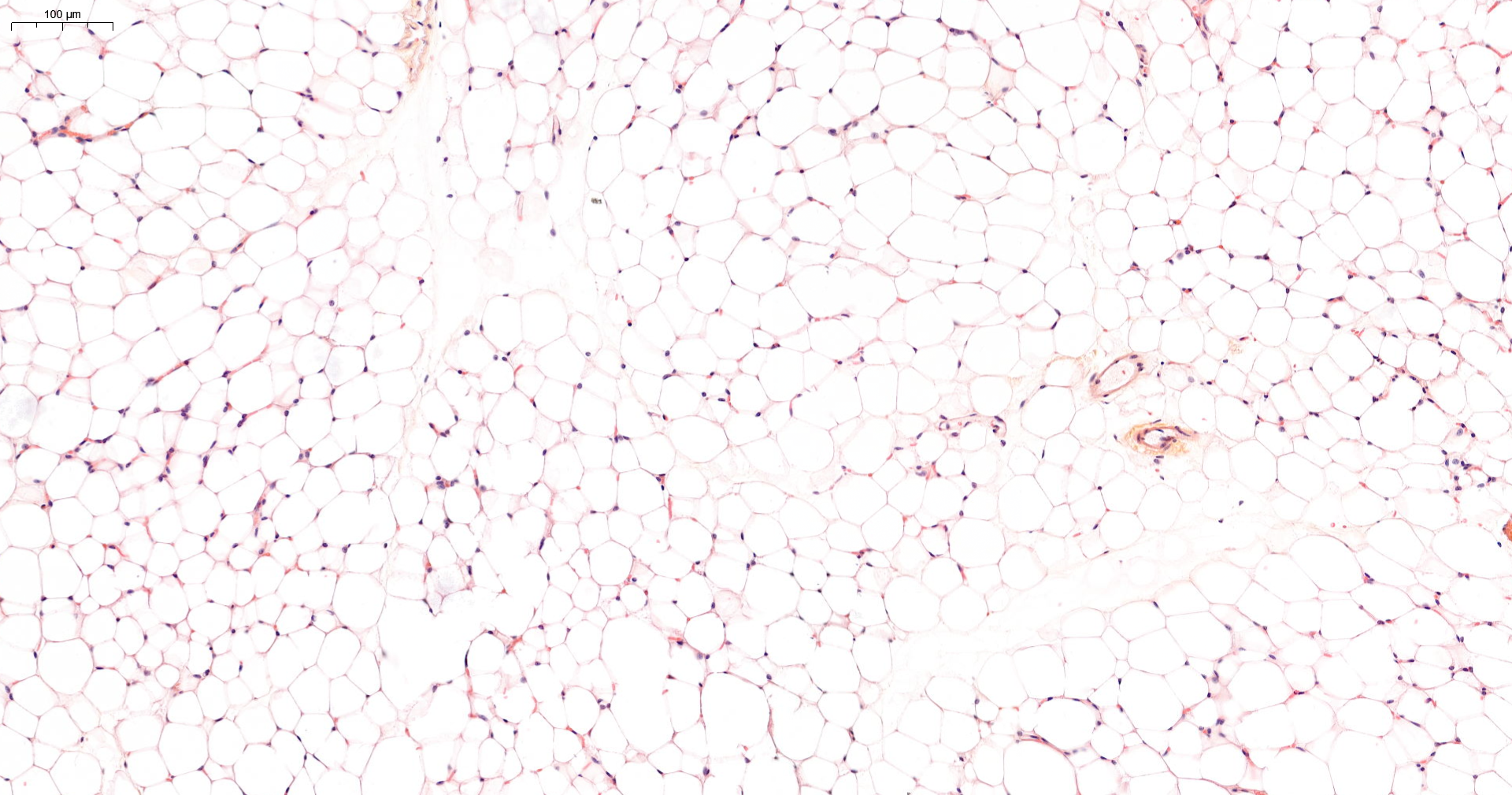

Supplement: Supplementary file 6 — Source data Fig. 4 [file 44319_2026_790_MOESM6_ESM.zip › Figure 4/4N/epWAT FGF21LKO Fasted.tif]

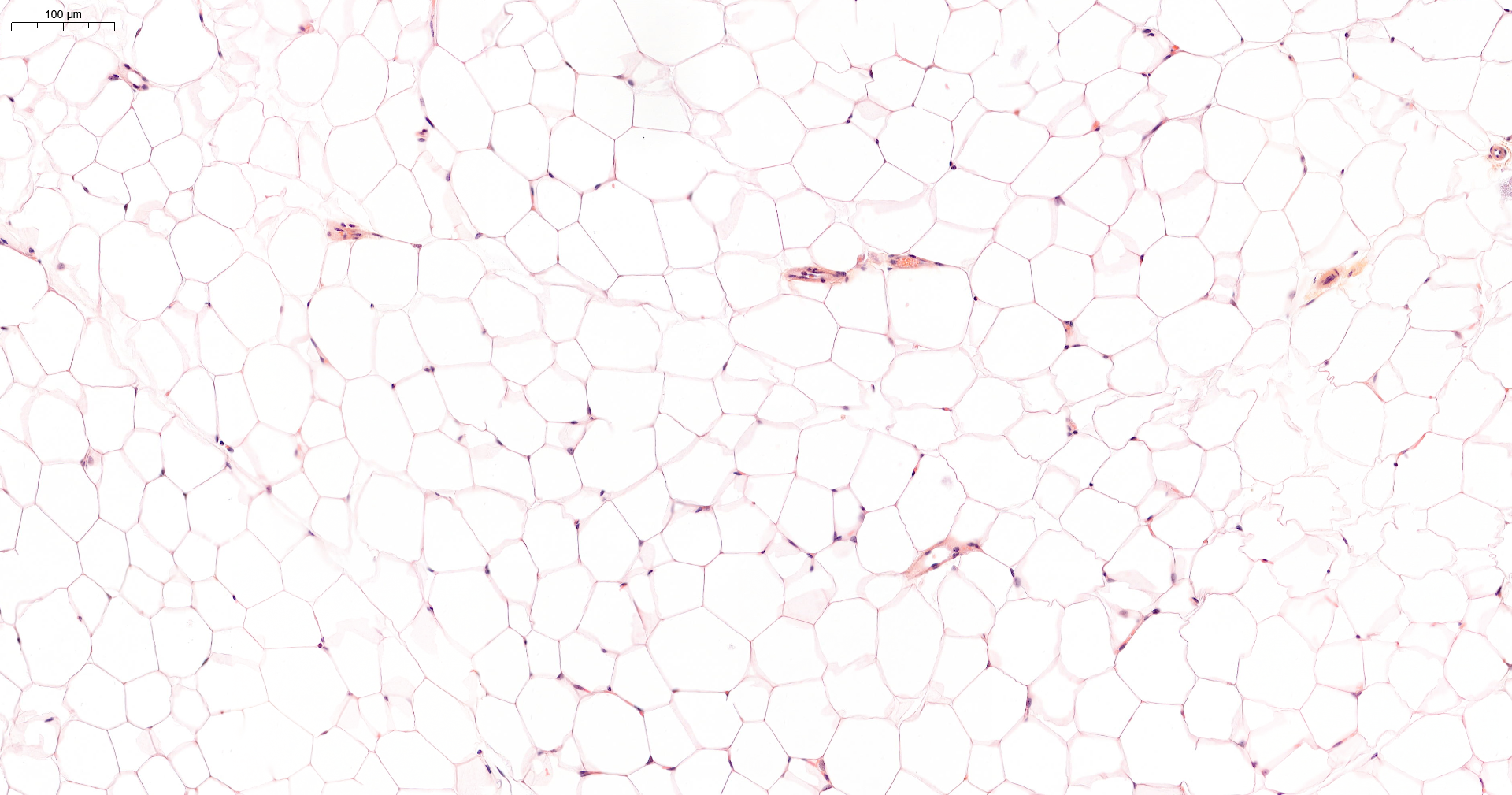

Supplement: Supplementary file 6 — Source data Fig. 4 [file 44319_2026_790_MOESM6_ESM.zip › Figure 4/4N/epWAT FGF21LKO Fed.tif]

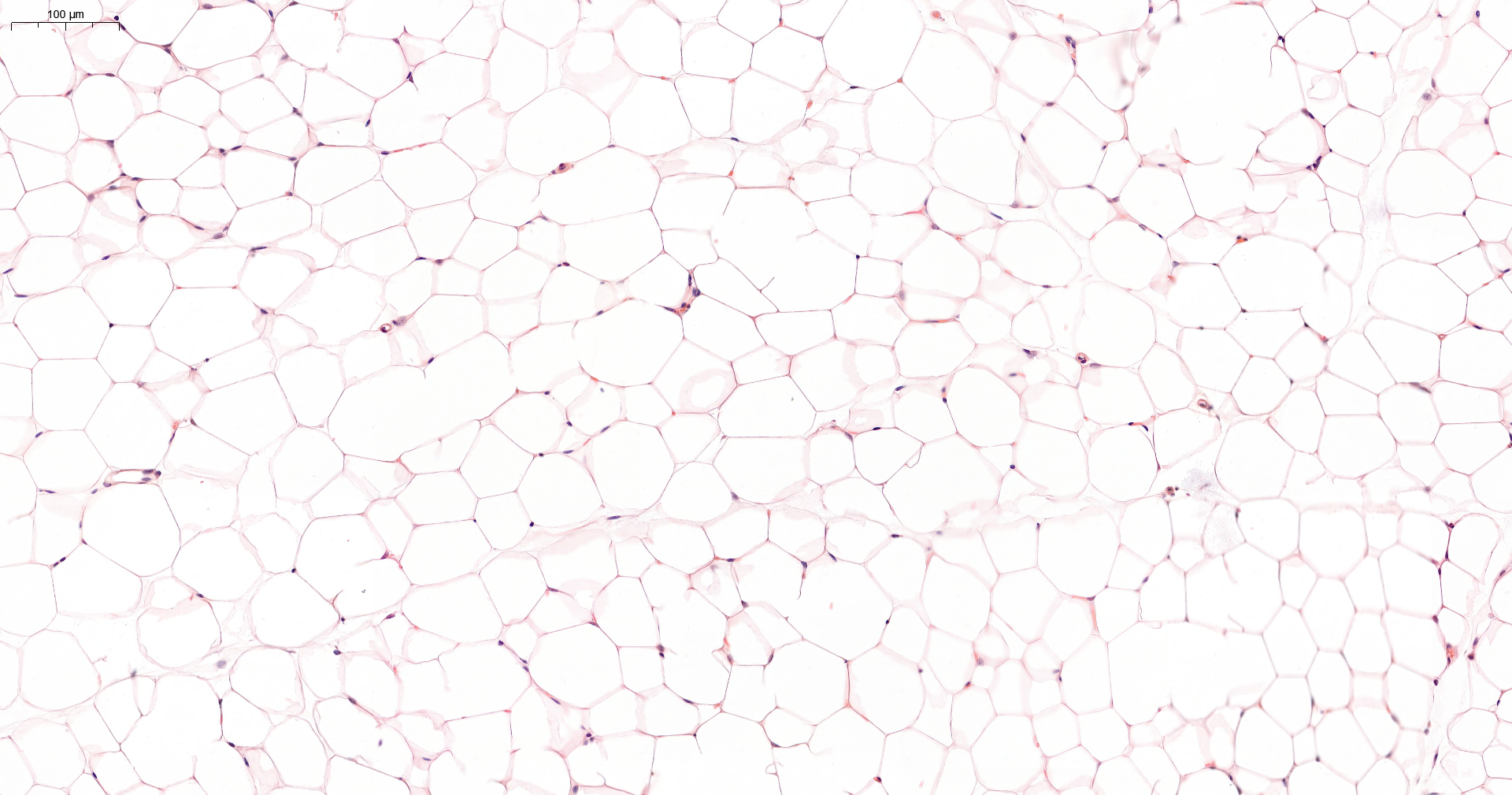

Supplement: Supplementary file 6 — Source data Fig. 4 [file 44319_2026_790_MOESM6_ESM.zip › Figure 4/4N/epWAT FGF21LWT Fasted.tif]

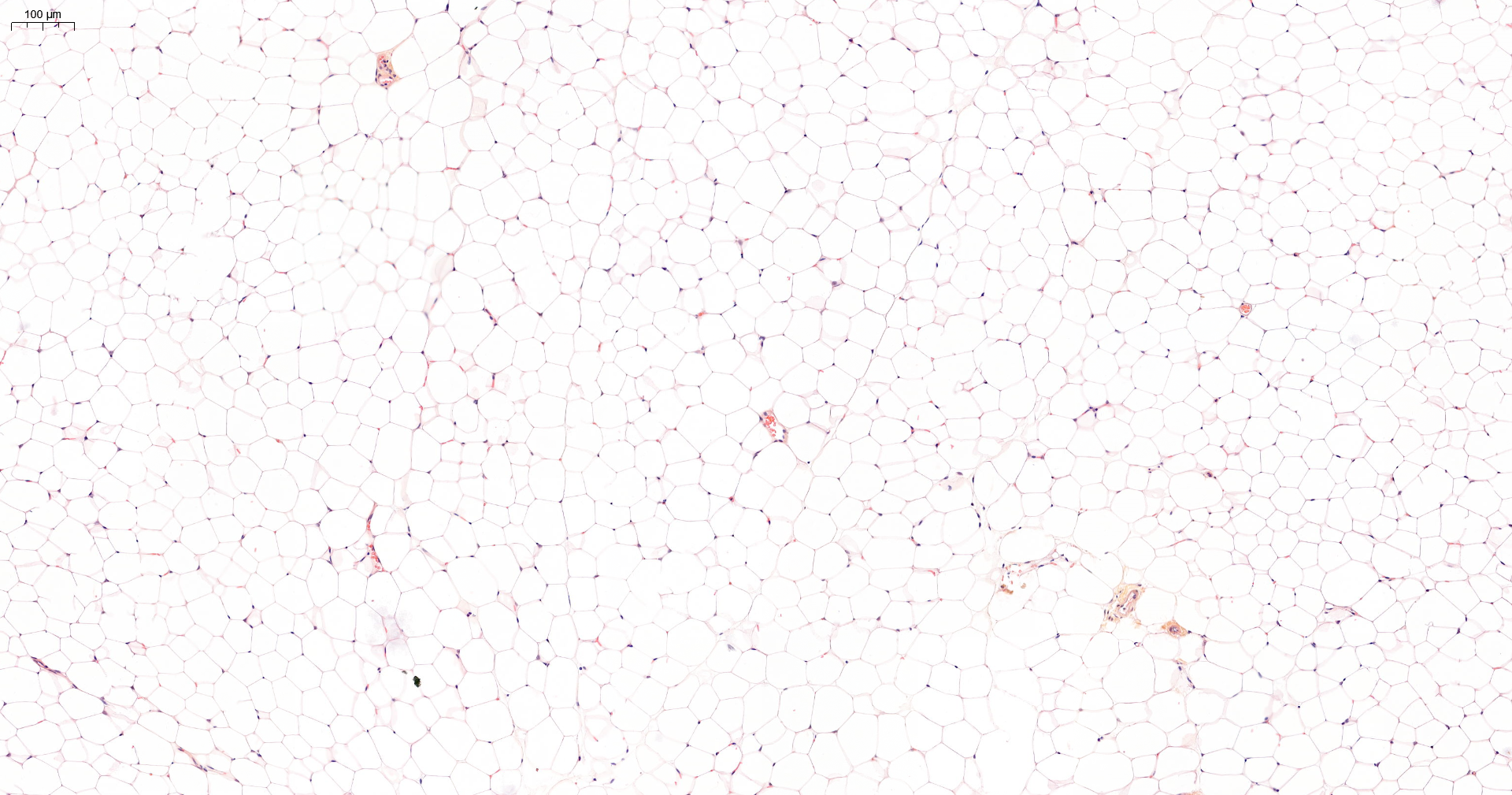

Supplement: Supplementary file 6 — Source data Fig. 4 [file 44319_2026_790_MOESM6_ESM.zip › Figure 4/4N/epWAT FGF21LWT Fed.tif]

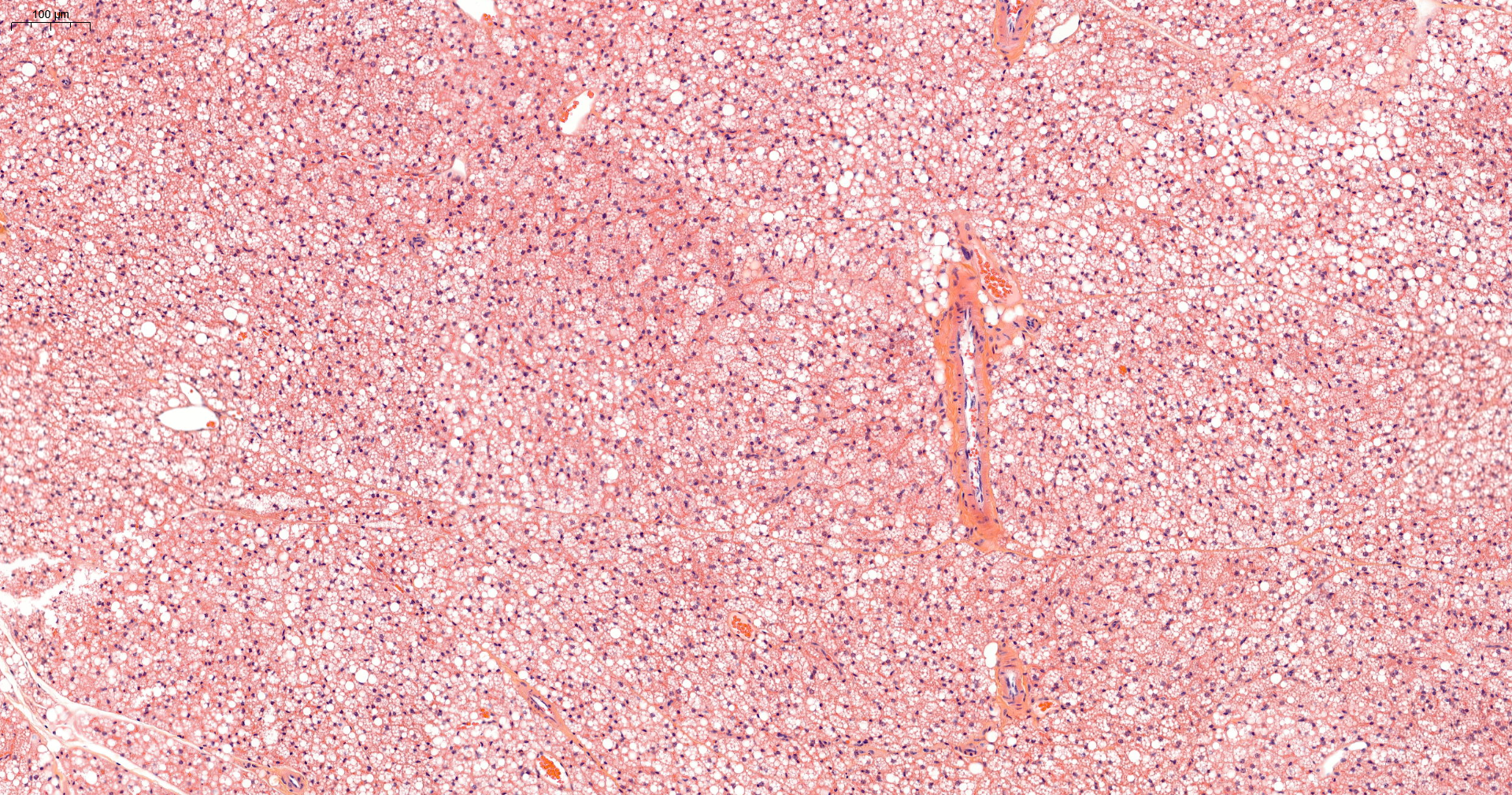

Supplement: Supplementary file 7 — Source data Fig. 5 [file 44319_2026_790_MOESM7_ESM.zip › Figure 5/5B/BAT FGF21LKO Fasted.tif]

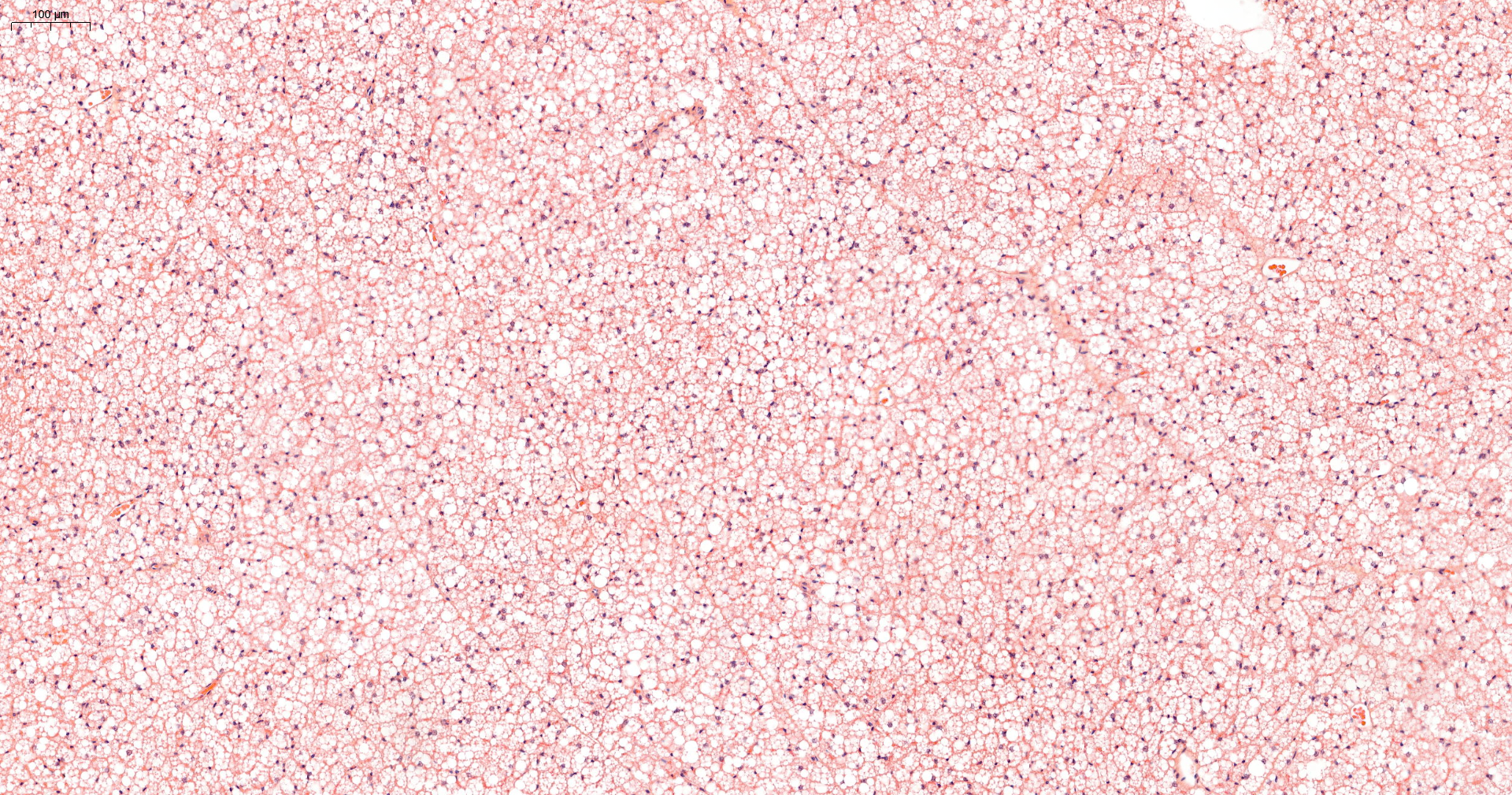

Supplement: Supplementary file 7 — Source data Fig. 5 [file 44319_2026_790_MOESM7_ESM.zip › Figure 5/5B/BAT FGF21LKO Fed.tif]

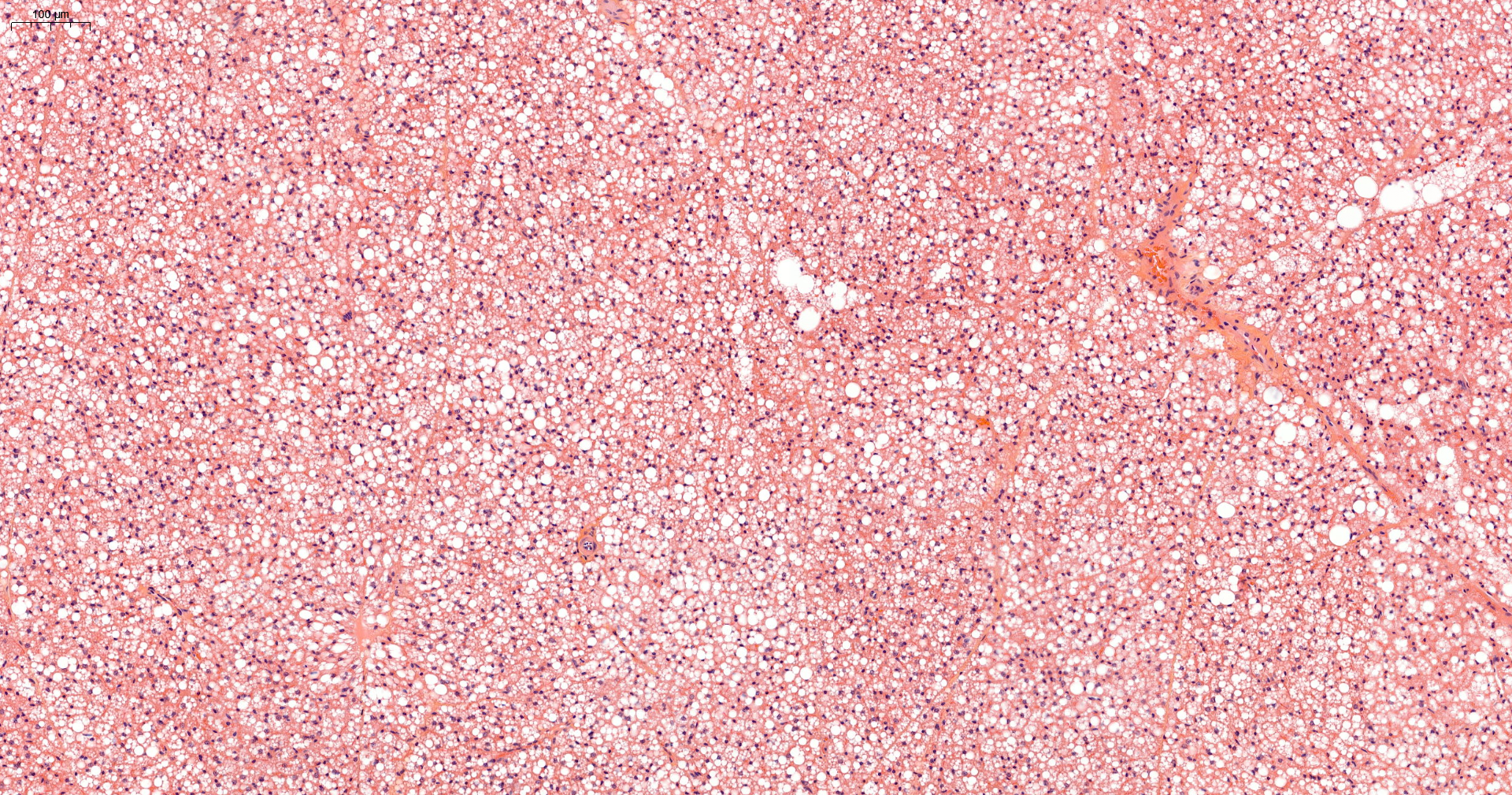

Supplement: Supplementary file 7 — Source data Fig. 5 [file 44319_2026_790_MOESM7_ESM.zip › Figure 5/5B/BAT FGF21LWT Fasted.tif]

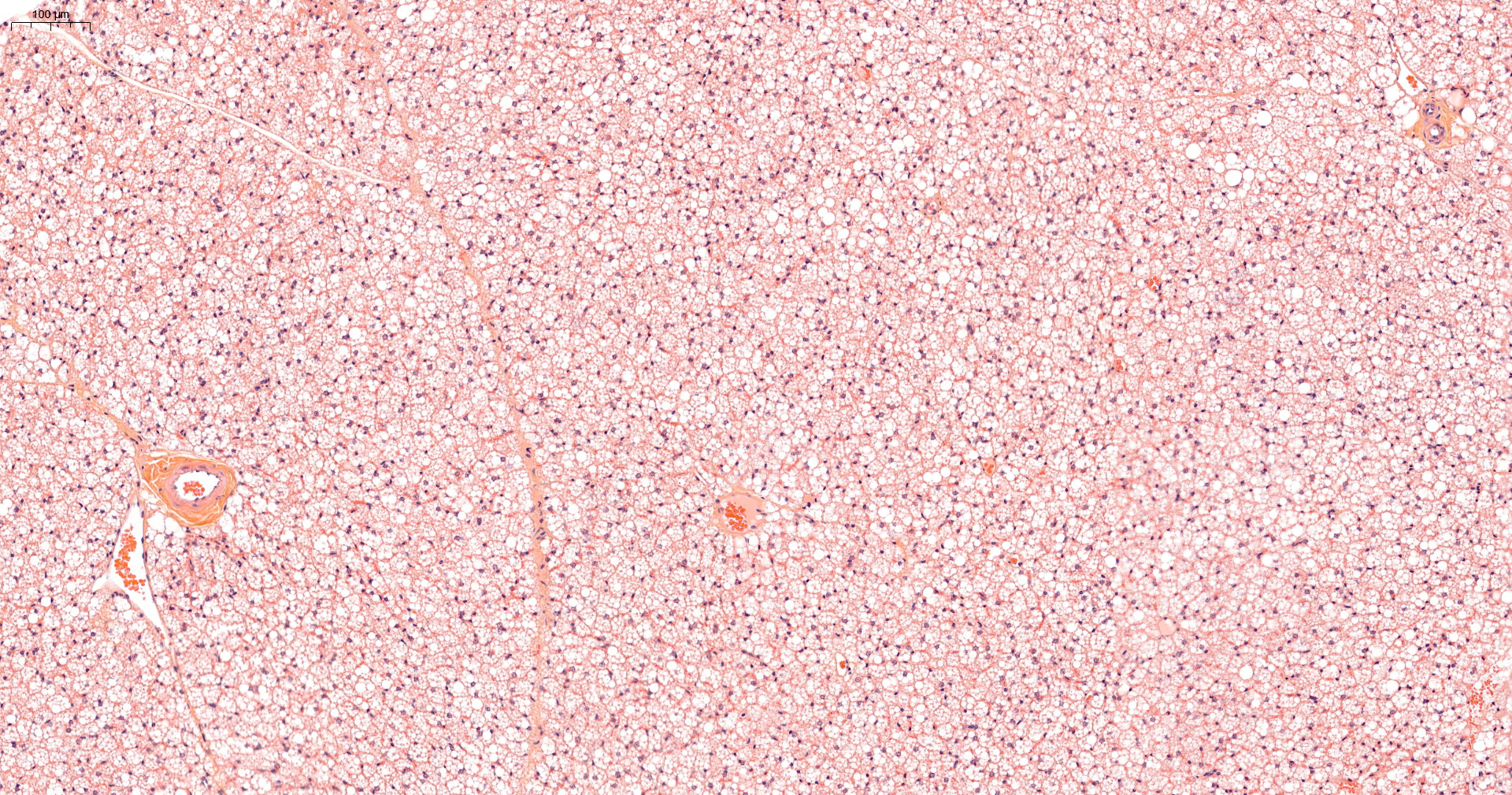

Supplement: Supplementary file 7 — Source data Fig. 5 [file 44319_2026_790_MOESM7_ESM.zip › Figure 5/5B/BAT FGF21LWT Fed.tif]

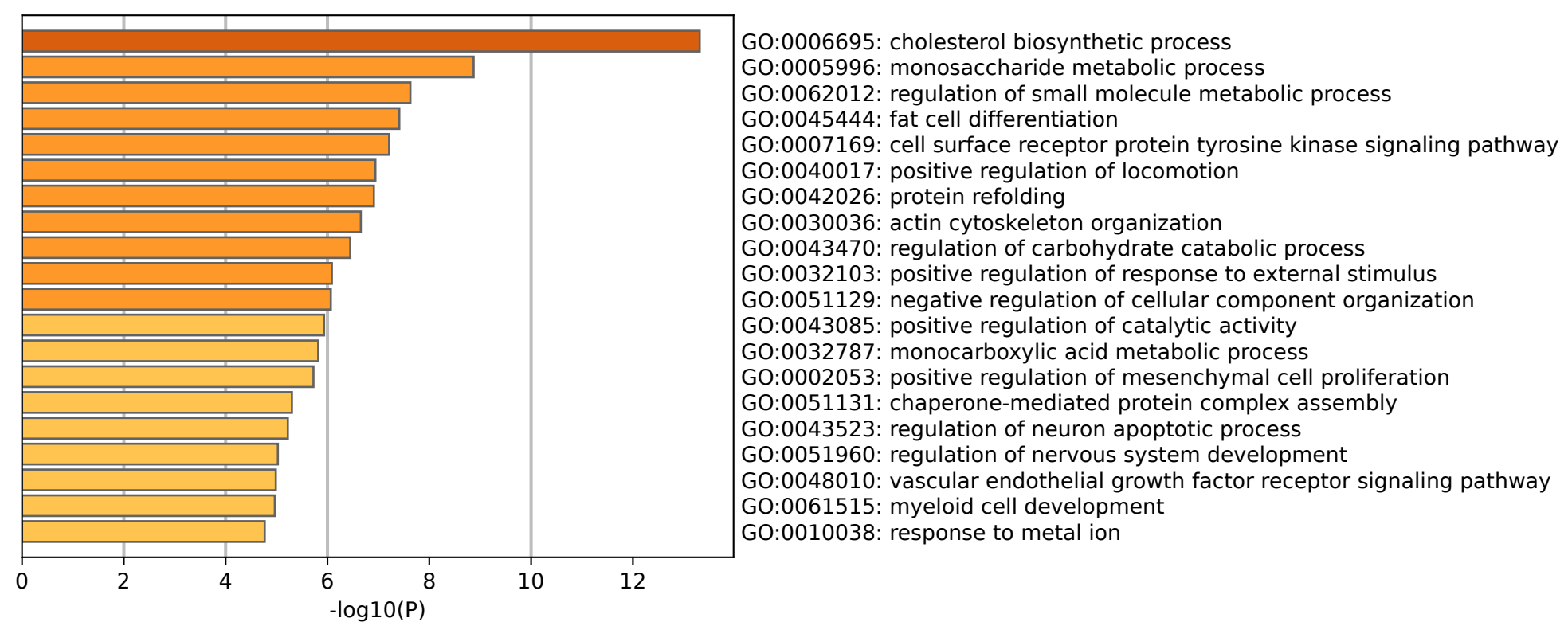

Supplement: Supplementary file 7 — Source data Fig. 5 [file 44319_2026_790_MOESM7_ESM.zip › Figure 5/5E/HeatmapSelectedGO cluster1_BAT.pdf]

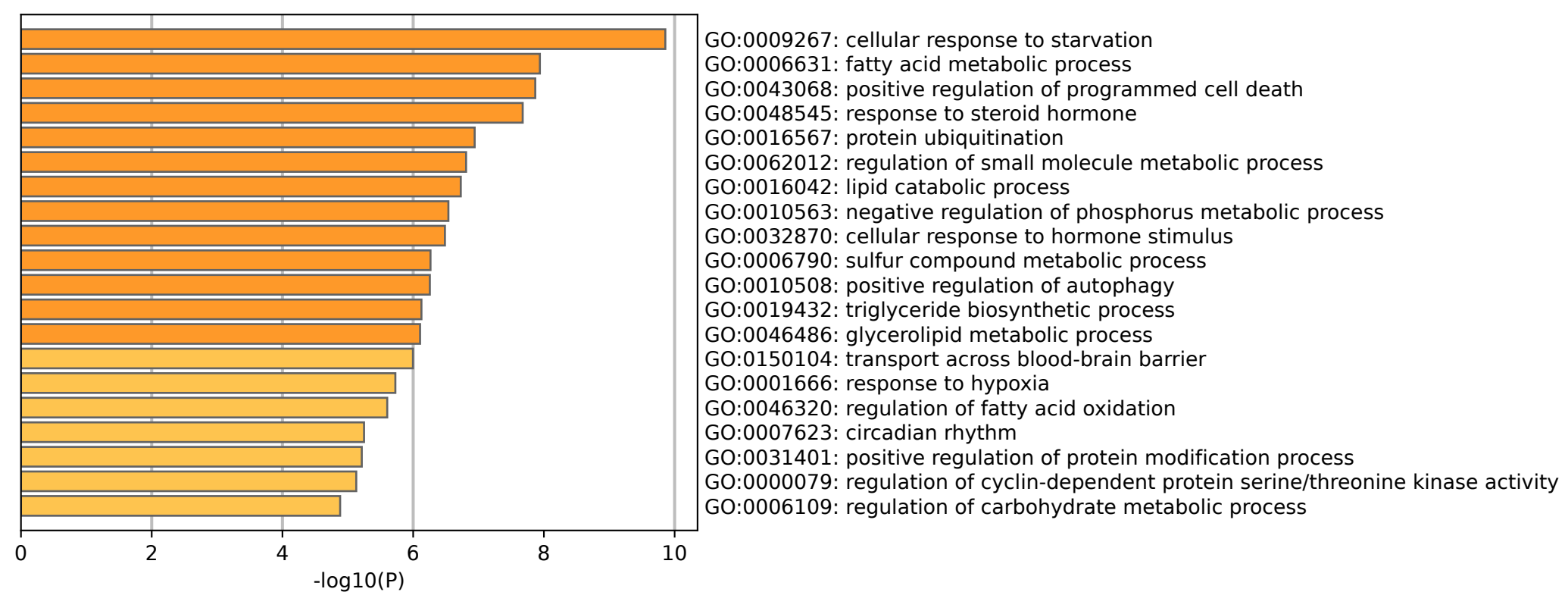

Supplement: Supplementary file 7 — Source data Fig. 5 [file 44319_2026_790_MOESM7_ESM.zip › Figure 5/5E/HeatmapSelectedGO cluster2_BAT.pdf]

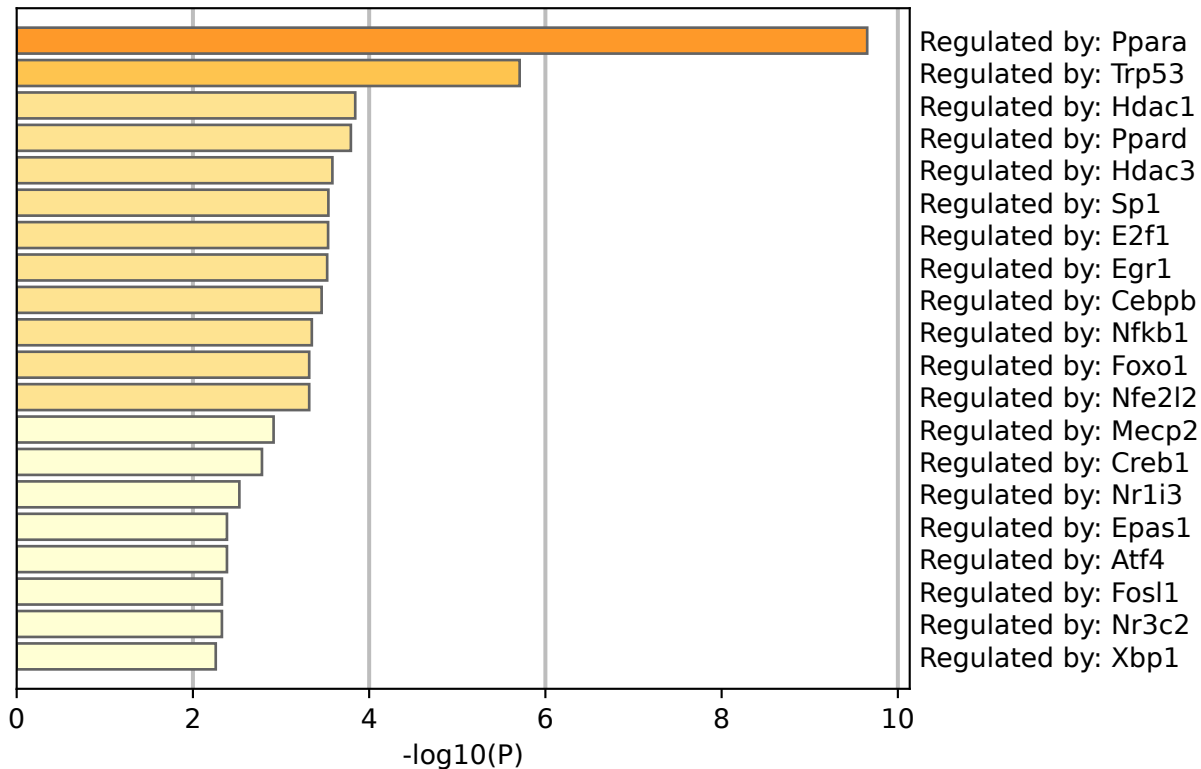

Supplement: Supplementary file 7 — Source data Fig. 5 [file 44319_2026_790_MOESM7_ESM.zip › Figure 5/5F/HeatmapSelectedGO_TRRUST cluster2_BAT.pdf]
