# Supplementary material for: Hepatic FGF21 is not required for fasting metabolism but guides protein appetite post energy depletion
Source: EMBO Rep. 2026 Apr 27;27(12):3189–213. doi: 10.1038/s44319-026-00790-9 (PMC13303862; doi:10.1038/s44319-026-00790-9)
Supplement: Supplementary file 6 — Source data Fig. 4 [file 44319_2026_790_MOESM6_ESM.zip › Figure 4/4H/HeatmapSelectedGO_TRRUST cluster1_scWAT.pdf]

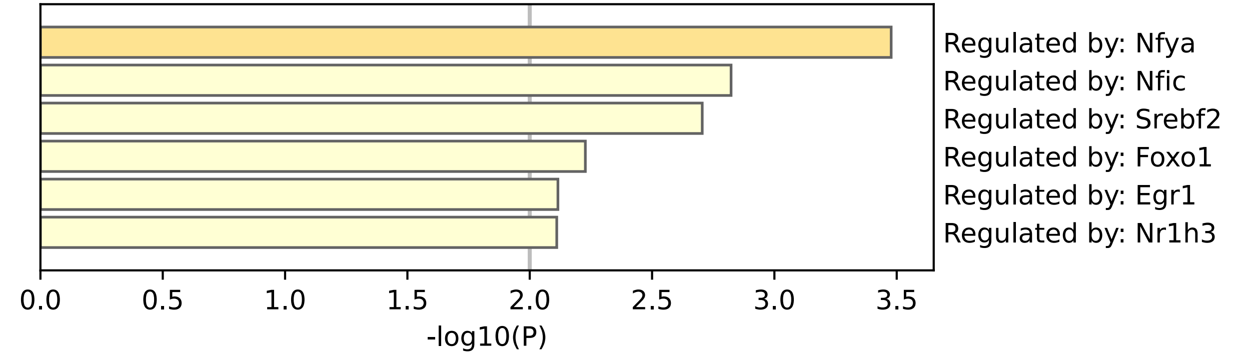
Regulated by: Nfya
Regulated by: Nfic
Regulated by: Srebf2
Regulated by: Foxo1
Regulated by: Egr1
Regulated by: Nr1h3
0.0
0.5
1.0
1.5
2.0
2.5
3.0
3.5
-log10(P)
