# Supplementary material for: Hepatic FGF21 is not required for fasting metabolism but guides protein appetite post energy depletion
Source: EMBO Rep. 2026 Apr 27;27(12):3189–213. doi: 10.1038/s44319-026-00790-9 (PMC13303862; doi:10.1038/s44319-026-00790-9)
Supplement: Supplementary file 7 — Source data Fig. 5 [file 44319_2026_790_MOESM7_ESM.zip › Figure 5/5F/HeatmapSelectedGO_TRRUST cluster1_BAT.pdf]

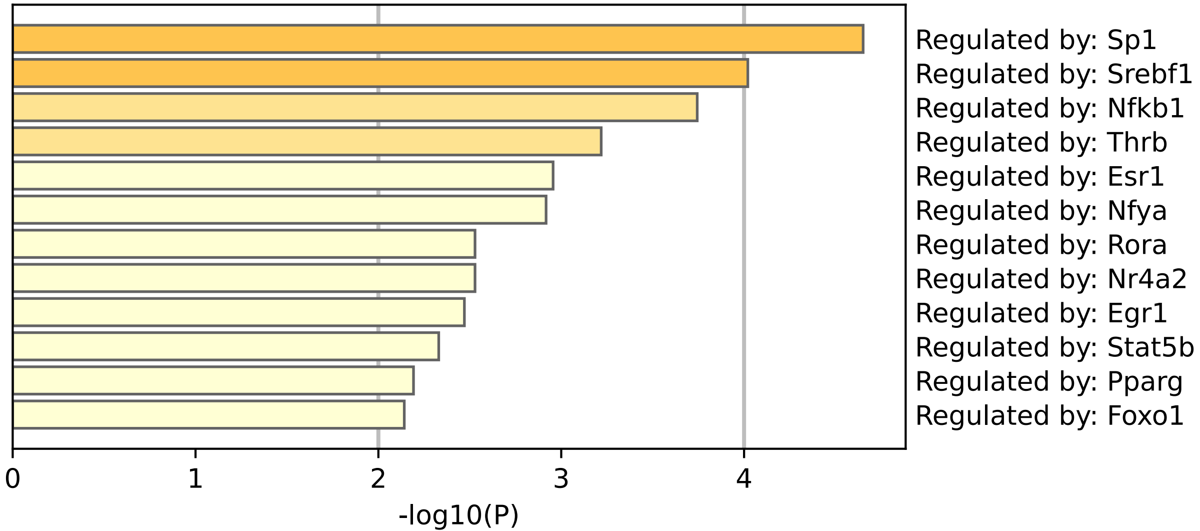

Regulated by: Sp1
Regulated by: Srebf1
Regulated by: Nfkb1
Regulated by: Thrb
Regulated by: Esr1
Regulated by: Nfya
Regulated by: Rora
Regulated by: Nr4a2
Regulated by: Egr1
Regulated by: Stat5b
Regulated by: Pparg
Regulated by: Foxo1
0
1
2
3
4
-log10(P)
