# Supplementary material for: Hepatic FGF21 is not required for fasting metabolism but guides protein appetite post energy depletion
Source: EMBO Rep. 2026 Apr 27;27(12):3189–213. doi: 10.1038/s44319-026-00790-9 (PMC13303862; doi:10.1038/s44319-026-00790-9)
Supplement: Supplementary file 9 — Expanded View Figures [file 44319_2026_790_MOESM9_ESM.pdf]

Expanded View Figures

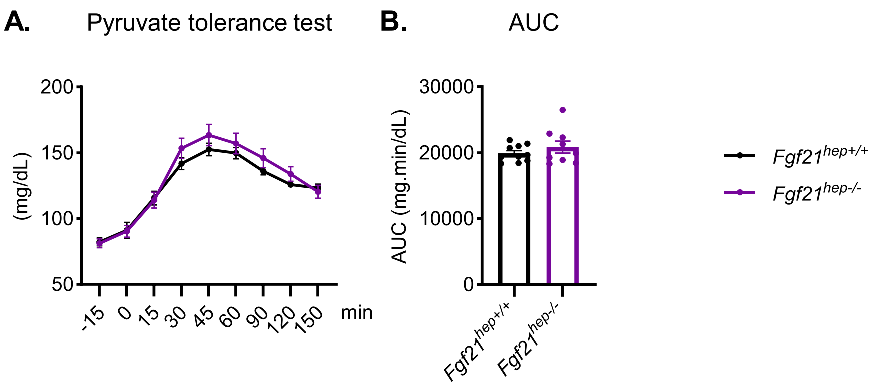

Figure EV1. Hepatocyte-specific deletion of *Fgf21* does not affect glucose production during the pyruvate tolerance test.

Related to Fig. 2. (**A**) Glycemia of pyruvate tolerance test (PTT) ($n = 9$–10 mice per group, biological replicates, Student's *t*-test). (**B**) Area under the curve (AUC) for each mouse representing PTT results ($n = 9$–10 mice per group, biological replicates, Student's *t*-test). Data information: All data were presented as mean ± SEM.

© The Author(s)

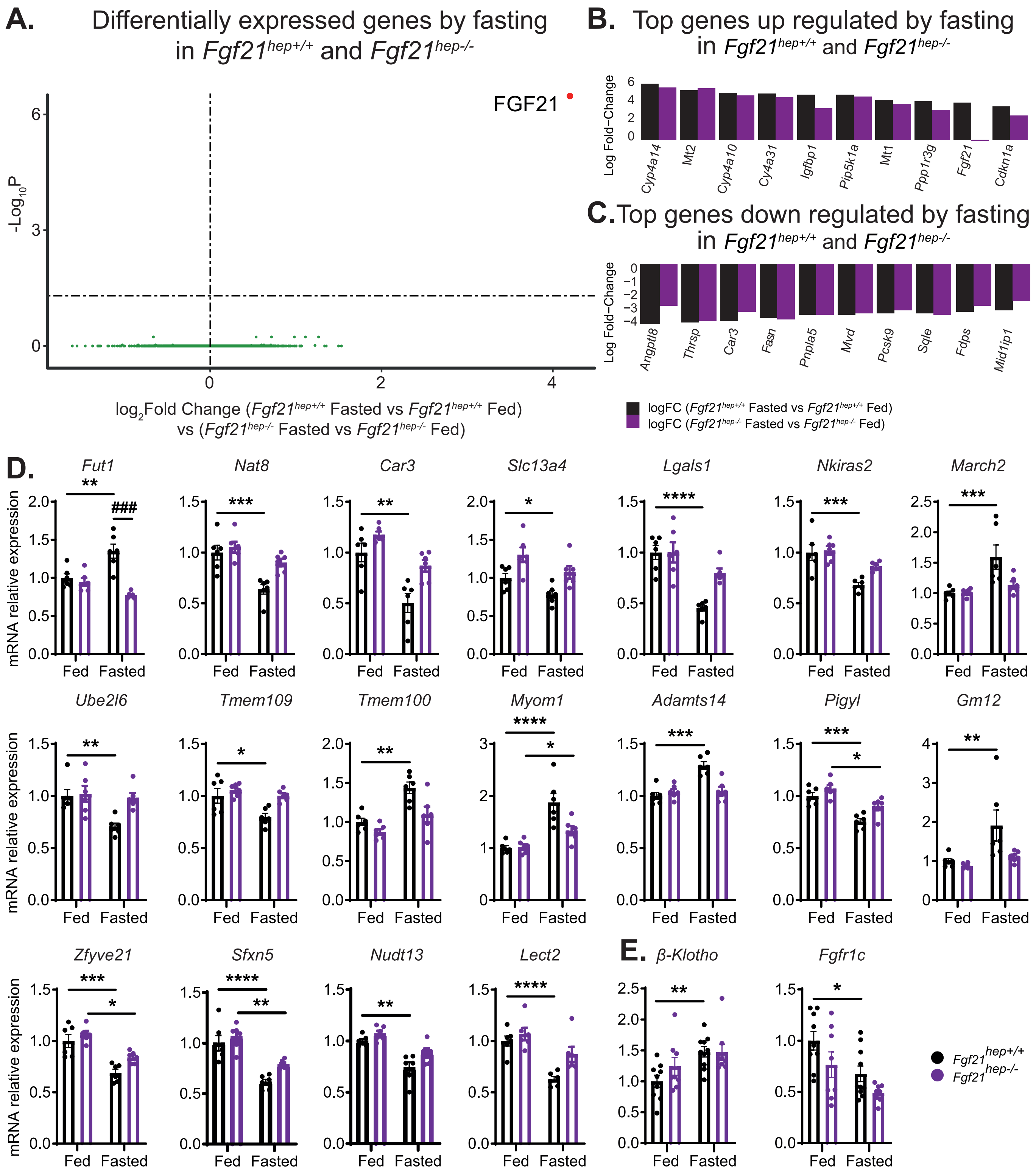
A. Differentially expressed genes by fasting in Fgf21hep+/+ and Fgf21hep-/-
FGF21
-Log10P
log2Fold Change (Fgf21hep+/+ Fasted vs Fgf21hep+/+ Fed) vs (Fgf21hep-/- Fasted vs Fgf21hep-/- Fed)
B. Top genes up regulated by fasting in Fgf21hep+/+ and Fgf21hep-/-
Log Fold−Change
Cyp4a14
Mt2
Cyp4a10
Cy4a31
Igfbp1
Pip5k1a
Mt1
Ppp1r3g
Fgf21
Cdkn1a
C. Top genes down regulated by fasting in Fgf21hep+/+ and Fgf21hep-/-
Log Fold−Change
Angptl8
Thrsp
Car3
Fasn
Pnpla5
Mvd
Pcsk9
Sqle
Fdps
Mid1ip1
logFC (Fgf21hep+/+ Fasted vs Fgf21hep+/+ Fed)
logFC (Fgf21hep-/- Fasted vs Fgf21hep-/- Fed)
D.
mRNA relative expression
Fut1
Nat8
Car3
Slc13a4
Lgals1
Nkiras2
March2
Ube2l6
Tmem109
Tmem100
Myom1
Adamts14
Pigyl
Gm12
Zfyve21
Sfxn5
Nudt13
Lect2
Fed
Fasted
E. β-Klotho
Fgfr1c
Fgf21hep+/+
Fgf21hep-/-

© The Author(s)

Figure EV2. Hepatocyte-specific deletion of *Fgf21* affects the expression of only a few genes in the liver during fasting.

Related to Fig. 3. *Fgf21* liver floxed (*Fgf21*$^{hep+/+}$) or *Fgf21* liver knockout (*Fgf21*$^{hep-/-}$) mice were fed *ad libitum* or fasted for 20 h. (**A**) Volcano plot representing the regulated genes in (*Fgf21*$^{hep+/+}$ Fasted vs *Fgf21*$^{hep+/+}$ Fed) vs (*Fgf21*$^{hep-/-}$ Fasted *vs Fgf21*$^{hep-/-}$ Fed) mice in liver samples. Green dots correspond to genes that are non-significant, and the red dot represents significant genes ($n = 6$ mice per group, biological replicates, $\log_2$FC >1: vertical line; $-\log_{10}(p_{adj})$ >0.05: horizontal line). (**B**, **C**) LogFC of top genes up- (**B**) and down-regulated (**C**) by fasting from *Fgf21* liver floxed (*Fgf21*$^{hep+/+}$) or *Fgf21* liver knockout (*Fgf21*$^{hep-/-}$) mice ($n = 6$ mice per group, biological replicates). (**D**) mRNA relative expression of liver *Fut1*, *Nat8*, *Car3*, *Slc13a4*, *Lgals1*, *Nkiras2*, *March2*, *Ube2l6*, *Tmem109*, *Tmem100*, *Myom1*, *Adamts14*, *Pigyl*, *Gm12*, *Zfyve21*, *Sfxn5*, *Nudt13*, and *Lect2*, derived from microarray results ($n = 6$ mice per group, biological replicates, Pearson correlation analysis to identify genes whose expression levels are correlated with that of *Fgf21*: correlation coefficient threshold >0.7 and for each represented gene: limma package, with linear models fitted (lmFit), Fed vs Fasted in *Fgf21*$^{hep+/+}$ or *Fgf21*$^{hep-/-}$, *Fut1*, $^{**}p_{adj} = 0.003$; *Nat8*, $^{***}p_{adj} = 0.0002$; *Car3*, $^{**}p_{adj} = 0.0019$; *Slc13a4*, $^{*}p_{adj} = 0.048$; *Lgals1*, $^{****}p_{adj} < 0.0001$; *Nkiras2*, $^{***}p_{adj} = 0.00018$; *March2*, $^{***}p_{adj} = 0.0006$; *Ube2l6*, $^{**}p_{adj} = 0.0016$; *Tmem109*, $^{*}p_{adj} = 0.0123$; *Tmem100*, $^{**}p_{adj} = 0.002$; *Myom1*, $^{****}p_{adj} < 0.0001$ (*Fgf21*$^{hep+/+}$), $^{*}p_{adj} = 0.033$ (*Fgf21*$^{hep-/-}$); *Adamts14*, $^{***}p_{adj} = 0.0005$; *Pigyl*, $^{***}p_{adj} = 0.0004$ (*Fgf21*$^{hep+/+}$), $^{*}p_{adj} = 0.031$ (*Fgf21*$^{hep-/--}$); *Gm12*, $^{**}p_{adj} = 0.0016$; *Zfyve21*, $^{***}p_{adj} = 0.00013$ (*Fgf21*$^{hep+/+}$), $^{*}p_{adj} = 0.0109$ (*Fgf21*$^{hep-/-}$); *Sfxn5*, $^{****}p_{adj} < 0.0001$ (*Fgf21*$^{hep+/+}$), $^{**}p_{adj} = 0.0031$ (*Fgf21*$^{hep-/-}$); *Nudt13*, $^{**}p_{adj} = 0.0012$; *Lect2*, $^{****}p_{adj} < 0.0001$); Fasted *Fgf21*$^{hep+/+}$ vs Fasted *Fgf21*$^{hep-/-}$, *Fut1*, $^{\#\#\#}p_{adj} = 0.0009$. (**E**) mRNA relative expression of *β-Klotho and Fgfr1c* in liver samples measured by qRT-PCR ($n = 8$–10 mice per group, biological replicates, two-way ANOVA followed by Šídák's multiple comparisons test $\alpha = 0.05$; Fed vs Fasted in *Fgf21*$^{hep+/+}$, *β-Klotho*, $^{**}p_{adj} = 0.008$; *Fgfr1c*, $^{*}p_{adj} = 0.02$). Data information: All data were presented as mean ± SEM; * shows a fasting effect; # shows a genotype effect.

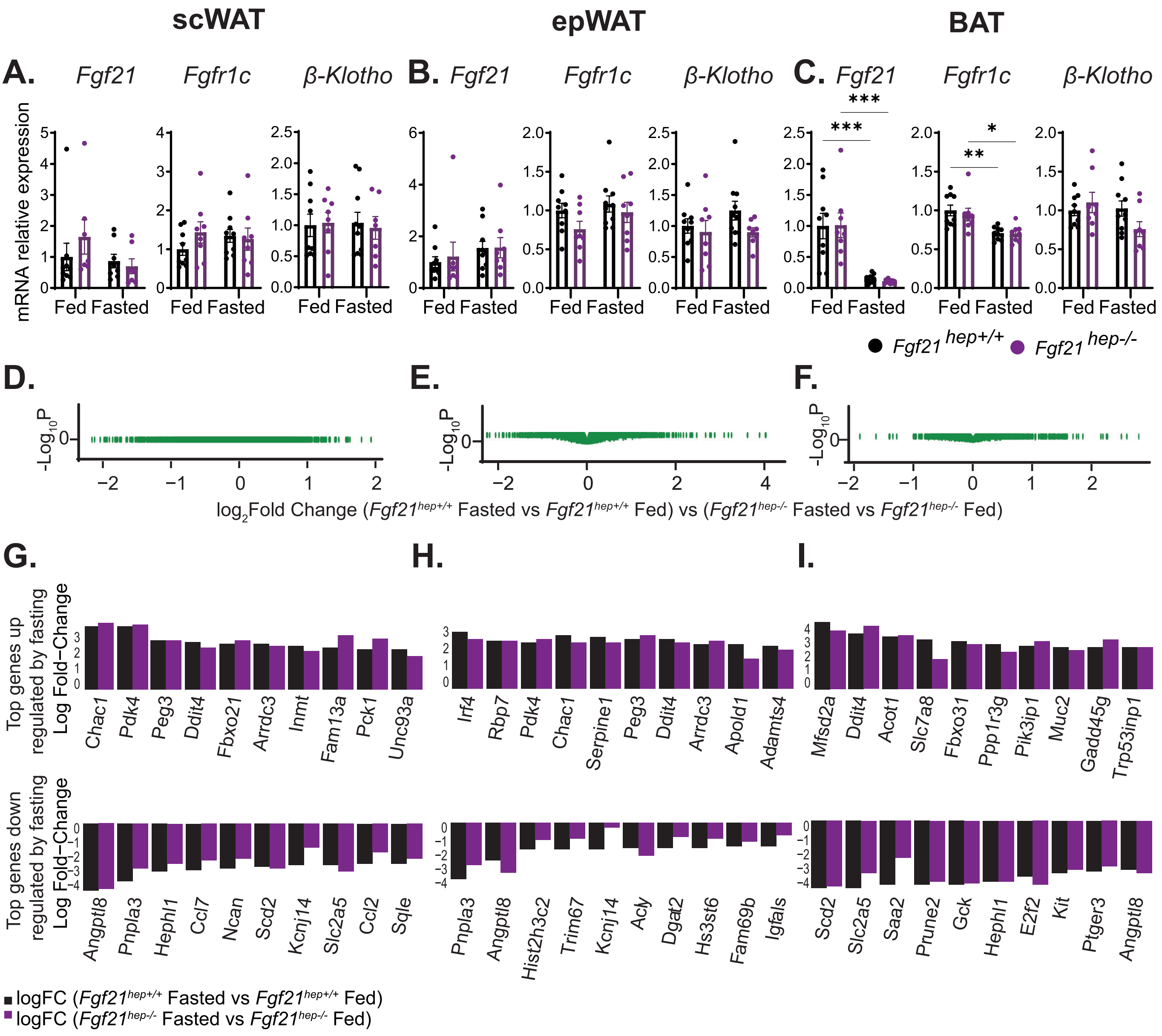

Figure EV3. Hepatocyte-specific deletion of *Fgf21* does not affect fasting-induced adipose tissue gene expression.

Related to Figs. 4 and 5. *Fgf21* liver floxed (*Fgf21*$^{hep+/+}$) or *Fgf21* liver knockout (*Fgf21*$^{hep-/-}$) mice were fed *ad libitum* or fasted for 20 h. (**A**) mRNA relative expression of *Fgf21*, *Fgfr1c*, and *β-Klotho* in subcutaneous white adipose tissue (scWAT) samples measured by qRT-PCR ($n = 7$–10 mice per group, biological replicates, two-way ANOVA followed by Šídák's multiple comparisons test $\alpha = 0.05$). (**B**) mRNA relative expression of *Fgf21*, *Fgfr1c*, and *β-Klotho* in epididymal white adipose tissue (epWAT) samples measured by qRT-PCR ($n = 8$–10 mice per group, biological replicates, two-way ANOVA followed by Šídák's multiple comparisons test $\alpha = 0.05$). (**C**) mRNA relative expression of *Fgf21*, *Fgfr1c*, and *β-Klotho* in brown adipose tissue (BAT) samples measured by qRT-PCR ($n = 7$–10 mice per group, biological replicates, two-way ANOVA followed by Šídák's multiple comparisons test $\alpha = 0.05$; Fed vs Fasted in *Fgf21*$^{hep+/+}$ or *Fgf21*$^{hep-/-}$, *Fgf21*, ***$p_{adj} = 0.0003$ (*Fgf21*$^{hep+/+}$), ***$p_{adj} = 0.0005$ (*Fgf21*$^{hep-/-}$); *Fgfr1c*, **$p_{adj} = 0.0019$, *$p_{adj} = 0.014$). (**D-F**) Volcano plot representing the regulated genes in (*Fgf21*$^{hep+/+}$ Fasted *vs Fgf21*$^{hep+/+}$ Fed) vs (*Fgf21*$^{hep-/-}$ Fasted *vs Fgf21*$^{hep-/-}$ Fed) mice in scWAT (**D**), epWAT (**E**), and BAT (**F**) samples. Green dots correspond to genes that are non-significant ($n = 6$ mice per group, biological replicates, $Log_2FC > 1$; under $-log_{10}(p_{adj}) > 0.05$). (**G-I**) LogFC of top genes up- and down-regulated by fasting from *Fgf21* liver floxed (*Fgf21*$^{hep+/+}$) or *Fgf21* liver knockout (*Fgf21*$^{hep-/-}$) mice ($n = 6$ mice per group, biological replicates) in scWAT (**G**), epWAT (**H**), and BAT (**I**) samples. Data information: All data were presented as mean ± SEM; * shows a fasting effect.

 © The Author(s)

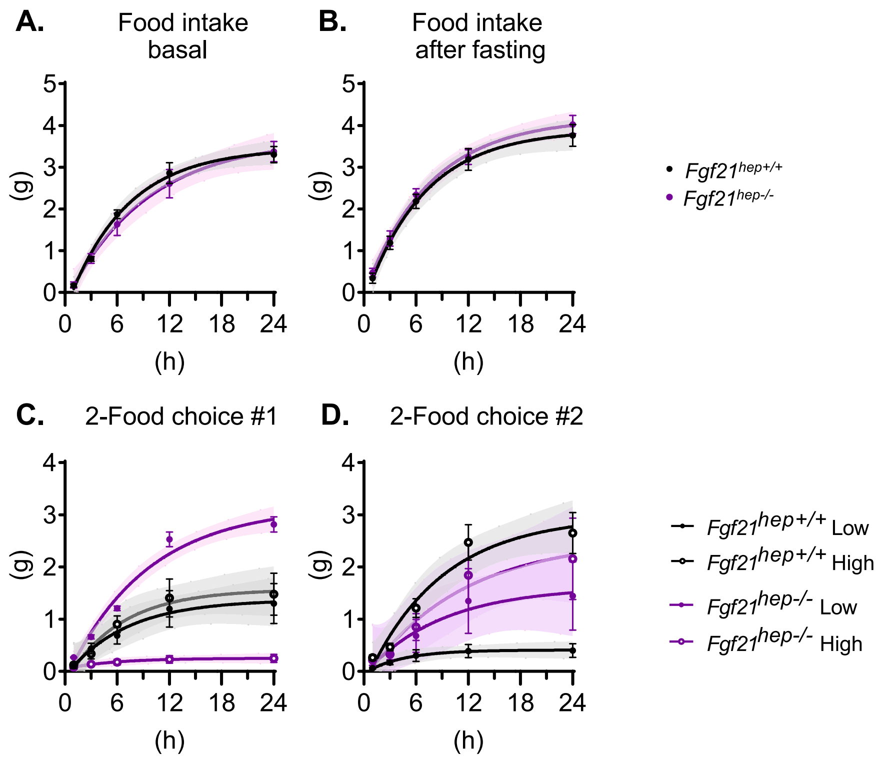

Figure EV4. Hepatic FGF21 is required for protein preference after fasting from the first hours of feeding.

Related to Fig. 6. (**A**, **B**) Food intake of singly housed mice was monitored over 24 h after 4, 6, 12, and 24 h in mice fed *ad libitum* with a standard diet (**A**) or refed *ad libitum* with a standard diet after 20 h of fasting (**B**) ($n = 5$–6 mice per group, nonlinear regression with an exponential plateau was fitted to the data, and 95% confidence bands were plotted). (**C**, **D**) Food intake of singly housed mice was monitored over 24 h after 4, 6, 12, and 24 h of refeeding of mice fed *ad libitum* with a low-protein diet (low-protein/high-carb diet: 6.5% protein and 80.4% carbohydrate) and a high-protein diet (high-protein/low-carb diet: 42.6% protein and 44.3% carbohydrate) (**C**) or *ad libitum* with a low-protein diet (low-protein/high-carb diet: 6.5% protein and 80.4% carbohydrate) and a high-protein diet (high-protein/high-carb diet: 17.8% protein, 70.4% carbohydrate) (**D**) ($n = 5$–6 mice per group, nonlinear regression with an exponential plateau was fitted to the data, and 95% confidence bands were plotted). Data information: Only the regression curves are shown on the graphs, with data points displayed as mean ± SEM.

© The Author(s)
