# Supplementary material for: Hepatic FGF21 is not required for fasting metabolism but guides protein appetite post energy depletion
Source: EMBO Rep. 2026 Apr 27;27(12):3189–213. doi: 10.1038/s44319-026-00790-9 (PMC13303862; doi:10.1038/s44319-026-00790-9)
Supplement: Supplementary file 2 — Peer Review File [file 44319_2026_790_MOESM2_ESM.pdf]

EMBO reports

Hepatic FGF21 is not required for fasting metabolism but guides protein appetite post energy depletion

Justine Bruse, Clothilde Marbach, Arnaud Polizzi, Tatiana Landre, Juliette Salvi, Valérie Alquier-Bacquier, Shiou-Ping Chen, Marine Huillet, Clemence Rives, Céline Martin, Prunelle Perrier, Fadila Benhamed, Marion Regnier, Stefan Weger, Claire Naylies, Yannick Lippi, Caroline Sommer, Mikael Albin, Frederic Lasserre, Thierry Levade, Michael Schupp, Laurence Gamet-Payrastre, Leon Kautz, Nicolas Loiseau, Walter WAHLI, Sandrine Ellero-Simatos, Céline Cruciani-Guglielmacci, Alexandra Montagner, Alexandre Benani, Catherine Postic, Herve Guillou, and Anne Fougerat

Corresponding author(s): Anne Fougerat (anne.fougerat@inrae.fr) , Herve Guillou (herve.guillou@inrae.fr)

Review Timeline:

Submission Date:	22nd Oct 25
Editorial Decision:	9th Jan 26
Revision Received:	13th Feb 26
Editorial Decision:	18th Mar 26
Revision Received:	23rd Mar 26
Accepted:	13th Apr 26

Editor: Kurt Weir

Transaction Report:

(Note: With the exception of the correction of typographical or spelling errors that could be a source of ambiguity, letters and reports are not edited. Depending on transfer agreements, referee reports obtained elsewhere may or may not be included in this compilation. Referee reports are anonymous unless the Referee chooses to sign their reports.)

Dear Dr. Fougerat

Thank you for the submission of your research manuscript to our journal. We have now received the full set of referee reports that is copied below.

As you have seen, the referees acknowledge that the findings are interesting and that the conclusions are overall supported by the data presented but they also raise a number of concerns and have suggestions how to further strengthen the data, particularly including greater discussion of the literature (particularly specific studies looking at full body FGF21 deletion) and an explanation relating to the use of Albumin-cre controls.

Given these constructive comments, we would like to invite you to revise your manuscript with the understanding that the referee concerns (as detailed above and in their reports) must be fully addressed and their suggestions taken on board. Please address all referee concerns in a complete point-by-point response. Acceptance of the manuscript will depend on a positive outcome of a second round of review. It is EMBO Reports policy to allow a single round of revision only and acceptance or rejection of the manuscript will therefore depend on the completeness of your responses included in the next, final version of the manuscript.

We realize that it is difficult to revise to a specific deadline. In the interest of protecting the conceptual advance provided by the work, we recommend a revision within 3 months (April 9th). Please discuss the revision progress ahead of this time with the editor if you require more time to complete the revisions.

I am also happy to discuss the revision further via e-mail or a video call, if you wish.

You can either publish the study as a short report or as a full article. For short reports, the revised manuscript should not exceed 27,000 characters (including spaces but excluding materials & methods and references) and 5 main plus 5 expanded view figures. The results and discussion sections must further be combined, which will help to shorten the manuscript text by eliminating some redundancy that is inevitable when discussing the same experiments twice. For a normal article there are no length limitations, but it should have more than 5 main figures and the results and discussion sections must be separate. In both cases, the entire materials and methods must be included in the main manuscript file.

================
IMPORTANT NOTE:

We perform an initial quality control of all revised manuscripts before re-review. Your manuscript will FAIL this control and the handling will be delayed IN CASE the following APPLIES:

1) A data availability section providing access to data deposited in public databases is missing. If you have not deposited any data, please add a sentence to the data availability section that explains that.

2) Your manuscript contains statistics and error bars based on n=2. Please use scatter blots in these cases. No statistics should be calculated if n=2.

When submitting your revised manuscript, please carefully review the instructions that follow below. Failure to include requested items will delay the evaluation of your revision.

================

When submitting your revised manuscript, we will require:

1) a .docx formatted version of the manuscript text (including legends for main figures, EV figures and tables). Please make sure that the changes are highlighted to be clearly visible.

2) individual production quality figure files as .eps, .tif, .jpg (one file per figure).
Please download our Figure Preparation Guidelines (figure preparation pdf) from our Author Guidelines pages https://www.embopress.org/page/journal/14693178/authorguide for more info on how to prepare your figures.

3) a .docx formatted letter INCLUDING the reviewers' reports and your detailed point-by-point responses to their comments. As part of the EMBO Press transparent editorial process, the point-by-point response is part of the Review Process File (RPF), which will be published alongside your paper.

4) a complete author checklist, which you can download from our author guidelines (). Please insert information in the checklist

that is also reflected in the manuscript. The completed author checklist will also be part of the RPF.

5) Please note that all corresponding authors are required to supply an ORCID ID for their name upon submission of a revised manuscript (). Please find instructions on how to link your ORCID ID to your account in our manuscript tracking system in our Author guidelines
()

6) We replaced Supplementary Information with Expanded View (EV) Figures and Tables that are collapsible/expandable online. A maximum of 5 EV Figures can be typeset. EV Figures should be cited as 'Figure EV1, Figure EV2" etc... in the text and their respective legends should be included in the main text after the legends of regular figures.

- For the figures that you do NOT wish to display as Expanded View figures, they should be bundled together with their legends in a single PDF file called *Appendix*, which should start with a short Table of Content. Appendix figures should be referred to in the main text as: "Appendix Figure S1, Appendix Figure S2" etc. See detailed instructions regarding expanded view here:

- Additional Tables/Datasets should be labeled and referred to as Table EV1, Dataset EV1, etc. Legends have to be provided in a separate tab in case of .xls files. Alternatively, the legend can be supplied as a separate text file (README) and zipped together with the Table/Dataset file.

7) Before submitting your revision, primary datasets (and computer code, where appropriate) produced in this study need to be deposited in an appropriate public database (see <
https://www.embopress.org/page/journal/14693178/authorguide#dataavailability>).

Please remember to provide a reviewer password if the datasets are not yet public.

The accession numbers and database should be listed in a formal "Data Availability " section (placed after Materials & Method) that follows the model below (see also < https://www.embopress.org/page/journal/14693178/authorguide#dataavailability>). Please note that the Data Availability Section is restricted to new primary data that are part of this study.

Data availability

The datasets (and computer code) produced in this study are available in the following databases:

- RNA-Seq data: Gene Expression Omnibus GSE46843 (https://www.ncbi.nlm.nih.gov/geo/query/acc.cgi?acc=GSE46843)
- [data type]: [name of the resource] [accession number/identifier/doi] ([URL or identifiers.org/DATABASE:ACCESSION])

*** Note - All links should resolve to a page where the data can be accessed. ***

8) At EMBO Press we ask authors to provide source data for the main manuscript figures. You will receive a separate email with instructions for providing source data with your revised manuscript, including how to upload and organize the files.

Additional information on source data and instruction on how to label the files are available

9) The journal requires a statement specifying whether or not authors have competing interests (defined as all potential or actual interests that could be perceived to influence the presentation or interpretation of an article). In case of competing interests, this must be specified in your disclosure statement. Further information: https://www.embopress.org/competing-interests

10) Figure legends and data quantification:
The following points must be specified in each figure legend:

- the name of the statistical test used to generate error bars and P values,
- the EXACT p-values,
- the number (n) of independent experiments (please specify technical or biological replicates) underlying each data point,
- the nature of the bars and error bars (s.d., s.e.m.)

- If the data are obtained from n {less than or equal to} 5, show the individual data points in addition to the SD or SEM.
- If the data are obtained from n {less than or equal to} 2, use scatter blots showing the individual data points.

Discussion of statistical methodology can be reported in the materials and methods section, but figure legends should contain a basic description of n, P and the test applied.

See also the guidelines for figure legend preparation:
https://www.embopress.org/page/journal/14693178/authorguide#figureformat

- Please also include scale bars in all microscopy images and define their size in the legend, not the image.

11) Our journal encourages inclusion of *data citations in the reference list* to directly cite datasets that were re-used and obtained from public databases. Data citations in the article text are distinct from normal bibliographical citations and should directly link to the database records from which the data can be accessed. In the main text, data citations are formatted as follows: "Data ref: Smith et al, 2001" or "Data ref: NCBI Sequence Read Archive PRJNA342805, 2017". In the Reference list, data citations must be labeled with "[DATASET]". A data reference must provide the database name, accession number/identifiers and a resolvable link to the landing page from which the data can be accessed at the end of the reference. Further instructions are available at .

12) All Materials and Methods need to be described in the main text using our 'Structured Methods' format. According to this format, the Methods section includes a Reagents and Tools Table (listing key reagents, experimental models, software and relevant equipment and including their sources and relevant identifiers) followed by a Methods and Protocols section describing the methods, ideally using a step-by-step protocol format. The aim is to facilitate adoption of the methodologies across labs. Please download and fill our Reagents and Tools Table template (.docx), which you can find in our author guidelines: https://www.embopress.org/page/journal/14693178/authorguide#structuredmethods.
When submitting your revised manuscript, please do not include the Reagents and Tools Table in the Methods section of the manuscript but upload it as a separate file choosing the file type "Reagent Table".
An example of a Method paper with Structured Methods can be found here: https://www.embopress.org/doi/10.15252/msb.20178071.

13) As part of the EMBO publication's Transparent Editorial Process, EMBO Reports publishes online a Review Process File to accompany accepted manuscripts. This File will be published in conjunction with your paper and will include the referee reports, your point-by-point response and all pertinent correspondence relating to the manuscript.

You are able to opt out of this by letting the editorial office know (emboreports@embo.org). If you do opt out, the Review Process File link will point to the following statement: "No Review Process File is available with this article, as the authors have chosen not to make the review process public in this case."

We would also welcome the submission of cover suggestions, or motifs to be used by our Graphics Illustrator in designing a cover.

I look forward to seeing a revised form of your manuscript when it is ready and please let me know if you have questions or comments regarding the revision.

Yours sincerely,

Kurt Weir
Editor
EMBO Reports

Referee #1:

This paper is extremely important because it corrects the very common notion that the effect of PPARa on ketogenesis and fatty acid oxidation is mediated by the induction of FGF21. While FGF21 is highly inducible by PPARa, which is essential for the regulation of hepatic ketogenesis, the data in the present paper clearly show that the loss of hepatic production of FGF21 does not influence hepatic gluconeogenesis, adipose tissue lipolysis, or ketone production. Instead, the data support the notion that liver-derived FGF21 acts in the brain and regulates nutrient preference.

The studies are very carefully done using the appropriate controls. The data are timely, given the importance of FGF21 as a pharmacological target and ongoing clinical trials with FGF21 analogs. The paper helps correct a widely propagated yet incorrect narrative using a very solid set of studies and the use of appropriate animal models.

In the concluding statement, it is written that "Unlike other studies in Fgf21-/- mice (Badman et al., 2009; Hotta et al., 2009; Potthoff et al., 2009; Liang et al., 2014), our work reveals that hepatocyte-restricted deletion of Fgf21 does not influence fasting response." This sentence is incorrect, since none of the other papers studied the effect of hepatocyte-restricted deletion of Fgf21 and some did not even study fasting. Hotta found that ketogenesis is not impaired in whole-body FGF21 KO mice. Badman studied fasting but did only a very shallow analysis. Please adjust the wording.

The discussion would benefit from an elaboration on FGf21 as a stress-induced hepatokine that alters nutrient preference. This notion should replace the widespread misconception that Fgf21 mediates the induction of hepatic ketogenesis and fatty acid oxidation by PPARa, which is clearly incorrect.

Referee #2:

In the manuscript "Hepatocyte FGF21 is not required for fasting-induced metabolic responses but guides protein appetite after energy depletion", Bruse Justine and colleague investigate the physiological role of hepatocyte-derived FGF21 in the metabolic and behavioural adaptations to fasting. Using a hepatocyte-specific knockout model, the authors convincingly demonstrate that, despite being the primary source of circulating FGF21 during fasting, hepatocyte FGF21 is not required for the classical metabolic responses to fasting: Fgf21-deficient mice maintain normal fasting-induced hypoglycemia, adipose-tissue lipolysis, gluconeogenesis, fatty-acid oxidation, and ketogenesis, and exhibit hepatic transcriptomic and metabolomic changes largely indistinguishable from those of wild-type animals. Likewise, fasting-induced remodelling of white and brown adipose depots proceeds unaffected by the loss of liver Fgf21. These findings challenge long-standing models that portray FGF21 as a central fasting hormone orchestrating systemic metabolic adaptation: despite evidences generated in obesity context, whole body KO models or by super-physiological overexpression (by KI models) are not contradicted, the authors make a clear point (that I share in light of the data presented) that at physiological concentrations, the situation might be more nuanced. Similar point was made for other metabolic hormones like GLP1 in the past where "pharmacological doses" exert effects that are not seen in physiological contexts.

Notably, the study also identifies a distinct and physiologically relevant role for hepatocyte-derived FGF21 in guiding post-fasting macronutrient selection. During refeeding, Fgf21hep-/- mice fail to develop the strong protein preference observed in controls, despite comparable overall food intake. This work therefore redefines FGF21 as a liver-brain endocrine signal that supports the selection of protein-rich foods after energy depletion, rather than as a driver of fasting metabolism itself.

The manuscript is clear, written in good English and discussion is well tailored to the results. The reconceptualization of FGF21 has implications for therapeutic strategies targeting metabolic diseases and appetite regulation which is a hot topic these days in research focussing on obesity, T2D and their complications (e.g. MASLD).

The Key Strengths of the Study in my opinion are: 1) Conceptual innovation: the study convincingly overturns the view of FGF21 as a canonical fasting hormone, offering a revised and more nuanced theory of its action; 2) a clear mechanistic dissection: the hepatocyte-specific KO model isolates the liver's contribution to FGF21 physiology with high precision; 3) the comprehensive multi-tissue analysis: integrated transcriptomic, metabolomic, physiological, and behavioural datasets provide a robust systems-biology characterization; 3) the methodological rigor and the fact that key experiments have been performed in both sexes.

I have just minor suggestions to improve the quality of the manuscript:

1) Please append in supplementary the full list of DEGs (unfiltered for P) of the hepatic and AT transcriptomes in the different comparisons. Same for pathways and transcription factor analyses (with significances and list of the genes enriching the pathway). This is very useful information for the field and will attract collaborations and citations (despite I do not dispute raw data have been deposited ... not all the groups have bioinformatic capacities to replicate the analyses).

2) Please clarify in the figures and legends if significance refers to P or rather to FDR for omics data; from the methods section, looks like the data have been FDR corrected but in the figures authors refer to P value. Also, are the comparisons powered enough to conclude that no differences are observed among genotypes? Authors have decided to use a really robust but also very restrictive statistical approach so the risk of Type 2 error (false negatives) is at least possible (I would therefore show in the aforementioned supp table both the P and the FDR). Please consider that despite FDR correction is a rigorous approach for repeated measures, it assumes that variables are INDEPENDENT (which is not the case for gene expression ... that's why we all run pathway analyses ...).

3) Array heatmaps. Despite I do not dispute the value of the unbiased clustering on top (showing that there is no clear separation among genotypes), I wonder if having a biased ordering with WT/KO subgroups clearly separated would be graphically more informative to show that no major differences can be observed among genotypes.

4) The graphical representation of the 2 way ANOVA is a bit confusing in all the figures. Would not be clearer just to write on each panel the 3 relevant Ps (treatment/genotype/interaction), and asterisks/letters for those comparisons significant at the post-hoc analysis?

5) While I see the choice of splitting the results of the same experiment in different figures, would be useful to the reader to have a more comprehensive understanding of the phenotype of the different genotypes for each experiment with respect to BW, Insulin, Glucose, NEFA, TG, CHOL, glycerol, etc (if and when available); supplementary table? Currently the reader needs to hunt these data here and there in the manuscript (and they are not all systematically available). Which is a pity after all this work.

6) Fig 1G and Pyruvate tolerance test in supplementary figure: can you calculate AUC? Looks like there might be a trending difference in β-hydroxybutyrate (in fasting; ZT 16-20) and pyruvate response among genotypes.

7) Provocative question (to improve discussion): if no genotype-specific DEGs are identified in fasting, how the genotype effect on EE and substrate utilisation (calorimetry) can be justified? Do the authors have any mechanistic explanation?

8) Please when you resubmit the manuscript:
a. add figure number on each of them with a single combined PDF has been challenging to understand which figure was which while scrolling up and down between legend and figure ...
b. verify that each figure panel has its own letter (e.g. The autophagy gene expression panel labelling, and letter for Angptl4 and Angptl6 sections are missing)

9) It would be nice to have high-resolution pictures of the stainings;

10)The axis of some graphs not always starts form zero; moreover, kindly use the same scale ranges while comparing male-female differences (For example, male FGF21 uses a different scale range from females) to make less challenging to compare the data.

Really well done!

Selected points from cross-comment:

The main experimental concern to be addressed is from Rev 3 (Why were albumin-cre mouse not used as controls) that i largely share (well spotted!).

The rest of the comments relates to improvements in discussion/presentation of the data: I agree with other reviewers that a general issue with readability and figure presentation of the results makes the manuscript difficult to follow, and that discussion of the data and literature can be improved.

Referee #3:

This paper was difficult to review because of combination of formatting and missing data. In addition the strategy for the specific studies included and an adequate interpretation are poorly described. While the authors argue that most studies evaluating the physiology of FGF21 in the fed and fasted state were performed in mice with total body deletion of FGF21 they are either unaware or not interested in discussing studies with liver specific FGF21 deletion. This includes a 2019 paper by Ameka et al as well as a 2020 manuscript on the role of hepatic FGF21 in mediating glycemic versus ketogenic responses by Watanabe et al. These previous publications would need to be directly addressed in the discussion.

An additional question is why were albumin-cre mouse not used as controls as cre mice can have unexpected metabolic phenotypes that may obscure the flox-cre mice with specific gene deletion?

With regard to poor formatting and missing data. The manuscript is not paginated, and the figures are not labelled. Where are figures 1-2 and three? They are referred to in the text but I do not see them anywhere within the printed manuscript which I have. For figures 4 and 5 for which there are figure legends, the legends do not match the text. For example the food choice figure legend for Figure 5 refers to panels A through F but only four panels are displayed in the figure which appears to be on the last page of the manuscript. A similar problem exists with Figure 4 where the number of panels are described as A to N but only panels a through H are shown. As this figure looks at changes in adipose tissue it is surprising that BAT is not examined as FGF21 has substantial effects on BAT both in the fasted state and when animals are eating a ketogenic diet. It seems that none of the authors paid adequate attention to the final assembly of the manuscript.

The figures labeled as expanded view figures are poorly described in the figure legends. For example for EV1 which is supposed to distinguish between liver floxed mice and liver mice with FGF 21 deletion of the assumptions regarding what is being shown in the figure legends on page 17 is unclear presumably fed and fasted are uh following the 20 hour fast described in the EV1 figure legend on page 15. It is difficult to determine what the expanded view figure should be matched with for example is the figure on page 19 which shows fed and fasted levels with both control and a ketogenic diet supposed to be Figure 1? As a specific question why is relative liver weight reported as relative liver weight as a percent of body weight and what I assume is extended figure one on page 17 rather absolute liver weight I do not find an indication that the weights of the mice were recorded and in fact this needs to be shown as data and liver weight should actually be shown as absolute rather than relative to total body weight another point is that while reportedly there are no differences between fed and fasted animals looking at figure I believe extended figure 1B hepatic triglycerides hepatic triglycerides appear to be higher in fasted FGF 21 knockout mice as uh this numbers of animals are small it's really difficult to tell if this is statistically significant

On page what I believe is 21 differentially expressed genes by fasting and FGF 21 intact and FGF 21 liver specific knockout figure 1A it's really difficult to understand what is being shown on the basis of the way that the figure was formatted and showing the log full change also obscures potential differences between the groups.

Multiple panels in EV-5 show differences in gene expression (eg: Fut1) but the potential significance of these differences in the fasted state are not discussed.

Selected points from cross-comment:

Key missing experiments are as basic as food preference. The data presented is a shift in protein preference after fasting. While the authors refer to the studies of Laeger et al, they do not note the differences and the requirements in changes of baseline diets to elicit protein preference. Furthermore while the conclusion is that since no changes in fasting and responses to ketogenic diet are detected in liver specific FGF21 deleted mice and that therefore FGF21 must be acting through the brain no data specific to this conclusion are shown so this is in effect a confirmation of the previous studies by Laeger. Antonellis et al, Front Endocrinol 2016 confirmed that FGF21 null mice do not exhibit did not have an impaired response to fasting, another point in which the specific hepatic deletion of FGF21 becomes confirmatory, another report that is not referenced. Similarly Von Holstein-Rathlou showed that total body FGF21 deletion increased sweet preference. Since circulating FGF21 is almost entirely derived from hepatocytes, deletion of the gene in hepatocytes would be expected to reproduce much of the total body deletion so much of the data "collapses" to a confirmatory study. The weight stability in the context of consumption of a ketogenic diet remains perplexing.

Finally with regard to impact -- much of the data presented is confirmatory of total body deletion because the "endocrine" functions of FGF21 are already well mapped to the hepatocyte release.

There is inconsistency with other reports with regard to the liver and ketogenic diets. For example weights of female mice consuming a ketogenic diet are shown to be the same (panel H -- unclear which figure) despite the fact that diets that reliable reduce ketosis ither prevent weight gain or are associated with weight loss in obese animals. Attenuated weight gain has been shown by multiple groups including Poplawski et al, PLos One, 2011, Jornayvaz et al AJP 2010.

Point-by-point response to referees' comments

Referee #1:

This paper is extremely important because it corrects the very common notion that the effect of PPARa on ketogenesis and fatty acid oxidation is mediated by the induction of FGF21. While FGF21 is highly inducible by PPARa, which is essential for the regulation of hepatic ketogenesis, the data in the present paper clearly show that the loss of hepatic production of FGF21 does not influence hepatic gluconeogenesis, adipose tissue lipolysis, or ketone production. Instead, the data support the notion that liver-derived FGF21 acts in the brain and regulates nutrient preference.

The studies are very carefully done using the appropriate controls. The data are timely, given the importance of FGF21 as a pharmacological target and ongoing clinical trials with FGF21 analogs. The paper helps correct a widely propagated yet incorrect narrative using a very solid set of studies and the use of appropriate animal models.
We thank Referee #1 for his/her supportive comments, his/her suggestions to improve our work, and for recognizing the importance of our findings.

In the concluding statement, it is written that "Unlike other studies in Fgf21-/- mice (Badman et al., 2009; Hotta et al., 2009; Potthoff et al., 2009; Liang et al., 2014), our work reveals that hepatocyte-restricted deletion of Fgf21 does not influence fasting response." This sentence is incorrect, since none of the other papers studied the effect of hepatocyte-restricted deletion of Fgf21 and some did not even study fasting. Hotta found that ketogenesis is not impaired in whole-body FGF21 KO mice. Badman studied fasting but did only a very shallow analysis. Please adjust the wording.
We fully agree with Referee #1 that the wording of this sentence was not correct. In the revised version of the manuscript, we have removed this sentence to avoid inappropriate comparisons with previous studies.
In addition, in agreement with the editor, we have changed the format of the manuscript from a Report to a full Research Article. This has allowed us to expand the discussion and provide a more comprehensive view of prior studies.

The discussion would benefit from an elaboration on FGf21 as a stress-induced hepatokine that alters nutrient preference. This notion should replace the widespread misconception that Fgf21 mediates the induction of hepatic ketogenesis and fatty acid oxidation by PPARa, which is clearly incorrect.
We thank Referee #1 for his/her suggestion. We have expanded the discussion accordingly.

Referee #2:

In the manuscript "Hepatocyte FGF21 is not required for fasting-induced metabolic responses but guides protein appetite after energy depletion", Bruse Justine and colleague investigate the physiological role of hepatocyte-derived FGF21 in the metabolic and behavioural adaptations to fasting. Using a hepatocyte-specific knockout model, the authors convincingly demonstrate that, despite being the primary source of circulating FGF21 during fasting, hepatocyte FGF21 is not required for the classical metabolic responses to fasting: Fgf21-deficient mice maintain normal fasting-induced hypoglycemia, adipose-tissue lipolysis, gluconeogenesis, fatty-acid oxidation, and ketogenesis, and exhibit hepatic transcriptomic and metabolomic changes largely indistinguishable from those of wild-type animals. Likewise, fasting-induced remodelling of white and brown adipose depots proceeds unaffected by the loss of liver Fgf21. These findings challenge long-standing models that portray FGF21 as a central fasting hormone orchestrating systemic metabolic adaptation: despite evidences generated in obesity context, whole body KO models or by super-physiological overexpression (by KI models) are not contradicted, the authors make a clear point (that I share in

light of the data presented) that at physiological concentrations, the situation might be more nuanced. Similar point was made for other metabolic hormones like GLP1 in the past where "pharmacological doses" exert effects that are not seen in physiological contexts.

Notably, the study also identifies a distinct and physiologically relevant role for hepatocyte-derived FGF21 in guiding post-fasting macronutrient selection. During refeeding, Fgf21hep-/- mice fail to develop the strong protein preference observed in controls, despite comparable overall food intake. This work therefore redefines FGF21 as a liver-brain endocrine signal that supports the selection of protein-rich foods after energy depletion, rather than as a driver of fasting metabolism itself.

The manuscript is clear, written in good English and discussion is well tailored to the results. The reconceptualization of FGF21 has implications for therapeutic strategies targeting metabolic diseases and appetite regulation which is a hot topic these days in research focussing on obesity, T2D and their complications (e.g. MASLD).

The Key Strengths of the Study in my opinion are: 1) Conceptual innovation: the study convincingly overturns the view of FGF21 as a canonical fasting hormone, offering a revised and more nuanced theory of its action; 2) a clear mechanistic dissection: the hepatocyte-specific KO model isolates the liver's contribution to FGF21 physiology with high precision; 3) the comprehensive multi-tissue analysis: integrated transcriptomic, metabolomic, physiological, and behavioural datasets provide a robust systems-biology characterization; 3) the methodological rigor and the fact that key experiments have been performed in both sexes.
We thank Referee #2 for his/her thorough and positive evaluation of our manuscript. We are pleased that the reviewer finds the data interesting and convincing.

I have just minor suggestions to improve the quality of the manuscript:
We thank Referee #2 for the careful reading and the constructive suggestions, which we believe significantly improve the quality, clarity, and accessibility of the manuscript. We have addressed each point as follows:

1) Please append in supplementary the full list of DEGs (unfiltered for P) of the hepatic and AT transcriptomes in the different comparisons. Same for pathways and transcription factor analyses (with significances and list of the genes enriching the pathway). This is very useful information for the field and will attract collaborations and citations (despite I do not dispute raw data have been deposited ... not all the groups have bioinformatic capacities to replicate the analyses).
We fully agree that providing complete datasets will be highly valuable to the field. We have now provided data source files associated with all main figures. In particular, the data source files for Figures 3, 4, and 5 now contain:

- the full lists of differentially expressed genes (including non-significant genes, with raw P values and FDR-adjusted values (p_{adj}) for hepatic and adipose tissue transcriptomes across all relevant comparisons;
- full pathway and transcription factor enrichment results, including enrichment statistics and the list of genes contributing to each pathway.

2) Please clarify in the figures and legends if significance refers to P or rather to FDR for omics data; from the methods section, looks like the data have been FDR corrected but in the figures authors refer to P value. Also, are the comparisons powered enough to conclude that no differences are observed among genotypes? Authors have decided to use a really robust but also very restrictive statistical approach so the risk of Type 2 error (false negatives) is at least possible (I would therefore show in the aforementioned supp table both the P and the FDR). Please consider that despite FDR correction is a rigorous approach for repeated measures, it assumes that variables are INDEPENDENT (which is not the case for gene expression ... that's why we all run pathway analyses ...).

We thank the reviewer for this important point. We have clarified in the figure legends that statistical significance for omics analyses refers to FDR-adjusted values (p_{adj}). We also now explicitly report both raw ANOVA P values (interaction/nutritional condition/genotype) and FDR-adjusted values (p_{adj}) in the data source files.

3) Array heatmaps. Despite I do not dispute the value of the unbiased clustering on top (showing that there is no clear separation among genotypes), I wonder if having a biased ordering with WT/KO subgroups clearly separated would be graphically more informative to show that no major differences can be observed among genotypes.

We thank the reviewer for this graphical suggestion. We deliberately chose an unbiased clustering approach in the main figures, as it directly illustrates that samples from different genotypes are mixed and that genotype does not drive major transcriptional differences under both fed and fasted conditions.

However, we have generated below the heatmaps for liver and adipose tissues in which samples are ordered by genotype, showing also that the absence of hepatocyte *Fgf21* does not induce major transcriptional changes in liver, and white and brown adipose tissues.

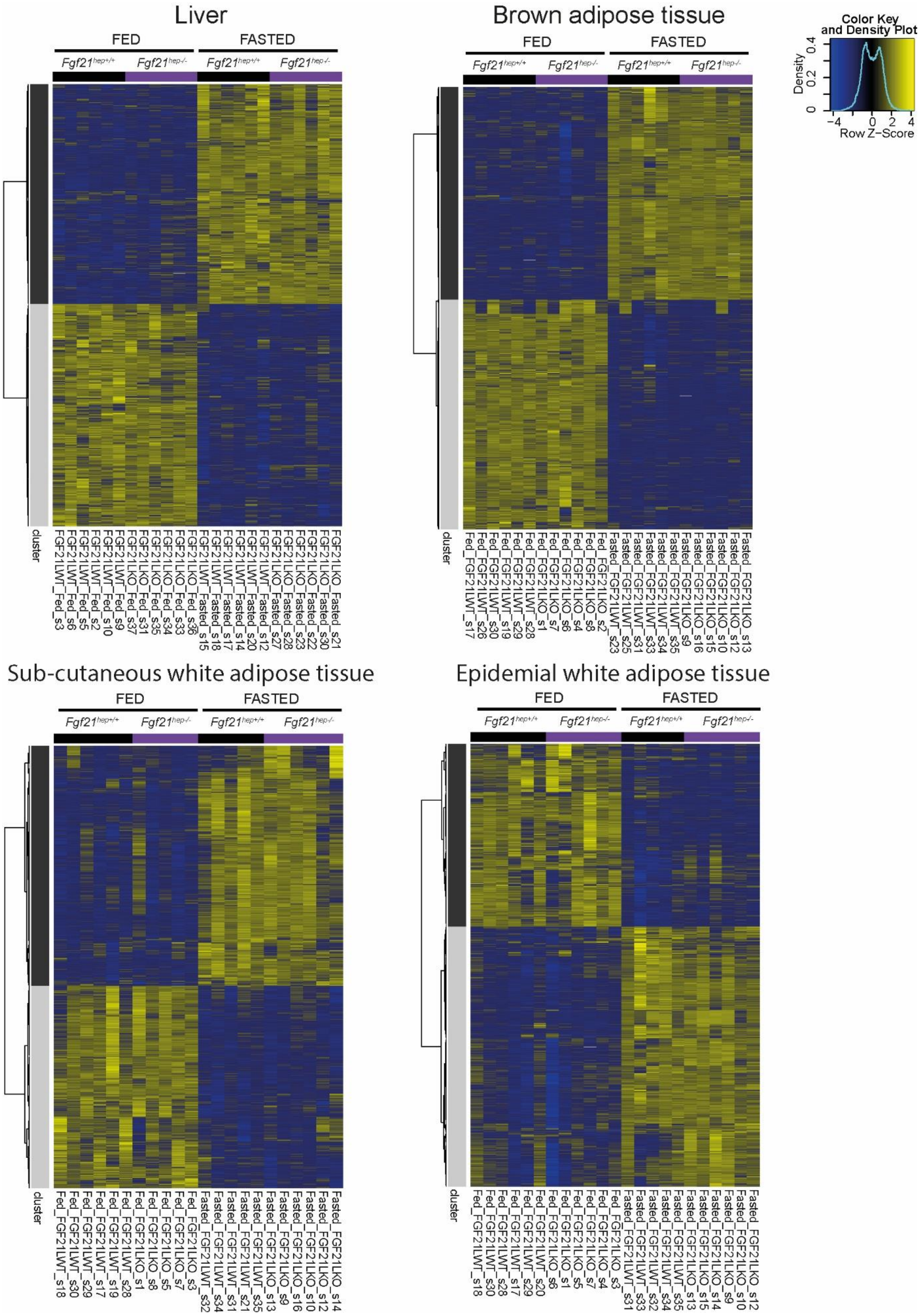
Liver
Brown adipose tissue
Sub-cutaneous white adipose tissue
Epidemial white adipose tissue
FED
FASTED
Fgf21hep+/+
Fgf21hep-/-
cluster
Color Key and Density Plot
Density
Row Z-Score

4) The graphical representation of the 2 way ANOVA is a bit confusing in all the figures. Would not be clearer just to write on each panel the 3 relevant Ps (treatment/genotype/interaction), and asterisks/letters for those comparisons significant at the post-hoc analysis?

We agree that the presentation of the two-way ANOVA results can be improved, especially in the kinetics in Figure 1. We have revised the figures to improve readability and consistency across figures.

While we appreciate the suggestion to display all P values (genotype, nutritional condition, and interaction) directly on each panel, we believe that it will reduce graphical clarity given the number of conditions and comparisons. In line with the *EMBO Reports* guidelines for revision, we have reported the exact adjusted P values for each relevant comparison in the figure legends. Data source files also contain all p values (genotype, nutritional condition, and interaction) for all figures. This provides full statistical transparency while maintaining figure readability.

5) While I see the choice of splitting the results of the same experiment in different figures, would be useful to the reader to have a more comprehensive understanding of the phenotype of the different genotypes for each experiment with respect to BW, Insulin, Glucose, NEFA, TG, CHOL, glycerol, etc (if and when available); supplementary table? Currently the reader needs to hunt these data here and there in the manuscript (and they are not all systematically available). Which is a pity after all this work.

We thank the reviewer for this suggestion. In the main figures, we initially chose to organize the data by tissue-specific responses (for example, hepatic triglycerides and β-hydroxybutyrate in Figure 2 related to liver metabolism, and glycerol and free fatty acids in Figure 4 related to white adipose tissue).

In accordance with *EMBO Reports* revision guidelines, we have provided the data source files associated with all main figures corresponding to the fasting experiment in males. To further address the reviewer's concern, we have now pooled all data obtained from the fasting experiment in females into a single figure (now Appendix Figure S1). Similarly, all data obtained from the ketogenic diet experiment have been pooled into a single figure (now Appendix Figure S2). We agree that this revised presentation provides a better overview of the phenotype of the different genotype in each experiment.

6) Fig 1G and Pyruvate tolerance test in supplementary figure: can you calculate AUC? Looks like there might be a trending difference in β-hydroxybutyrate (in fasting; ZT 16-20) and pyruvate response among genotypes.

The AUC analysis corresponding to the pyruvate tolerance test was already included in Figure EV3 (now Figure EV1 in the revised manuscript). The calculated AUC values were 19905 ± 402.245261 for *Fgf21*$^{hep+/+}$ mice and 20870 ± 894.3774022 for *Fgf21*$^{hep-/-}$ mice. Statistical analysis using unpaired Student's t-test revealed no statistical difference between genotypes (p=0.322284551).

In addition, we have now calculated the AUC for the fasting β-hydroxybutyrate levels in Figure 1G (graph below). While a trend is observed, it do not reach statistical significance (AUC = 11.24444 ± 0.885549 for fed *Fgf21*$^{hep+/+}$ mice, AUC = 10.075 ± 0.875816 for fed *Fgf21*$^{hep-/-}$ mice, AUC = 27.68 ± 1.212967 for fasted *Fgf21*$^{hep+/+}$ mice, AUC = 31.075 ± 2.446554 for fasted *Fgf21*$^{hep-/-}$ mice, Two-way ANOVA followed by Sidak's post hoc test Fasted *Fgf21*$^{hep+/+}$ vs Fasted *Fgf21*$^{hep-/-}$: p_{adj}=0.1977.

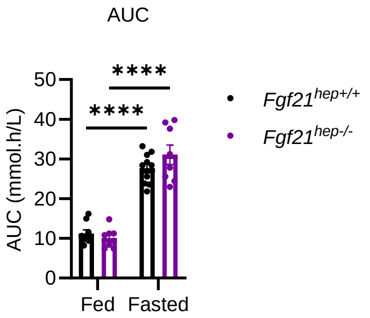

- Two-way ANOVA α=0.05:
  - Interaction P value = 0.1262
  - Nutritional condition P value < 0.0001
  - Genotype P value = 0.4493

- Post hoc: Šídák's multiple comparisons test α=0.05:
  - Fed *vs* Fasted in *Fgf21*$^{hep+/+}$ or *Fgf21*$^{hep-/-}$:

****p_{adj}<0.0001

- Fed *Fgf21*$^{hep+/+}$ vs Fed *Fgf21*$^{hep-/-}$: p_{adj}=0.8216
- Fasted *Fgf21*$^{hep+/+}$ vs Fasted *Fgf21*$^{hep-/-}$: p_{adj}=0.1977

In addition, the FDR-adjusted (p_{adj}) values between genotypes for all different ZT are now reported in the data source file and in the figure legend associated with Figure 1G : two-way ANOVA followed by Šídák's multiple comparisons test α=0.05, ZT4, **p_{adj}= 0.029, ****p_{adj}<0.0001, #p_{adj}=0.0467 (Fasted *Fgf21*$^{hep+/+}$ vs Fasted *Fgf21*$^{hep-/-}$); ZT8, *p_{adj}=0.0445, **p_{adj}=0.0097; ZT12, ***p_{adj}=0.0002, **p_{adj}=0.0089; ZT16-20-24, ****p_{adj}<0.001).

7) Provocative question (to improve discussion): if no genotype-specific DEGs are identified in fasting, how the genotype effect on EE and substrate utilisation (calorimetry) can be justified? Do the authors have any mechanistic explanation?
We thank Referee #2 for this relevant comment.
In accordance with the absence of major metabolic alterations during fasting, the indirect calorimetry data indicate that energy expenditure and substrate utilization during fasting were largely comparable between genotypes. We observed a modest increase in energy expenditure in the absence of hepatocyte *Fgf21* mostly during the refed phase. These data support our findings that hepatocyte *Fgf21* is dispensable for fasting metabolism but contributes to the physiological transition following nutrient availability.
Total energy expenditure integrates basal metabolism, physical activity and thermogenesis. Given that locomotor activity and substrate utilization are largely similar between genotypes, the increased energy expenditure observed in *Fgf21*$^{hep-/-}$ mice during refeeding is more consistent with altered thermogenesis associated with nutrient handling during the refed state. The underlying mechanisms may involve central effects of FGF21 or altered brown/beige adipose tissue activation. However our study focuses on the effect of hepatocyte *Fgf21* deletion during fasting and do not allow us to mechanistically address the differences observed upon refeeding. Addressing this point would require dedicated analyses in the refed state and is beyond the scope of the current study.

8) Please when you resubmit the manuscript:
a. add figure number on each of them with a single combined PDF has been challenging to understand which figure was which while scrolling up and down between legend and figure ...
b. verify that each figure panel has its own letter (e.g. The autophagy gene expression panel labelling, and letter for Angptl4 and Angptl6 sections are missing)
We apologize for the inconvenience. All figures have now been clearly numbered, and each panel has been assigned a unique letter where appropriate. For the figures without panel lettering (the autophagy gene panel and the hepatokine gene panel), we chose to include them in the Appendix Figures (Appendix Figure S3 and Appendix Figure S4, respectively) in the revised version of the manuscript. Accordingly, following the *EMBO Reports* format, the Appendix Figures file contains each figure immediately followed by its legend to facilitate comprehension. No individual lettering is assigned to each gene, as these genes are interpreted and discussed collectively based on their shared functional category (autophagy or hepatokines), rather than individually.

9) It would be nice to have high-resolution pictures of the stainings;
High-resolution versions of all histological images are now provided in Figures 2, 4, and 5.

10)The axis of some graphs not always starts form zero; moreover, kindly use the same scale ranges while comparing male-female differences (For example, male FGF21 uses a different scale range from females) to make less challenging to compare the data.
We agree and have standardized axis origins (starting from zero where appropriate) and harmonized

scale ranges between male and female datasets to facilitate direct comparison across sexes where appropriate.

Really well done!

Selected points from cross-comment:

The main experimental concern to be addressed is from Rev 3 (Why were albumin-cre mouse not used as controls) that i largely share (well spotted!).

The rest of the comments relates to improvements in discussion/presentation of the data: I agree with other reviewers that a general issue with readability and figure presentation of the results makes the manuscript difficult to follow, and that discussion of the data and literature can be improved.
In the present manuscript, our focus is on the interaction between hepatocyte-derived FGF21 and the physiological response to fasting, rather than on the consequences of FGF21 deletion per se. Importantly, the basal phenotype under fed condition is unaffected by Cre-mediated deletion of *Fgf21*. Moreover, ALT levels were unchanged, and whole-genome expression analysis revealed no significant alterations apart from the expected robust reduction in *Fgf21*. Altogether, these observations indicate absence of toxicity and minimal off-target effects of Cre recombination in the liver. Therefore, we consider additional Cre controls neither standard nor scientifically necessary in the context of our study. We have addressed this point explicitly in our response to Referee #3. In addition, we have improved overall readability of the figures and manuscript. In agreement with the editor, we have changed the format of the manuscript from a Report to a full Research Article. This has allowed us to expand the discussion, to provide a more comprehensive view of prior studies, and to more explicitly position our work with existing literature.

Referee #3:

This paper was difficult to review because of combination of formatting and missing data. In addition the strategy for the specific studies included and an adequate interpretation are poorly described. While the authors argue that most studies evaluating the physiology of FGF21 in the fed and fasted state were performed in mice with total body deletion of FGF21 they are either unaware or not interested in discussing studies with liver specific FGF21 deletion. This includes a 2019 paper by Ameka et al as well as a 2020 manuscript on the role of hepatic FGF21 in mediating glycemic versus ketogenic responses by Watanabe et al. These previous publications would need to be directly addressed in the discussion.
We thank the reviewer for pointing out these important studies using liver-specific *Fgf21* deletion. We would like to clarify that we did not intend to overlook or disregard this literature. The studies by Ameka et al. (*Scientific Reports*, 2019) and Watanabe et al. (*Endocrine*, 2020) indeed address important aspects of hepatic FGF21 function; however, they were conducted in physiological contexts distinct from that of our work. Ameka *et al.* examined the role of hepatic FGF21 during cold exposure, while Watanabe *et al.* focused on responses to a ketogenic diet. In both cases, animals were not studied under fasting conditions, which is the central focus of our work. As a result, direct comparison is limited. Nevertheless, to better position our findings within the existing literature, we have now added these references to the Discussion.

An additional question is why were albumin-cre mouse not used as controls as cre mice can have unexpected metabolic phenotypes that may obscure the flox-cre mice with specific gene deletion? In the present manuscript, our focus is not on the consequences of *Fgf21* deletion per se, but on the

interaction between hepatocyte-derived FGF21 and the physiological response to fasting. Importantly, the basal phenotype (fed condition) is unaffected by Cre-mediated deletion of FGF21. Had Cre expression itself induced an effect, this would have been apparent in this condition.
Consistent with this approach, key publications in the field investigating liver-derived FGF21 have used floxed mice as controls rather than $Cre^{+}/^{-}$ animals (e.g. Markan et al., *Diabetes* 2014; Von Holstein-Rathlou et al., *Cell Metabolism* 2016). Likewise, the studies cited above by Referee #3 did not include $Cre^{+}/^{-}$ controls : Ameka et al. (*Scientific Reports*, 2019) used wild-type mice as controls for Alb-Cre–mediated *Fgf21* deletion; Watanabe et al. (*Endocrine*, 2020) used floxed mice as controls; and Von Holstein-Rathlou et al. (*Cell Metabolism*, 2016) similarly relied on floxed animals.
The inclusion of $Cre^{+}/^{-}$ controls would have been essential had we observed unexpected liver toxicity or broad changes in hepatic gene expression. However, we assessed ALT levels and other liver-specific toxicity markers, all of which were unchanged. Moreover, whole-genome expression analysis revealed no significant alterations apart from the expected robust reduction in *Fgf21*, indicating minimal off-target effects of Cre recombination in the liver.
Altogether, our control strategy is consistent with established practice in the field and is supported by both the literature and our experimental data.

With regard to poor formatting and missing data. The manuscript is not paginated, and the figures are not labelled. Where are figures 1-2 and three? They are referred to in the text but I do not see them anywhere within the printed manuscript which I have. For figures 4 and 5 for which there are figure legends, the legends do not match the text. For example the food choice figure legend for Figure 5 refers to panels A through F but only four panels are displayed in the figure which appears to be on the last page of the manuscript. A similar problem exists with Figure 4 where the number of panels are described as A to N but only panels a through H are shown. As this figure looks at changes in adipose tissue it is surprising that BAT is not examined as FGF21 has substantial effects on BAT both in the fasted state and when animals are eating a ketogenic diet. It seems that none of the authors paid adequate attention to the final assembly of the manuscript.
We regret any confusion caused by formatting issues in the submitted version of the manuscript. In the revised manuscript, we have now added page numbers throughout the text and ensured that all figures are clearly labelled, with figure numbers indicated on each figure.
Regarding the figures and legends, Figures 1–3 were present in the submitted manuscript and are correctly cited in the text. The apparent discrepancies between figure legends and displayed panels likely result from an incorrect association between figures and legends. In the revised version, we have carefully verified that each figure is matched with its corresponding legend and that panel numbering is consistent.
Concerning the analysis of brown adipose tissue (BAT), we would like to clarify that the effects of hepatocyte-specific *Fgf21* deletion on BAT responses to fasting were indeed examined. These data were originally presented in Figure EV8 and are included in the revised manuscript as Figure 5.

The figures labeled as expanded view figures are poorly described in the figure legends. For example for EV1 which is supposed to distinguish between liver floxed mice and liver mice with FGF 21 deletion of the assumptions regarding what is being shown in the figure legends on page 17 is unclear presumably fed and fasted are uh following the 20 hour fast described in the EV1 figure legend on page 15. It is difficult to determine what the expanded view figure should be matched with for example is the figure on page 19 which shows fed and fasted levels with both control and a ketogenic diet supposed to be Figure 1?
The confusion regarding the correspondence between Expanded View figures, main figures and legends again appears to result from an incorrect matching of figures and an incorrect association between figures and legends. In the revised version, we have carefully verified that each figure is labelled and matched with its corresponding legend. In addition, to address Referee#2 comment, we have now pooled all data obtained from the fasting experiment in females into a single figure (now

Appendix Figure S1). Similarly, all data obtained from the ketogenic diet experiment have been pooled into a single figure (now Appendix Figure S2).

As a specific question why is relative liver weight reported as relative liver weight as a percent of body weight and what I assume is extended figure one on page 17 rather absolute liver weight I do not find an indication that the weights of the mice were recorded and in fact this needs to be shown as data and liver weight should actually be shown as absolute rather than relative to total body weight another point is that while reportedly there are no differences between fed and fasted animals looking at figure I believe extended figure 1B hepatic triglycerides hepatic triglycerides appear to be higher in fasted FGF 21 knockout mice as uh this numbers of animals are small it's really difficult to tell if this is statistically significant

Relative liver weight was reported as a percentage of body weight to account for differences in overall body mass between fed and fasted animals, which is standard practice in metabolic studies. In accordance with *EMBO Reports* revision guidelines, we have provided the data source files associated with all main figures corresponding to the fasting experiment in males, including absolute liver weight and body weight. Body weight is reduced in response to fasting in both males (Figure 1) and females (previous Expended View Figure EV1 which is now Appendix Figure S1).

Regarding hepatic triglycerides, the group size (n = 8–10 mice per group) is sufficient for statistical analysis and is consistent with standards in the field. Appropriate statistical testing (two-way ANOVA followed by Šídák's multiple comparisons test, α = 0.05) was performed, and significant differences between fed and fasted conditions were detected in both genotypes (*p_adj = 0.0182 for *Fgf21*$^{hep+/+}$; ***p_adj = 0.005 for *Fgf21*$^{hep-/-}$) but no significant difference between fasted *Fgf21*$^{hep+/+}$ and fasted *Fgf21*$^{hep-/-}$ were observed (p_adj = 0.1183). In line with *EMBO Reports* revision guidelines, we have reported the exact adjusted P values for each relevant comparison in the figure legends. Data source files also contain all p values (genotype, nutritional condition, and interaction) for all figures. This ensures full statistical transparency.

On page what I believe is 21 differentially expressed genes by fasting and FGF 21 intact and FGF 21 liver specific knockout figure 1A it's really difficult to understand what is being shown on the basis of the way that the figure was formatted and showing the log full change also obscures potential differences between the groups.

In line with *EMBO Reports* revision guidelines, we have reported the exact adjusted P values for each relevant comparison in the figure legends. Data source files also contain all p values (genotype, nutritional condition, and interaction) for all figures. This ensures full statistical transparency.

Multiple panels in EV-5 show differences in gene expression (eg: Fut1) but the potential significance of these differences in the fasted state are not discussed.

In the original Figure EV5 (Figure EV2 in the revised version), among the genes shown, *Fut1* is the only one whose expression shows a significant difference between fasted *Fgf21*$^{hep+/+}$ and fasted *Fgf21*$^{hep-/-}$ (p_{adj}=0.0009). In addition, the interaction comparing (fed *Fgf21*$^{hep+/+}$_fed *Fgf21*$^{hep-/-}$)*vs*(fasted *Fgf21*$^{hep+/+}$ _fasted *Fgf21*$^{hep-/-}$) is not significant (p_{adj} = 0.58).

Fgf21 and *Fut1* are located in close genomic proximity on mouse chromosome 7, suggesting the presence of shared cis-regulatory elements. We therefore propose that Cre-mediated recombination at the *Fgf21* locus may alter the local chromatin environment, leading to a secondary reduction in *Fut1* expression.

Selected points from cross-comment:

Key missing experiments are as basic as food preference. The data presented is a shift in protein preference after fasting. While the authors refer to the studies of Laeger et al, they do not note the differences

Furthermore while the conclusion is that since no changes in fasting and responses to ketogenic diet are detected in liver specific FGF21 deleted mice and that therefore FGF21 must be acting through the brain no data specific to this conclusion are shown so this is in effect a confirmation of the previous studies by Laeger. Antonellis et al, Front Endocrinol 2016 confirmed that FGF21 null mice do not exhibit did not have an impaired response to fasting, another point in which the specific hepatic deletion of FGF21 becomes confirmatory, another report that is not referenced. Similarly Von Holstein-Rathlou showed that total body FGF21 deletion increased sweet preference.
Studies by Laeger et al. showed that hepatic FGF21 is induced by protein restriction and necessary for the metabolic adaptations to protein restriction, including increased food intake and changes in body weight. These studies clearly establish FGF21 as a key endocrine signal of protein restriction (Laeger et al., JCI, 2014 ; Laeger et al., Cell Reports, 2016). In contrast, our study demonstrates that although fasting also induces hepatic FGF21, this induction is not required for the classical metabolic adaptations to fasting. We show that liver-specific deletion of FGF21 does not affect systemic metabolic parameters, hepatic gene expression, or adipose tissue responses during fasting. These findings are consistent with previous reports showing preserved fasting responses in whole-body FGF21-deficient mice (Antonellis et al., Front Endocrinol 2016), which we now cite in the Discussion. Importantly, our work reveals that hepatic FGF21 is required for nutrient selection towards protein following fasting. To our knowledge, this specific role of liver-derived FGF21 in post-fasting protein preference has not been previously demonstrated.
Regarding the site of action, we agree with the reviewer that we do not directly demonstrate a central mechanism. However, the selective effect on nutrient preference, in the absence of metabolic alterations, strongly suggests a role for central FGF21 signaling, in line with previous studies showing central actions of FGF21 on nutrient and sweet preference (von Holstein-Rathlou et al., 2016 ; Hill et al, 2019). We already stated this in the previous version of the manuscript : « Our findings strongly support this model and suggest a liver-brain endocrine axis to mediate preference for proteins following a fasting period".
In agreement with the editor, we have changed the format of the manuscript from a Report to a full Research Article. This has allowed us to expand the discussion, to provide a more comprehensive view of prior studies, and to more explicitly position our work with existing literature.

Since circulating FGF21 is almost entirely derived from hepatocytes, deletion of the gene in hepatocytes would be expected to reproduce much of the total body deletion so much of the data "collapses" to a confirmatory study. The weight stability in the context of consumption of a ketogenic diet remains perplexing.

Finally with regard to impact -- much of the data presented is confirmatory of total body deletion because the "endocrine" functions of FGF21 are already well mapped to the hepatocyte release.
We would like to clarify why the liver-specific *Fgf21* deletion model provides novel and relevant findings rather than being just confirmatory.
First, while circulating FGF21 has indeed been shown to originate predominantly from hepatocytes (Markan et al, Diabetes, 2014), which we confirm in our study, FGF21 can also be produced by other tissues, including adipose tissue and pancreas, under specific physiological or pathological conditions (Dutchak et al, 2012 ; Fisher et al, 2012 ; Coate et al, 2017). Therefore, the use of a hepatocyte-specific *Fgf21* knockout remains highly relevant to selectively assess the contribution of liver-derived FGF21 during fasting without confounding effects of extrahepatic sources.
Second, previous studies investigating the role of FGF21 during fasting have primarily relied on whole-body knockout models or on pharmacological or transgenic overexpression of FGF21. The conclusions from these studies have not always been consistent, and the specific physiological role of endogenous FGF21 in response to fasting has remained unclear. In this context, liver-specific deletion of *Fgf21* represents a more physiological model to precisely assess the role of endogenous hepatic FGF21 during fasting.

Third, and most importantly, our study demonstrates for the first time that fasting-induced hepatic FGF21 is not required for metabolic adaptations to fasting, but is required after fasting to drive adaptive protein preference. This role of FGF21 has not been previously described and refines the current understanding of FGF21 biology, as also highlighted by Referee #1 and #2.

There is inconsistency with other reports with regard to the liver and ketogenic diets. For example weights of female mice consuming a ketogenic diet are shown to be the same (panel H -- unclear which figure) despite the fact that diets that reliable reduce ketosis ither prevent weight gain or are associated with weight loss in obese animals. Attenuated weight gain has been shown by multiple groups including Poplawski et al, PLos One, 2011, Jornayvaz et al AJP 2010.
As specified in the figure legend, the ketogenic diet in our study was administered for a short duration (9 days). In contrast, the studies cited by the reviewer typically involve much longer ketogenic feeding periods (five weeks or more), in which attenuated weight gain or weight loss has been reported, particularly in obese animals. We therefore believe that the lack of a significant difference in body weight observed in our study reflects differences in experimental duration rather than an inconsistency with the published literature.

Dear Dr. Fougerat

Thank you for the submission of your revised manuscript to EMBO reports. We have now received the set of referee reports that is copied below. We requested that Referee 2 comment on the completeness of your revision in response to Referee 3's comments.

As you can see, the referees find that the study is significantly improved during revision and recommend publication.

From the editorial side, there are just a few things that we need before we can proceed with the official acceptance of your study.

- Please move the Disclosure and Competing Interests Statement to be placed after the Acknowledgements section.

- Please adjust the author list so the format matches our system - First Name Last Name.

- Please provide an email address for Hervé Guillou on the title page of the manuscript.

- Please remove the Reagent and Tools Table from the manuscript file and upload it as an individual file. For more information, please check https://link.springer.com/journal/44318/submission-guidelines#structuredmethods and download the template for Reagent Table.

- Additionally, please update the table callout in the Reagent and Tools Table from Table2 to Appendix Table S2.

- Please note that EMBO press papers are accompanied online by:
A) a short (2 sentences) summary of the findings and their significance,
B) 2-5 short bullet points highlighting the key results, and
C) a synopsis image in .jpg or .png format that is exactly 550 pixels wide and 300-600 pixels high (the height is variable). Please note that the text needs to be legible at the final size. Please upload this information along with your revised manuscript (the text for A and B should be provided in one separate Word file uploaded as Cover Art/Synopsis Image).

- As a standard procedure, we edit the title and abstract of manuscripts to make them more accessible to a general readership. I have shortened your title to match our character limit (100 characters with spaces, 102 is acceptable) and adjusted three words in the abstract to maintain present tense. Please find the suggested versions below my signature and confirm whether they maintain the scientific intent of your originals.

- Please remove the DOI information from the reference list (except for pre-published papers that do not have assigned volume and page/elocator numbers)

- Our production/data editors have asked you to clarify several points in the figure legends (see below). Please incorporate these changes in the manuscript and return the revised file with tracked changes with your final manuscript submission.
- Please indicate the statistical test used for data analysis in the legend of figure 5E

We look forward to seeing a final version of your manuscript as soon as possible.

Kurt Weir
Editor
EMBO Reports

Hepatic FGF21 is not required for fasting metabolic responses but guides appetite post energy depletion

Fasting initiates a coordinated metabolic response to preserve energy balance. As glycogen stores are depleted, the body transitions to mobilizing fatty acids from adipose tissue and generating ketone bodies in the liver to sustain the function of vital organs. A network of hormonal signals and transcriptional programs coordinate these adaptations. Among these, the hepatokine fibroblast growth factor 21 (FGF21) is strongly upregulated during fasting and has been proposed as a key mediator of the fasting response. To investigate the physiological functions of FGF21, we study mice with hepatocyte-specific deletion of Fgf21. Although the liver is the primary source of circulating FGF21 during fasting, its absence in hepatocytes does not alter typical fasting-induced gene expression or key metabolic pathways such as hepatic gluconeogenesis, adipose tissue lipolysis, or ketone production. Instead, we uncover a distinct role for FGF21 in promoting protein appetite following a fast. These findings challenge the conventional view of hepatocyte-produced FGF21 as a fasting-acting hormone and reveal a more specialized function in guiding nutrient selection after energy depletion.

Referee #1:

The manuscript has been appropriately revised.

Referee #2:

The authors have proactively approached the revision and fully addressed my concerns. No additional comments from my side.

Referee #2 Comments on revisions in response to Referee #3:

In my opinion all the comments have been diligently and proactively addressed with supporting data/literature/discussion.

Referee #3:

The manuscript is much improved with appropriate pagination, citation of figures which made it easier to review. The addition of suggested references is appreciated.

A key issue that came up re-evaluating the manuscript is that the relevance of FGF21 as a fasting factor was not considered to be a feature of human biology. The only reports of increased circulating FGF21 in humans came from either sever or 10 days fasting and was likely attributed to autophagy/damage to hepatocytes. Conventional fasting overnight or even for 3 days did not increase FGF21 in humans.

From the perspective of the manuscript the title should reflect the fact that this is a mouse study. In addition the discussion could include a paragraph on the differences/similarities between mice and humans. This would include lack of an effect with fasting or ketogenic diet in humans compared to mice but similarities with ingestion of fructose and alcohol. In that vein the FGF21 therapies for human disease are aimed at the liver, particularly MASH and cirrhosis as opposed to metabolic disease. I think this is an important addition.

With regard to weight loss and ketogenic diet 5333 has almost no protein whereas the diet used here has sufficient proteing which could affect weight loss compared to other studies.

One minior point -- the micrographs 1D and 5B are extremely difficult to visualize, even with increased magnification on the electronic diet. Are micrographs with higher magnification available?

The authors have addressed all editorial requests.

Dr. Anne Fougerat
INRAE
Toxalim
Toulouse
France

Dear Dr. Fougerat,

I am pleased to inform you that your manuscript has been accepted for publication in EMBO reports. Your manuscript will be processed for publication by EMBO Press. It will be copy edited and you will receive page proofs prior to publication. Please note that you will be contacted by Springer Nature Author Services to complete licensing and payment information.

You may qualify for financial assistance for your publication charges - either via a Springer Nature fully open access agreement or an EMBO initiative. Check your eligibility: https://link.springer.com/journal/44319/how-to-publish-with-us

Should you be planning a Press Release on your article, please get in contact with embo_production@springernature.com as early as possible in order to coordinate publication and release dates.

If you have any questions, please do not hesitate to contact the Editorial Office. Thank you for your contribution to EMBO Reports.

Yours sincerely,

Kurt Weir
Editor
EMBO Reports

>>> Please note that it is EMBO Reports policy for the transcript of the editorial process (containing referee reports and your response letter) to be published as an online supplement to each paper. If you do NOT want this, you will need to inform the Editorial Office via email immediately. More information is available here: https://link.springer.com/partners/embo-press/editorial-policies#Peer%20review

EMBO Press Author Checklist

Corresponding Author Name: Anne Fougerat	
Journal Submitted to: EMBO Reports	
Manuscript Number: EMBOR-2025-63031	

USEFUL LINKS FOR COMPLETING THIS FORM
The EMBO Journal - Author Guidelines
EMBO Reports - Author Guidelines
Molecular Systems Biology - Author Guidelines
EMBO Molecular Medicine - Author Guidelines

Reporting Checklist for Life Science Articles (updated January
This checklist is adapted from Materials Design Analysis Reporting (MDAR) Checklist for Authors. MDAR establishes a minimum set of requirements in transparent reporting in the life sciences (see Statement of Task: 10.31222/osf.io/9sm4x). Please follow the journal's guidelines in preparing your
Please note that a copy of this checklist will be published alongside your article.

Abridged guidelines for figures
1. Data
The data shown in figures should satisfy the following conditions:
- the data were obtained and processed according to the field's best practice and are presented to reflect the results of the experiments in an accurate and unbiased manner.
- ideally, figure panels should include only measurements that are directly comparable to each other and obtained with the same assay.
- plots include clearly labeled error bars for independent experiments and sample sizes. Unless justified, error bars should not be shown for technical
- if n<5, the individual data points from each experiment should be plotted. Any statistical test employed should be justified.
- Source Data should be included to report the data underlying figures according to the guidelines set out in the authorship guidelines on Data

2. Captions
Each figure caption should contain the following information, for each panel where they are relevant:
- a specification of the experimental system investigated (eg cell line, species name).
- the assay(s) and method(s) used to carry out the reported observations and measurements.
- an explicit mention of the biological and chemical entity(ies) that are being measured.
- an explicit mention of the biological and chemical entity(ies) that are altered/varied/perturbed in a controlled manner.
- the exact sample size (n) for each experimental group/condition, given as a number, not a range;
- a description of the sample collection allowing the reader to understand whether the samples represent technical or biological replicates (including how many animals, litters, cultures, etc.).
- a statement of how many times the experiment shown was independently replicated in the laboratory.
- definitions of statistical methods and measures:
  - common tests, such as t-test (please specify whether paired vs. unpaired), simple χ2 tests, Wilcoxon and Mann-Whitney tests, can be unambiguously identified by name only, but more complex techniques should be described in the methods section;
  - are tests one-sided or two-sided?
  - are there adjustments for multiple comparisons?
  - exact statistical test results, e.g., P values = x but not P values < x;
  - definition of 'center values' as median or average;
  - definition of error bars as s.d. or s.e.m.

Please complete ALL of the questions below.
Select "Not Applicable" only when the requested information is not relevant for your study.

Materials

Newly Created Materials	Information included in the manuscript?	In which section is the information available? (Reagents and Tools Table, Materials and Methods, Figures, Data Availability Section)
New materials and reagents need to be available; do any restrictions apply?	Not Applicable	

Antibodies	Information included in the manuscript?	In which section is the information available? (Reagents and Tools Table, Materials and Methods, Figures, Data Availability Section)
For **antibodies** provide the following information:<br>- Commercial antibodies: RRID (if possible) or supplier name, catalogue number and or/clone number<br>- Non-commercial: RRID or citation	Not Applicable	

DNA and RNA sequences	Information included in the manuscript?	In which section is the information available? (Reagents and Tools Table, Materials and Methods, Figures, Data Availability Section)
**Short novel DNA or RNA including primers, probes**: provide the sequences.	Yes	Appendix Table S2

Cell materials	Information included in the manuscript?	In which section is the information available? (Reagents and Tools Table, Materials and Methods, Figures, Data Availability Section)
**Cell lines:** Provide species information, strain. Provide accession number in repository **OR** supplier name, catalog number, clone number, and/**OR** RRID.	Not Applicable	
**Primary cultures:** Provide species, strain, sex of origin, genetic modification status.	Not Applicable	
Report if the cell lines were recently **authenticated** (e.g., by STR profiling) and tested for mycoplasma contamination.	Not Applicable	

Experimental animals	Information included in the manuscript?	In which section is the information available? (Reagents and Tools Table, Materials and Methods, Figures, Data Availability Section)
**Laboratory animals or Model organisms:** Provide species, strain, sex, age, genetic modification status. Provide accession number in repository **OR** supplier name, catalog number, clone number, **OR** RRID.	Yes	Reagents and Tools Table
**Animal observed in or captured from the field:** Provide species, sex, and age where possible.	Not Applicable	
Please detail **housing and husbandry conditions**.	Yes	Materials and Methods

Plants and microbes	Information included in the manuscript?	In which section is the information available? (Reagents and Tools Table, Materials and Methods, Figures, Data Availability Section)
**Plants:** provide species and strain, ecotype and cultivar where relevant, unique accession number if available, and source (including location for collected wild specimens).	Not Applicable	
**Microbes:** provide species and strain, unique accession number if available, and source.	Not Applicable	

Human research participants	Information included in the manuscript?	In which section is the information available? (Reagents and Tools Table, Materials and Methods, Figures, Data Availability Section)
If collected and within the bounds of privacy constraints report on age, sex and gender or ethnicity for all study participants.	Not Applicable	

Core facilities	Information included in the manuscript?	In which section is the information available? (Reagents and Tools Table, Materials and Methods, Figures, Data Availability Section)
If your work benefited from core facilities, was their service mentioned in the acknowledgments section?	Yes	Acknowledgments

Design

Study protocol	Information included in the manuscript?	In which section is the information available? (Reagents and Tools Table, Materials and Methods, Figures, Data Availability Section)
If study protocol has been **pre-registered, provide DOI in the manuscript**. For clinical trials, provide the trial registration number **OR** cite DOI.	Not Applicable	
Report the **clinical trial registration number** (at ClinicalTrials.gov or equivalent), where applicable.	Not Applicable	

Laboratory protocol	Information included in the manuscript?	In which section is the information available? (Reagents and Tools Table, Materials and Methods, Figures, Data Availability Section)
Provide DOI OR other citation details if **external detailed step-by-step protocols** are available.	Not Applicable	

Experimental study design and statistics	Information included in the manuscript?	In which section is the information available? (Reagents and Tools Table, Materials and Methods, Figures, Data Availability Section)
Include a statement about **sample size** estimate even if no statistical methods were used.	Yes	Figures legends
Were any steps taken to minimize the effects of subjective bias when allocating animals/samples to treatment (e.g. **randomization procedure**)? If yes, have they been described?	Yes	Materials and Methods
Include a statement about **blinding** even if no blinding was done.	Not Applicable	
Describe **inclusion/exclusion criteria** if samples or animals were excluded from the analysis. Were the criteria pre-established?<br><br>If sample or data points were omitted from analysis, report if this was due to attrition or intentional exclusion and provide justification.	Not Applicable	
For every figure, are **statistical tests** justified as appropriate? Do the data meet the assumptions of the tests (e.g., normal distribution)? Describe any methods used to assess it. Is there an estimate of variation within each group of data? Is the variance similar between the groups that are being statistically compared?	Yes	Figures legends; Source data

Sample definition and in-laboratory replication	Information included in the manuscript?	In which section is the information available? (Reagents and Tools Table, Materials and Methods, Figures, Data Availability Section)
In the figure legends: state number of times the experiment was **replicated** in laboratory.	Not Applicable	
In the figure legends: define whether data describe **technical or biological replicates**.	Yes	Figures legends

Ethics

Ethics	Information included in the manuscript?	In which section is the information available? (Reagents and Tools Table, Materials and Methods, Figures, Data Availability Section)
Studies involving **human participants**: State details of **authority granting ethics approval** (IRB or equivalent committee(s), provide reference number for approval.	Not Applicable	
Studies involving **human participants**: Include a statement confirming that **informed consent** was obtained from all subjects and that the experiments conformed to the principles set out in the WMA Declaration of Helsinki and the Department of Health and Human Services Belmont Report.	Not Applicable	
Studies involving **human participants:** For publication of **patient photos**, include a statement confirming that consent to publish was obtained.	Not Applicable	
Studies involving experimental **animals**: State details of **authority granting ethics approval** (IRB or equivalent committee(s), provide reference number for approval. Include a statement of compliance with ethical regulations.	Yes	Reagents and Tools Table; Materials and Methods
Studies involving **specimen and field samples:** State if relevant **permits** obtained, provide details of authority approving study; if none were required, explain why.	Not Applicable	

Dual Use Research of Concern (DURC)	Information included in the manuscript?	In which section is the information available? (Reagents and Tools Table, Materials and Methods, Figures, Data Availability Section)
Could your study fall under dual use research restrictions? Please check biosecurity documents and list of **select agents and toxins** (CDC): https://www.selectagents.gov/sat/list.htm	Not Applicable	
If you used a select agent, is the security level of the lab appropriate and reported in the manuscript?	Not Applicable	
If a study is subject to dual use research of concern regulations, is the name of the **authority granting approval and reference number** for the regulatory approval provided in the manuscript?	Not Applicable	

Reporting

The MDAR framework recommends adoption of discipline-specific guidelines, established and endorsed through community initiatives. Journals have their own policy about requiring specific guidelines and recommendations to complement MDAR.

Adherence to community standards	Information included in the manuscript?	In which section is the information available? (Reagents and Tools Table, Materials and Methods, Figures, Data Availability Section)
State if relevant guidelines or checklists (e.g., **ICMJE, MIBBI, ARRIVE, PRISMA**) have been followed or provided.	Not Applicable	
For **tumor marker prognostic studies,** we recommend that you follow the **REMARK** reporting guidelines (see link list at top right). See author guidelines, under 'Reporting Guidelines'. Please confirm you have followed these guidelines.	Not Applicable	
For **phase II and III randomized controlled trials**, please refer to the **CONSORT** flow diagram (see link list at top right) and submit the CONSORT checklist (see link list at top right) with your submission. See author guidelines, under 'Reporting Guidelines'. Please confirm you have submitted this list.	Not Applicable	

Data Availability

Data availability	Information included in the manuscript?	In which section is the information available? (Reagents and Tools Table, Materials and Methods, Figures, Data Availability Section)
Have **primary datasets** been deposited according to the journal's guidelines (see 'Data Deposition' section) and the respective accession numbers provided in the Data Availability Section?	Yes	Data Availability Section
Were **human clinical and genomic datasets** deposited in a public access-controlled repository in accordance to ethical obligations to the patients and to the applicable consent agreement?	Not Applicable	
Are **computational models** that are central and integral to a study available without restrictions in a machine-readable form? Were the relevant accession numbers or links provided?	Not Applicable	
If publicly available data were reused, provide the respective **data citations in the reference list**.	Yes	Materials and Methods
